# Supplementary material for: Quantifying patient preferences for symptomatic breast clinic referral: a decision analysis study
Source: BMJ Open. 2018 May 31;8(5):e017286. doi: 10.1136/bmjopen-2017-017286 (PMC5988058; doi:10.1136/bmjopen-2017-017286)
Supplement: Supplementary file 1 [file bmjopen-2017-017286supp001.pdf]

HRB Centre for Primary Care Research

Welcome to the Breast Cancer Preference Study

Please enter your participant number below

Start Study

HRB Centre for Primary Care Research

Please complete the paper based questionnaire before proceeding

Completed

HRB Centre for Primary Care Research

This is a study aimed at understanding womens' perceptions of breast cancer. Women with certain risk factors, signs and symptoms are more likely than others to develop breast cancer.

We will present you with several health scenarios, and ask you to choose between different states of health.

Next

Scenario

You have presented to your GP with symptom(s) of breast cancer.
Your GP will take a history of your symptoms, and perform a breast examination. They may also perform any other examinations/tests that they feel are necessary (listen to your heart, take blood, etc.).
Based on history and examination findings, your GP will either recommend a "watchful waiting" programme, or triple assessment at a specialist breast clinic. However, it is your decision which option you prefer.

Definitions

Beside you are definitions for terms that you will come across in the scenarios. Please read them now, but you will also have a copy of them beside you to refer to at any point. Any word in **blue** in the scenarios has a definition provided.

How will we measure your preferences?

We will use 2 methods (the VAS and standard gamble) to assess how you feel about each of the 11 health states. The health states will be presented to you in random order.
An explanation of each of the methods will follow. You also have laminated cards in front of you with the same information.
You can refer to these at any time that you wish.

Visual Analogue Scale - VAS

The Visual Analogue Scale is a scale from 0-100, with 0 being the worst health scenario you can think of (death), and 100 being the best health scenario (perfect health).
We will ask you to read each health care scenario individually, and then to place it where you think it fits along the scale.
You do not need even gaps between scenarios on the scale, and you can place two or more scenarios on the same number if you wish.
Scale: 0-100

Please include VAS scale here

36 people.
Scen 1-5
Type Asse.
6-11
Watch Wil

Standard Gamble.

You will be presented with 11 different health state scenarios, each scenario represents your current permanent health state. We will ask you to make a gamble between your current health state with two other health states (perfect health or death). I want you to imagine that this carries a certain chance of you living the rest of your life in perfect health and a certain chance of you dying painlessly tomorrow.

It is important to remember your current health scenario, and your current symptoms, treatments, side-effects and prognosis. You will be given different chances of perfect health versus death, against the current scenario, and keep gambling until you reach a point where you cannot choose between the 2 options.

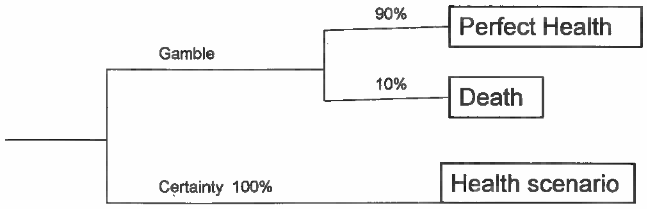

Example- Standard Gamble

Imagine you have a heart condition. You have a choice between taking medication which has unpleasant side effects or taking no medication.

If you had to choose between taking this medication with side-effects for the rest of your life, or taking no medication and have perfect health, but with the risk of dying, what risk of death would you take to avoid the medication and side effects?

Another way of putting it is - what risk of dying painlessly tomorrow would you be willing to accept (if you didn't take the drugs) before you change your mind and decide you would rather take them?

•If offered a 90% chance of perfect health, no drugs, no side effects, but with a 10% risk of instant death, would you take it?

•If offered a 70% chance of perfect health, no drugs, no side effects, but with a 30% risk of instant death, would you take it?

Below, you will see 2 buttons. When you click on the right button, the chances of perfect health and death vary, these are examples to help you understand. In the text box below, please enter in the risk of death that you are willing to take.

no drugs, no side effects

Live in current Health State.	Versus	50% chance of perfect health and 50% chance of death.

instant

[ ] % chance of death

Enter

The Study

We are going to present you with 11 possible health scenarios in a random order - please follow the instructions and if you have any questions please ask.

Definitions have been provided for any word or phrase in **blue**.

1.0069

Health State 1: Referred to breast clinic for triple assessment - Stage 1 Breast Cancer

What it means:
You were referred for **triple assessment** and breast cancer has been diagnosed that is confined to the breast and may involve a small number of **lymph nodes**. This is an early stage of breast cancer.

Physical Consequences:
- You have undergone **triple assessment.**
- You will have surgery (**lumpectomy, mastectomy, lymph node removal**).
- **Chemotherapy** and/or **radiation therapy** to prevent the cancer from coming back.
- **Hormonal therapy** or **biological therapy** may be given, depending on the type of cancer cell.

Emotional Consequences:
- Fear over investigations and treatments (pain, side-effects).
- Anxiety or depression associated with diagnosis.
- Worry that cancer may spread or will not be fully cured.

VAS Scale
Assume that you are currently experiencing the health state you just read. On a scale of 0 to 100 where 0 is death and 100 perfect health where would you rate this health state. Please use the scale below.
When you are happy with your selection please click 'Completed'.

Health State 1: Referred to breast clinic for triple assessment - Stage 1 Breast Cancer

What it means:
You were referred for **triple assessment** and breast cancer has been diagnosed that is confined to the breast and may involve a small number of **lymph nodes**. This is an early stage of breast cancer.

Physical Consequences:
- You have undergone **triple assessment.**
- You will have surgery (**lumpectomy, mastectomy, lymph node removal**).
- **Chemotherapy** and/or **radiation therapy** to prevent the cancer from coming back.
- **Hormonal therapy** or **biological therapy** may be given, depending on the type of cancer cell.

Emotional Consequences:
- Fear over investigations and treatments (pain, side-effects).
- Anxiety or depression associated with diagnosis.
- Worry that cancer may spread or will not be fully cured.

Standard Gamble
You have the opportunity to gamble your current health state (as described above) for perfect health, but you also risk death if you take the gamble. It is important to remember about your current health scenario, and your current symptoms, treatments, side-effects and prognosis. Below, you will see 2 buttons. When you click on the right button, the chances of perfect health and death vary, these are examples to help you understand. In the text box below, please enter in the risk of death that you are willing to take in order to avoid this health state.

\ type

Health State 2: Referred to breast clinic for triple assessment - Stage 2 Breast Cancer

What it means:
Triple assessment has found cancer that is contained within the breast. It may also be in surrounding **lymph nodes**.

Physical Consequences:
- You have undergone **triple assessment.**
- You will have surgery **(lumpectomy, mastectomy, lymph node removal)**.
- You will have **radiation therapy.**
- You may have **chemotherapy** and/or **hormonal therapy,** depending on the type of cancer.

Emotional Consequences:
- Fear over investigations and treatments (pain, side-effects).
- Anxiety or depression associated with diagnosis.
- Fear of recurrence.

VAS Scale
Assume that you are currently experiencing the health state you just read. On a scale of 0 to 100 where 0 is death and 100 perfect health where would you rate this health state. Please use the scale below. When you are happy with your selection please click 'Completed'.

Health State 2: Referred to breast clinic for triple assessment; Stage 2 Breast Cancer

What it means:
Triple assessment has found cancer that is contained within the breast. It may also be in surrounding **lymph nodes**.

Physical Consequences:
- You have undergone **triple assessment.**
- You will have surgery **(lumpectomy, mastectomy, lymph node removal)**.
- You will have **radiation therapy.**
- You may have **chemotherapy** and/or **hormonal therapy,** depending on the type of cancer.

Emotional Consequences:
- Fear over investigations and treatments (pain, side-effects).
- Anxiety or depression associated with diagnosis.
- Fear of recurrence.

Standard Gamble
You have the opportunity to gamble your current health state (as described above) for perfect health, but you also risk death if you take the gamble. It is important to remember about your current health scenario, and your current symptoms, treatments, side-effects and prognosis. Below, you will see 2 buttons. When you click on the right button, the chances of perfect health and death vary, these are examples to help you understand. In the text box below, please enter in the risk of death that you are willing to take in order to avoid this health state.

Health State 3: Referred to breast clinic for triple assessment - Stage 3 Cancer

What it means:
You were referred for **triple assessment** and cancer was found in the breast, it has spread into surrounding tissues and many **lymph nodes** are involved.

Physical consequences:
• You have undergone **triple assessment.**
•You will have a surgery (**lumpectomy, mastectomy, lymph node removal**).
•You will probably have **radiation therapy** and/or **chemotherapy.**
•You may have **hormonal** or **biological therapy**, depending on the type of cancer cells.

Emotional consequences:
• Fear over investigations and treatments (pain, side-effects).
• Poorer prognosis, worry about survival.
• Fear of recurrence.

VAS Scale
Assume that you are currently experiencing the health state you just read. On a scale of 0 to 100 where 0 is death and 100 perfect health where would you rate this health state. Please use the scale below.
When you are happy with your selection please click 'Completed'.

Health State 3: Referred to breast clinic for triple assessment - Stage 3 Cancer

What it means:
You were referred for **triple assessment** and cancer was found in the breast, it has spread into surrounding tissues and many **lymph nodes** are involved.

Physical consequences:
• You have undergone **triple assessment.**
•You will have a surgery (**lumpectomy, mastectomy, lymph node removal**).
•You will probably have **radiation therapy** and/or **chemotherapy.**
•You may have **hormonal** or **biological therapy**, depending on the type of cancer cells.

Emotional consequences:
• Fear over investigations and treatments (pain, side-effects).
• Poorer prognosis, worry about survival .
• Fear of recurrence.

Standard Gamble
You have the opportunity to gamble your current health state (as described above) for perfect health, but you also risk death if you take the gamble. It is important to remember about your current health scenario, and your current symptoms, treatments, side-effects and prognosis. Below, you will see 2 buttons. When you click on the right button, the chances of perfect health and death vary, these are examples to help you understand. In the text box below, please enter in the risk of death that you are willing to take in order to avoid this health state.

Health State 4: Referred to breast clinic for triple assessment - Stage 4 Breast Cancer found

What it means:
You are referred for **triple assessment**, and cancer is found in your breast, which has also spread to one or more organs in the body. At this stage the cancer cannot be cured, but treatment can slow down the disease and let you live longer.

Physical consequences:
- You have undergone **triple assessment**.
- The main treatment is **chemotherapy.**
- You may have surgery or **radiation therapy** to treat pain and other symptoms.
- Possible **hormonal and/or biological therapy**, depending on type of cancer.

Emotional consequences:
- Fear due to poor prognosis and treatments.
- Fear of dying.

VAS Scale
Assume that you are currently experiencing the health state you just read. On a scale of 0 to 100 where 0 is death and 100 perfect health where would you rate this health state. Please use the scale below. When you are happy with your selection please click 'Completed'.

Health State 4: Referred to breast clinic for triple assessment - Stage 4 Breast Cancer found

What it means:
You are referred for **triple assessment**, and cancer is found in your breast, which has also spread to one or more organs in the body. At this stage the cancer cannot be cured, but treatment can slow down the disease and let you live longer.

Physical consequences:
- You have undergone **triple assessment**.
- The main treatment is **chemotherapy.**
- You may have surgery or **radiation therapy** to treat pain and other symptoms.
- Possible **hormonal and/or biological therapy**, depending on type of cancer.

Emotional consequences:
- Fear due to poor prognosis and treatments.
- Fear of dying.

Standard Gamble
You have the opportunity to gamble your current health state (as described above) for perfect health, but you also risk death if you take the gamble. It is important to remember about your current health scenario, and your current symptoms, treatments, side-effects and prognosis. Below, you will see 2 buttons. When you click on the right button, the chances of perfect health and death vary, these are examples to help you understand. In the text box below, please enter in the risk of death that you are willing to take in order to avoid this health state.

Health State 5: Referred to breast clinic for triple assessment - No cancer found

What it means:
You were referred for **triple assessment** and no cancer was found.

Physical consequences:
•You have undergone **triple assessment**.

Emotional consequences:
• Fear of investigations.
• Fear that the results were wrong and that you could still have cancer.

VAS Scale
Assume that you are currently experiencing the health state you just read. On a scale of 0 to 100 where 0 is death and 100 perfect health where would you rate this health state. Please use the scale below.
When you are happy with your selection please click 'Completed'.

Health State 5: Referred to breast clinic for triple assessment - No cancer found

What it means:
You were referred for **triple assessment** and no cancer was found.

Physical consequences:
•You have undergone **triple assessment**.

Emotional consequences:
•Fear of investigations
•Fear that the results might be wrong and that you could still have cancer.

Standard Gamble
You have the opportunity to gamble your current health state (as described above) for perfect health, but you also risk death if you take the gamble. It is important to remember about your current health scenario, and your current symptoms, treatments, side-effects and prognosis. Below, you will see 2 buttons. When you click on the right button, the chances of perfect health and death vary, these are examples to help you understand. In the text box below, please enter in the risk of death that you are willing to take in order to avoid this health state.

Health State 6: Watchful waiting, recurrence/worsening of symptoms, referred to breast clinic for triple assessment - Stage 1 Breast Cancer found

What it means
After a period of **watchful waiting** with your GP, your symptoms were not resolving/becoming worse. You were referred for **triple assessment** and breast cancer has been diagnosed that is confined to the breast and may involve a small number of **lymph nodes**. This is an early stage of breast cancer.

Physical consequences:
- You have undergone **triple assessment**.
- You will have surgery (**lumpectomy, mastectomy, lymph node removal**).
- **Chemotherapy** and/or **radiation therapy** to prevent the cancer from coming back.
- **Hormonal therapy** or **biological therapy** may be given, depending on the type of cancer cells.

Emotional Consequences:
- Anger/worry about delay in diagnosis.
- Fear over investigations and treatments (pain, side-effects).
- Anxiety or depression associated with diagnosis.
- Worry that cancer may spread or will not be fully cured.

VAS Scale
Assume that you are currently experiencing the health state you just read. On a scale of 0 to 100 where 0 is death and 100 perfect health where would you rate this health state. Please use the scale below. When you are happy with your selection please click 'Completed

Health State 6: Watchful waiting, recurrence/worsening of symptoms, referred to breast clinic for triple assessment - Stage 1 Breast Cancer found

What it means
After a period of **watchful waiting** with your GP, your symptoms were not resolving/becoming worse. You were referred for **triple assessment** and breast cancer has been diagnosed that is confined to the breast and may involve a small number of **lymph nodes**. This is an early stage of breast cancer.

Physical consequences:
- You have undergone **triple assessment**.
- You will have surgery (**lumpectomy, mastectomy, lymph node removal**).
- **Chemotherapy** and/or **radiation therapy** to prevent the cancer from coming back.
- **Hormonal therapy** or **biological therapy** may be given, depending on the type of cancer cells.

Emotional Consequences:
- Anger/worry about delay in diagnosis.
- Fear over investigations and treatments (pain, side-effects).
- Anxiety or depression associated with diagnosis.
- Worry that cancer may spread or will not be fully cured

Standard Gamble
You have the opportunity to gamble your current health state (as described above) for perfect health, but you also risk death if you take the gamble. It is important to remember about your current health scenario, and your current symptoms, treatments, side-effects and prognosis. Below, you will see 2 buttons. When you click on the right button, the chances of perfect health and death vary, these are examples to help you understand. In the text box below, please enter in the risk of death that you are willing to take in order to avoid this health state.

Health State 7: Watchful waiting, recurrence/worsening of symptoms, referred to breast clinic for triple assessment - Stage 2 Breast Cancer found

What it means:
After a period of **watchful waiting**, your symptoms were not resolving/became worse. You were referred for **triple assessment**, and cancer was found, which is contained within the breast. It may also be in surrounding **lymph nodes.**

Physical consequences:
- You have undergone **triple assessment**.
- You will have surgery (**lumpectomy, mastectomy, lymph node removal**).
- You will have **radiation therapy.**
- You may have **chemotherapy**, and/or **hormonal therapy,** depending on the type of cancer

Emotional consequences:
- Anger/worry about delay in diagnosis.
- Fear over investigations and treatments (pain, side-effects).
- Anxiety or depression due to cancer diagnosis.
- Fear of recurrence.

<u>VAS Scale</u>
Assume that you are currently experiencing the health state you just read. On a scale of 0 to 100 where 0 is death and 100 perfect health where would you rate this health state. Please use the scale below.
When you are happy with your selection please click 'Completed

Health State 7: Watchful waiting, recurrence/worsening of symptoms, referred to breast clinic for triple assessment - Stage 2 Breast Cancer found

What it means:
After a period of **watchful waiting**, your symptoms were not resolving/became worse. You were referred for **triple assessment**, and cancer was found, which is contained within the breast. It may also be in surrounding **lymph nodes.**

Physical consequences:
- You have undergone **triple assessment**.
- You will have surgery (**lumpectomy, mastectomy, lymph node removal**).
- You will have **radiation therapy.**
- You may have **chemotherapy**, and/or **hormonal therapy,** depending on the type of cancer.

Emotional consequences:
- Anger/worry about delay in diagnosis.
- Fear over investigations and treatments (pain, side-effects).
- Anxiety or depression due to cancer diagnosis.
- Fear of recurrence.

<u>Standard Gamble</u>
You have the opportunity to gamble your current health state (as described above) for perfect health, but you also risk death if you take the gamble. It is important to remember about your current health scenario, and your current symptoms, treatments, side-effects and prognosis. Below, you will see 2 buttons. When you click on the right button, the chances of perfect health and death vary, these are examples to help you understand. In the text box below, please enter in the risk of death that you are willing to take in order to avoid this health state.

Health State 8: Watchful waiting, recurrence/worsening of symptoms, referred to breast clinic for triple assessment - Stage 3 Breast Cancer found

What it means:
After a period of **watchful waiting**, your symptoms have not resolved/become worse. You are referred for **triple assessment**. Cancer is found in the breast, it has spread into surrounding tissues, and many **lymph nodes** are involved.

Physical consequences:
- You have undergone **triple assessment.**
- You will have a surgery (**lumpectomy, mastectomy, lymph node removal**).
- You will probably have **radiation therapy** and/or **chemotherapy.**
- You may have **hormonal** or **biological therapy**, depending on the type of cancer cells.

Emotional consequences:
- Anger/worry about delay in diagnosis.
- Fear over investigations and treatments (pain, side-effects).
- Poorer prognosis, worry about survival.
- Fear of recurrence.

VAS Scale
Assume that you are currently experiencing the health state you just read. On a scale of 0 to 100 where 0 is death and 100 perfect health where would you rate this health state. Please use the scale below. When you are happy with your selection please click 'Completed

Health State 8: Watchful waiting, recurrence/worsening of symptoms, referred to breast clinic for triple assessment - Stage 3 Breast Cancer found

What it means:
After a period of **watchful waiting**, your symptoms have not resolved/become worse. You are referred for **Triple assessment**. Cancer is found in the breast, it has spread into surrounding tissues, and many **lymph nodes** are involved.

Physical consequences:
- You have undergone **triple assessment.**
- You will have a surgery (**lumpectomy, mastectomy, lymph node removal**).
- You will probably have **radiation therapy** and/or **chemotherapy.**
- You may have **hormonal** or **biological therapy**, depending on the type of cancer cells.

Emotional consequences:
- Anger/worry about delay in diagnosis.
- Fear over investigations and treatments (pain, side-effects).
- Poorer prognosis, worry about survival.
- Fear of recurrence.

Standard Gamble
You have the opportunity to gamble your current health state (as described above) for perfect health, but you also risk death if you take the gamble. It is important to remember about your current health scenario, and your current symptoms, treatments, side-effects and prognosis. Below, you will see 2 buttons. When you click on the right button, the chances of perfect health and death vary, these are examples to help you understand. In the text box below, please enter in the risk of death that you are willing to take in order to avoid this health state.

Health State 9: Watchful waiting, recurrence/worsening of symptoms, referred to breast clinic for triple assessment - Stage 4 Breast Cancer found

What it means:
After a period of **watchful waiting** with your GP, your symptoms have not resolved/became worse. You are referred for **triple assessment**, and cancer is found in your breast, which has also spread to one or more organs in the body. At this stage the cancer cannot be cured, but treatment can slow down the disease and let you live longer.

Physical consequences:
- You have undergone **triple assessment**.
- The main treatment is **chemotherapy.**
- You may have surgery or **radiation therapy** to treat pain and other symptoms.
- Possible **hormonal** and/or **biological therapy**, depending on type of cancer.

Emotional Consequences:
- Anger/worry about delay in diagnosis.
- Fear due to poor prognosis and treatments.
- Fear of dying.

VAS Scale
Assume that you are currently experiencing the health state you just read. On a scale of 0 to 100 where 0 is death and 100 perfect health where would you rate this health state. Please use the scale below. When you are happy with your selection please click 'Completed'.

Health State 1
0___|_____________________100

Health State 9: Watchful waiting, recurrence/worsening of symptoms, referred to breast clinic for triple assessment - Stage 4 Breast Cancer found

What it means:
After a period of **watchful waiting** with your GP, your symptoms have not resolved/became worse. You are referred for **triple assessment**, and cancer is found in your breast, which has also spread to one or more organs in the body. At this stage the cancer cannot be cured, but treatment can slow down the disease and let you live longer.

Physical consequences:
- You have undergone **triple assessment**.
- The main treatment is **chemotherapy.**
- You may have surgery or **radiation therapy** to treat pain and other symptoms.
- Possible **hormonal** and/or **biological therapy**, depending on type of cancer.

Emotional Consequences:
- Anger/worry about delay in diagnosis.
- Fear due to poor prognosis and treatments.
- Fear of dying.

Standard Gamble.
You have the opportunity to gamble your current health state (as described above) for perfect health, but you also risk death if you take the gamble. It is important to remember about your current health scenario, and your current symptoms, treatments, side-effects and prognosis. Below, you will see 2 scenarios. When you click on the right button, the chances of perfect health and death vary, these are examples to help you understand. In the text box below, please enter in the risk of death that you are willing to take in order to avoid this health state.

Health State 10: Watchful waiting, referred to breast clinic for triple assessment - No cancer found

What it means
After a period **watchful waiting** with your GP. Your symptoms were not resolving/became worse. You are referred for **triple assessment** and no cancer was found.

Physical Consequences:
- You have undergone **triple assessment**.

Emotional Consequences:
- Fear of investigations.
- Worry about the delay in diagnosis.
- Fear the results might be wrong and that you still could have cancer.

VAS Scale
Assume that you are currently experiencing the health state you just read. On a scale of 0 to 100 where 0 is death and 100 perfect health where would you rate this health state. Please use the scale below.
When you are happy with your selection please click 'Completed'.

Health State 10 : Watchful waiting, referred to breast clinic for triple assessment - No cancer found

What it means
After a period **watchful waiting** with your GP. Your symptoms were not resolving/became worse. You are referred for **triple assessment** and no cancer was found.

Physical Consequences:
- You have undergone **triple assessment**.

Emotional Consequences:
- Fear of investigations.
- Worry about the delay in diagnosis.
- Fear the results may be wrong and that you still could have cancer.

Standard Gamble.
You have the opportunity to gamble your current health state (as described above) for perfect health, but you also risk death if you take the gamble. It is important to remember about your current health scenario, and your current symptoms, treatments, side-effects and prognosis. Below, you will see 2 buttons. When you click on the right button, the chances of perfect health and death vary, these are examples to help you understand. In the text box below, please enter in the risk of death that you are willing to take in order to avoid this health state.

Health State 11: Watchful waiting, resolution of symptoms, no referral needed.

What it means:
After a period of **watchful waiting**, symptoms have stopped or are resolving within 3 month's follow up with GP. You do not need further investigation.

Physical Consequences:
•.No further tests are needed.

Emotional Consequences:
• Relief that symptoms are not serious.
• Worry that there might be a cancer that has been missed.

VAS Scale
Assume that you are currently experiencing the health state you just read. On a scale of 0 to 100 where 0 is death and 100 perfect health where would you rate this health state. Please use the scale below. When you are happy with your selection please click 'Completed'.

Health State 11: Watchful waiting, resolution of symptoms, no referral needed.

What it means:
After a period of **watchful waiting**, symptoms have stopped or are resolving within 3 month's follow up with GP. You do not need further investigation.

Physical Consequences:
•.No further tests are needed.

Emotional Consequences:
• Relief that symptoms are not serious.
• Worry that there might be a cancer that has been missed.

Standard Gamble.

You have the opportunity to gamble your current health state (as described above) for perfect health, but you also risk death if you take the gamble. It is important to remember about your current health scenario, and your current symptoms, treatments, side-effects and prognosis. Below, you will see 2 buttons. When you click on the right button, the chances of perfect health and death vary, these are examples to help you understand. In the text box below, please enter in the risk of death that you are willing to take in order to avoid this health state.
