# Supplementary material for: Quantifying patient preferences for symptomatic breast clinic referral: a decision analysis study
Source: BMJ Open. 2018 May 31;8(5):e017286. doi: 10.1136/bmjopen-2017-017286 (PMC5988058; doi:10.1136/bmjopen-2017-017286)
Supplement: Author's manuscript [file bmjopen-2017-017286.draft_revisions.pdf]

BMJ Open is committed to open peer review. As part of this commitment we make the peer review history of every article we publish publicly available.

When an article is published we post the peer reviewers' comments and the authors' responses online. We also post the versions of the paper that were used during peer review. These are the versions that the peer review comments apply to.

The versions of the paper that follow are the versions that were submitted during the peer review process. They are not the versions of record or the final published versions. They should not be cited or distributed as the published version of this manuscript.

BMJ Open is an open access journal and the full, final, typeset and author-corrected version of record of the manuscript is available on our site with no access controls, subscription charges or pay-per-view fees (http://bmjopen.bmj.com).

If you have any questions on BMJ Open's open peer review process please email info.bmjopen@bmj.com

BMJ Open

Quantifying patient preferences for symptomatic breast clinic referral: a decision analysis study

Journal:	*BMJ Open*
Manuscript ID	bmjopen-2017-017286
Article Type:	Research
Date Submitted by the Author:	19-Apr-2017

Complete List of Authors:	Quinlan, Aisling; Royal College of Surgeons in Ireland, Department of General Practice<br>O'Brien, Kirsty; Royal College of Surgeons in Ireland, Department of General Practice<br>Galvin, Rose; University of Limerick, 3Department of Clinical Therapies<br>Hardy, Colin; Royal College of Surgeons in Ireland, Department of General Practice<br>McDonnell, Ronan; Royal College of Surgeons in Ireland, Department of General Practice<br>Joyce, Doireann; Royal College of Surgeons in Ireland, Department of General Practice<br>McDowell, Ronald; Royal College of Surgeons in Ireland, HRB Centre for Primary Care Research<br>Ahearn, Emma; Royal College of Surgeons in Ireland, Department of General Practice<br>Keogh, Claire; RCSI, HRB Centre for Primary Care Research<br>O'Sullivan, Katriona; University of Dublin Trinity College<br>Fahey, Tom; Royal College of Surgeons in Ireland, Department of General Practice

**Primary Subject Heading**:	Evidence based practice
Secondary Subject Heading:	Diagnostics, Evidence based practice, Oncology
Keywords:	EPIDEMIOLOGY, ONCOLOGY, Quality in health care < HEALTH SERVICES ADMINISTRATION & MANAGEMENT

SCHOLARONE™
Manuscripts

Quantifying patient preferences for symptomatic breast clinic referral: a decision analysis study

Aisling Quinlan[1], BSc, Kirsty K O'Brien[1], PhD, Rose Galvin[1,3], PhD, Colin Hardy[1], MSc, Ronan McDonnell[1], PhD, Doireann Joyce[2], MB BCh BAO, Ronald D.McDowell[1], PhD, Emma Aherne[1],MB BCh BAO, Claire Keogh[1], PhD, Katriona O'Sullivan[1,4], PhD, Tom Fahey[1], MD

[1]HRB Centre for Primary Care Research, Department of General Practice, Royal College of Surgeons in Ireland (RCSI), 123 St.Stephens Green, Dublin 2, Republic of Ireland.

[2]National University of Ireland Galway, University Road, Galway. Republic of Ireland.

[3]Department of Clinical Therapies, University of Limerick, Limerick.

[4]School of Computer Science and Statistics, Trinity College Dublin.

*Corresponding Author

Tom Fahey, HRB Centre for Primary Care Research, Department of General Practice, Royal College of Surgeons in Ireland, 123 St. Stephen's Green, Dublin 2, Republic of Ireland.

Email: tomfahey@rcsi.ie

Word count:

For peer review only

Abstract

Objectives: To investigate the impact of women's preferences for referral or an alternative strategy of watchful waiting if faced with symptoms that could be due to breast cancer.

Setting: Community-based study.

Participants: Asymptomatic women aged 30-60 years.

Interventions: Participants were presented with 11 health scenarios that represent the possible consequences of symptomatic breast problems. Participants were asked the risk of death that they were willing to take in order to avoid the health scenario using the standard gamble (SG) utility method. This process was repeated for all 11 health scenarios.

Primary outcome measure: The preferred diagnostic strategy, either watchful waiting or referral to a breast clinic. Sensitivity analysis was used to examine how each varied according to changes in the probabilities of the health scenarios.

Results: A total of 35 participants completed the interviews, with median age 41 years (Interquartile range 35 to 47 years). The majority of the study sample were employed (n=32, 91.4%), with a third-level education (n=32, 91.4%) and with knowledge of someone with breast cancer (n=30, 85.7%). When individual preferences were accounted for, 25 (71.4%) patients preferred watchful waiting to referral for triple assessment as their preferred initial diagnostic strategy. Sensitivity analysis shows that referral for triple assessment becomes the dominant strategy at the upper probability estimate (18%) of breast cancer in the community.

Conclusions: Watchful waiting is an acceptable strategy for most women who present to their GP with breast symptoms. These findings suggest that current referral guidelines should take more explicit account of women's preferences in relation in terms of the initial diagnostic strategy for symptomatic breast problems.

For peer review only

Strengths and limitations of this study

Strength of this study is that it accounts for women's preferences in relation to a watchful waiting or immediate referral strategy when faced with symptoms that could be due to breast cancer.

Weakness is that preferences were elicited in asymptomatic women. Women with symptoms may express different preferences.

The impact of eliciting preferences should be done in the context of a randomized trial of a decision aid that uses decision analysis as a form of shared decision making for women with symptoms that could be due to breast cancer.

Taking account of women's preferences might mean that far fewer women would be referred to symptomatic breast clinics with a consequent saving in investigations and costs, and reducing the burden of "medicalization" in women with benign breast disease.

For peer review only

Introduction

Between 2009-2011, it was reported that 31% of cancers in Irish women were due to breast cancer, making it the most prevalent cancer in Irish women [1]. In response, the National Cancer Control Programme (NCCP) clinical guidelines were introduced to streamline the referral process to symptomatic breast units. At present, GPs act as gatekeepers for referral of women with breast symptoms. Referral to symptomatic breast clinics results in triple assessment: a three step process comprising of clinical, radiological and histological examination after fine needle aspiration [2]. Over the past ten years, the number of women referred to symptomatic breast clinics in Ireland has significantly increased, despite the fact there has been no rise in breast cancer incidence [2]. For example in 2006, there were 23,575 new referrals, increasing to 32,249 in 2009 [3],and 37,631 in 2010 [4]. Out of the 37,631 referrals in 2010, only 2,012 were diagnosed with breast cancer [4]. The rise in these referrals for triple assessment without an increase in cancer diagnosis indicates that more women are being exposed to invasive diagnostic investigations.

Clinical prediction rules (CPRs) are clinical tools that quantify the independent impact of factors from a patients history, physical examination and diagnostic tests and stratify patients according to the probability of having a target disorder [5]. Recently, CPRs have been derived and validated to calculate a patient's risk of breast cancer [6, 7], and have been proposed as methods to discriminate between patients at high risk of breast cancer from low risk patients. Such an approach may serve to decrease the number of unnecessary referrals to symptomatic breast clinics in women with a low probability of breast cancer. Patients who are considered low risk could, as an alternative, undergo a watchful waiting strategy (up to 6-8 weeks) with their GP. However, it is unclear from the current literature if patients who are considered low risk would find this acceptable.

Decision analysis is a technique to aid decision making when uncertainty exists over the balance between benefits and risks of treatment [8]. Decision trees have been utilised to assess how patient's preferences impact on diagnosis and treatment for conditions such as ovarian cancer [9] and atrial fibrillation [10]. To date, little is known about how women who have

For peer review only

symptomatic breast problems feel about the options of immediate referral or watching waiting in terms of referral to a symptomatic breast clinic. The aim of this study is assess the impact of patient preferences for immediate referral to a specialist breast clinic or watchful waiting in primary care. We developed a decision tree and elicited patients preferences for these alternative diagnostic strategies by combining individual preferences with probability estimates of breast cancer using decision analysis.

For peer review only

Methodology

Study design

This study utilized decision analysis in the form of utility assessment and folding back a decision tree. The STROBE standardized reporting guidelines were followed to ensure the standardized conduct and reporting of the research. Ethical approval was received from the Research Ethics Committee at the Royal College of Surgeons in Ireland.

Developing the decision tree and health scenarios

Literature searches were conducted in medical databases such as PubMed, the Cochrane library and Web of Science to assist with the development of the decision tree and health scenarios. A specialist registrar with clinical experience in a symptomatic breast clinic was also consulted during this development phase. Following these consultations, the decision tree was constructed to portray the temporal course of the two diagnostic options (Figure 1). The decision tree had one decision node with two diagnostic branches (a choice between watchful waiting or immediate referral), nine probability or chance nodes and eleven health outcome nodes. In accordance with the literature the tree was designed such that only two branches emanated from each chance node [11]. The number of outcome nodes differed between the two branches of the tree in order to accommodate the additional step (an observation period before breast cancer assessment among patients who return to their GP) in the watchful waiting branch of the tree. It is not always possible or appropriate for each branch of a decision tree to have the same number of outcome nodes as this will be dependent on the nature and

temporal order of events in that branch [12]. The decision tree is “balanced” as each branch has advantages and disadvantages and hence it is not possible to readily identify a treatment path that is ideal in all respects [11].

A total of eleven health scenarios were constructed, one for each outcome node. For this study the standard gamble (SG) method of utility assessment was used [13] [14]. Briefly, participants presented with a description of each health state, its physical consequences and emotional consequences, and were given the opportunity to remain in the health state or to gamble it for perfect health, but with the risk of death if they took the gamble. Participants were presented with differing probabilities showing how the chance of perfect health and death varied with each other for each health state (the “ping-pong method”). The utility value was one minus the risk of death they were willing to take in order to avoid the health state described. The 11 health states were presented in a random order.

Probability estimates for each chance node were obtained from the medical literature [7,15-28]. Where possible, relevant probability estimates for each chance node were combined using random effects meta-analysis (Table 1). The same probabilities were used for each participant. It was not possible to estimate from the literature the probability of a diagnosis of breast cancer among patients who returned to their doctor following a period of watchful waiting. Clinical experience suggested this would be several percentage points higher in absolute terms than the probability of a diagnosis of breast cancer following RTA and an estimate of 10% was used.

Participants and setting

Interviews were conducted over a six month period in Dublin from October 2014 to April 2015. Asymptomatic women aged 30-60 years were invited to participate in the study. Participants were recruited using staff notice boards, social media and snowball sampling. The majority of interviews were carried out in a meeting room in the research building. Some interviews were conducted in peoples’ place of work as this was more convenient for them. Written informed

For peer review only

consent was obtained from all participants prior to participation. The interviews were conducted with a researcher present, who was available to answer any questions and to obtain feedback about the interview. Prior to the interview, participants were given a baseline demographics questionnaire and a multiple-choice questionnaire to assess their knowledge of breast cancer.

Utility assessment

A computer-based assessment programme which presented and recorded participant responses was designed. This programme incorporated an introduction, an explanation of the interview process, and presentation of each clinical scenario in random order using the standard gamble method of utility assessment. An example of the standard gamble was included for orientation and definitions were provided for any medical terminology used. The purpose of using a computer programme was to allow the interviewer to limit their interaction with each participant, standardize utility assessment and minimize interviewer bias. A pilot study was carried out and modifications made to the computer programme and utility assessment process.

Ethical approval and data Protection

The data from the computer programme was stored with a participant ID only and saved to an encrypted password protected desktop computer. To protect patient identity, a MS excel spreadsheet was created; containing just patient names and identification numbers, and this was stored in a secured, password protected folder on the server and kept separate to the data from the interview. The study was approved by the RCSI institutional ethics committee.

Decision analysis

The utility for each outcome, "a number assigned to the quality of life a patient would attach to a particular outcome on a defined scale " [12], was calculated as 1-the risk of death (expressed as a fraction) a participant was willing to take in order to avoid the health scenario as described. For each participant, an expected utility score for each diagnostic option was obtained by

For peer review only - http://bmjopen.bmj.com/site/about/guidelines.xhtml

multiplying and summing the relevant probabilities and utilities. The branch which had the highest expected utility score was determined to be the preferred diagnostic strategy- watchful waiting or referral for triple assessment.

Sensitivity analysis was used to determine the robustness of the conclusion drawn from the decision tree [29]. For our study each of the probability estimates derived from the literature was allowed to vary between the lower and upper limits of the 95% confidence interval for that estimate, in order to assess the extent to which the preferred diagnostic strategy changed. The difference between the number of patients preferring watchful waiting using the upper limit of the 95% confidence interval and the number of patients preferring watchful waiting using the lower limit of the 95% confidence interval for each probability estimate (the swing) was squared and totalled; the impact of varying each of the probability estimates was measured as a percentage of the total squared-swing. The sensitivity analyses accounted for the fact that some probability estimates were used more than once by incorporating the conditioning event into the definition of the variables under consideration [30]. An additional two-way sensitivity analysis explored how women's preferred diagnostic option changed as the probability of diagnosis following watchful waiting varied relative to the probability of diagnosis following referral and triple assessment.

Standard descriptive statistics were used to summarise the sample. Results were held to be significant if they refer to statistical significance on a two-sided test evaluated at the 5% level. All analyses were performed using STATA 13 [31].

For peer review only

Results

A total of 36 women participated, with 35 interviews completed in full. The median age of participants was 41 years (interquartile range (IQR) 35 to 47 years). The demographic details of participants are described in Table 2. The majority of participants were employed (32, 91.4%), had a third-level education (32, 91.4%) and knew someone with breast cancer (30, 85.7%). Less than half the study sample (15, 42.9%) reported having a prior mammography. There was borderline evidence of a negative correlation between participants' self-reported knowledge of breast cancer and their actual knowledge based on responses to a multiple-choice questionnaire (p=0.06).

When patient preferences were taken into account, 25 patients (71.4%, 95%CI 19%, 30%) preferred watchful waiting to referral as the initial diagnostic strategy. Sensitivity analysis showed that the overall preferred diagnostic strategy changed to referral when the probability of breast cancer following a period of watchful waiting increased; only ten women favoured watchful waiting when the probability of breast cancer following a period of watchful waiting had increased to 18% (Table 3). Swings in the group preference according to changes in the probabilities associated with each of the stage of breast cancer were small in comparison (Table 3).

In terms of two-way sensitivity analysis more than half the participating women still preferred watchful waiting when the probability of a diagnosis of breast cancer following a period of watchful waiting was 2.25 times as high as the probability of diagnosis following referral and triple assessment. (Figure 2) This pattern remained consistent irrespective of the probability of breast cancer following referral and triple assessment, estimated at levels of 4%, 6%, 8% and 10%.

For peer review only - http://bmjopen.bmj.com/site/about/guidelines.xhtml

Discussion

Summary of main findings

This study shows that a strategy of watching waiting in women with breast symptoms in primary care should be considered as an acceptable alternative to immediate referral to a symptomatic breast clinic. These findings suggest that women's own preferences should form an important element of the decision to refer to a symptomatic breast clinic and that these preferences should be accounted as part of a shared decision making process. Sensitivity analysis shows that as the probability of breast cancer increases, the diagnostic strategy of referral for triple assessment dominates. Two way sensitivity analysis shows that, across the likely probability range of breast cancer under a watchful waiting strategy, referral for triple assessment becomes the dominant strategy once the probability of breast cancer is two and a half times more likely than under a watchful waiting strategy.

Results in the context of the current literature

Although utility assessment has been used to evaluate the acceptability of various clinical states and treatment regimes in the breast cancer literature [32-34] it has not been used to examine the acceptability of watchful waiting in low risk patients presenting with signs and symptoms of possible breast cancer. A previous study by McCafferty et al. found that watchful waiting is acceptable to patients with low grade ductal carcinoma in-situ (DCIS) but this depended on the terminology used for the disease [35]. These findings show that women are prepared to accept a conservative, non-invasive strategy when the probability of breast cancer is low.

Watchful waiting has also been described as a key technique for more effective management of other cancers such as prostate cancer [36]. In prostate cancer the use of PSA testing has led to an increase in the incidence of prostate cancer but not a decrease in the mortality rate, suggesting that PSA testing often identifies low risk cancers [37]. Given the serious and common side effects associated with treatment in prostate cancer some studies have shown that some men prefer watchful waiting to treatment options [38]. In our study the side effects of further diagnostic testing (triple assessment) are less onerous than treatment of localized prostate cancer, but many women would experience high levels of anxiety and stress upon

For peer review only

referral to a symptomatic breast clinic. This study suggests that many women would find it more acceptable to undergo watchful waiting for a period rather than participate in diagnostic testing when their risk of breast cancer is low.

A qualitative study examining women's views on over-diagnosis in breast cancer screening showed that 50% of the population study felt they needed to make more careful personal decisions about screening and would consider watchful waiting as an alternative route if they were found to have a low risk cancer [39]. Like our study watchful waiting was found to be an acceptable strategy when the risk involved is low and the health outcomes are positive.

Strengths and limitations

This study has several strengths. We used an evidence-based approach, quantifying probabilities from the literature, and developing health states that incorporate the common features of breast cancer. We tested and modified the wording of the health states and the computer programme in a pilot group of participants and refined our protocol based on feedback from these participants. We tried to limit any bias caused by the interviewer by using a computer programme and providing the participant with written definitions and information. Examples of the standard gamble were given to the participants prior to the interview, so participants had the opportunity to familiarize themselves with the method. Although the sample was relatively small, it included participants with a range of ages and knowledge of breast cancer.

Clinical and policy Implications

The current referral process leads to many patients who are at low risk of breast cancer being referred to busy symptomatic breast clinics, with a risk of iatrogenic harm, increased patient anxiety and over-medicalizing of a self-limiting condition. With more emphasis on the development of clinical prediction rules, it will be possible to identify and quantify each individual women's risk of breast cancer. A recent study derived a clinical prediction rule (CPR) for predicting the risk of breast cancer for women presenting to their GP with breast symptoms.

For peer review only

[7] A total of 6,590 patients were included in the derivation study and 4.9% were diagnosed with breast cancer. Independent clinical predictors for breast cancer were: increasing age by year (adjusted OR 1.08, 95%CI 1.07-1.09); presence of a lump (5.63, 95%CI 4.2-7.56); nipple change (2.77, 95%CI 1.68-4.58) and nipple discharge (2.09, 95% CI 1.1-3.97). Validation of the rule (n=911) also shows that the probability of breast cancer is higher with an increasing number of these independent clinical variable. By incorporating patient preferences with an individual's probability of breast cancer from a CPR, a strategy of watchful waiting is acceptable, cost effective and likely to avoid considerable iatrogenic harm. Clinical practice guidelines should incorporate probability estimates of breast cancer based on CPRs and urge elicitation of patient preference for alternative diagnostic options- watchful waiting or referral. More formally, decision aids that could be developed in conjunction with the symptomatic breast CPRs to enable the alternative options of watchful waiting or referral based on individual preferences.

Conclusion

Watchful waiting appears to be an acceptable diagnostic strategy for women who present to their general practitioner with breast symptoms. These findings should be validated in a larger and more comprehensive study of patient preferences in women with breast symptoms. Clinical practice guidelines should reflect the importance of eliciting women's preferences for watchful waiting as an alternative to referral to a symptomatic breast clinic, particularly in women with low risk clinical features.

Competing interests

The authors declare that they have no competing interests.

For peer review only

Authors' contributions

All authors were involved in the study conception and design. AQ, RG and KOB acquired data for analysis. RmcD designed the computer-based utility analysis programme. CH and RDMcD performed statistical analysis. AQ interpreted the data and drafted the paper. KOB, RG, RDMcD and TF critically revised the draft manuscript. All authors read and approved the final manuscript.

Funding statement

Health Research Board of Ireland grant number HRC-2007-1.

Data sharing statement

We are happy to share the data from this study

For peer review only

References

1. National Cancer Registry: **Cancer in Ireland 1994-2011: Annual report of the National Cancer Registry 2014**. In. Cork: National Cancer Registry; 2014.
2. O'Rourke N: **Review of Referral Patterns and Triage Processes in Symptomatic Breast Units - a Hospital Perspective**. In.: HSE; 2012: 72.
3. National Cancer Control Programme: **Review of activity in the symptomatic breast clinics in the eight designated centres, 2005 and 2006 compared with 2009 year to date.** . In.; 2009.
4. National Cancer Control: **Report on the Symptomatic Breast Disease Key Performance Indicators 2010.** In.; 2011.
5. Keogh C, Wallace E, O'Brien KK, Galvin R, Smith SM, Lewis C, Cummins A, Cousins G, Dimitrov BD, Fahey T: **Developing an international register of clinical prediction rules for use in primary care: a descriptive analysis**. *Annals of family medicine* 2014, **12**(4):359-366.
6. McCowan C, Donnan PT, Dewar J, Thompson A, Fahey T: **Identifying suspected breast cancer: development and validation of a clinical prediction rule**. *The British journal of general practice : the journal of the Royal College of General Practitioners* 2011, **61**(586):e205-214.
7. Galvin R, Joyce D, Downey E, Boland F, Fahey T, Hill AK: **Development and validation of a clinical prediction rule to identify suspected breast cancer: a prospective cohort study**. *BMC cancer* 2014, **14**(1):743.
8. Detsky AS NG, Krahn MD, Naimark D, Redelmeier DA.: **Primer on medical desicion analysis: Part 1 - getting started.** *Medical Decision Making* 1997, **17**(125).
9. Havrilesky LJ BG, Davis DM, Nolte KC, Barnett JC, Myers ER et al. : **Determination of quality of life-related utilities for health states relevant of ovarian cancer diagnosis and treatment.** *Gynecologic Oncology* 2009, **113**:216-220.
10. Protheroe J FT, Montgomery AA, Peters TJ. : **The impact of patients' preferences on the treatment of artial fibrilation: observational study of patient based decision analysis.** *The British Medical Journal* 2000.
11. Allan S, Detsky MD, Naglie G, Krahn MD, Redelmeier DA, Naimark D. **Primer on Medical Decision Analysis: Part 2-Building a Tree**. *Medical Decision Making* 1997. 17(2):126-35.
12. Lee A, Goynt GM, Ho AMH, Keitz S, McGinn T, Wyer P, EBM Teaching Scripts Working Group. **Tips for Teachers of Evidence-based Medicine: Making Sense of Decision Analysis Using a Decision Tree**. *Journal of General Internal Medicine* 2009. 24(5): 642–648
13. von Neumann H, Morgenstern O. **Theory of games and economic behaviour**. 1944. Princeton, NJ: Princeton University Press.
14. Drummond M SM, Torrance GW, O' Brien BJ, Stoddart GL: **Methods for the Economic Evaluation of Health Care Programmes.** Oxford New York: Oxford University Press; 2005.
15. National Cancer Registry: Breast Cancer Incidence, Mortality, Treatment and Survival in Ireland: 1994-2009. National Cancer Registry, Cork, Ireland 2012.
16. Davidson A, Chia S, Olson R, Nichol A, Speers C, Coldman AJ, Bajdik C, Woods R, Tyldesley S: **Stage, treatment and outcomes for patients with breast cancer in British Columbia in 2002: a population-based cohort study**. *CMAJ open* 2013, **1**(4):E134-141.
17. Louwman WJ, Voogd AC, van Dijck JA, Nieuwenhuijzen GA, Ribot J, Pruijt JF, Coebergh JW: **On the rising trends of incidence and prognosis for breast cancer patients diagnosed 1975-2004: a long-term population-based study in southeastern Netherlands**. *Cancer causes & control : CCC* 2008, **19**(1):97-106.

For peer review only

18. Keegan THM, Press DJ, Tao L, DeRouen MC, Kurian AW, Clarke CA, Gomez SL: **Impact of breast cancer subtypes on3-year survival among adolescent and young adult women**. *Breast Cancer Research* 2013: 15(5):R95.

19. Morris CR, Ramirez CN, Cook SN, Parikh-Patel A, Kizer KW, Bates JH, Snipes KP. **Cancer Stage at Diagnosis**, 2013. Sacramento, CA: California Department of Public Health, California Cancer Registry.

20. Wang Q., Li J, Zheng S, Li J-L, Pang Y, Huang R, Zhang B-N, Zhang B, Yang H-J, Xie X-M, Tang Z-H, Li H, He J-J, Fan J-H, Qiao Y-L. **Breast cancer stage at diagnosis and area-based socioeconomic status: a multicenter 10-year retrospective clinical epidemiological study in China**. *BMC Cancer* 2012. 12:122.

21. Walters S, Maringe C, Butler J, Rachet B, Barrett-Lee P , Bergh J, Boyages J , Christiansen P , Lee M, Wärnberg F, Allemani C , Engholm G , Fornander T, Gjerstorff ML, Johannesen TB, Lawrence G, McGahan CE, Middleton R, Steward J, Tracey E, Turner D, Richards MA, Coleman MP, The ICBP Module 1 Working Group. **Breast cancer survival and stage at diagnosis in Australia, Canada, Denmark, Norway, Sweden and the UK, 2000-2007: a population-based study**. *British Journal of Cancer* 2013. 108, 1195–1208.

22. Campbell C, Durning P, Cheema I, Naisby G. **A simple tool for rapid access to a symptomatic breast clinic**. *European Journal of Surgical Oncology* 2004**.** 30(3):248–251.

23. McCowan C, Donnan PT, Dewar J, Thompson A, Fahey T. **Identifying suspected breast cancer: development and validation of a clinical prediction rule**. *British Journal of General Practice* 2011. 61(585): e205-e214.

24. Cochrane RA, Singhal H, Monypenny IJ, Webster DJ, Lyons K, Mansel RE: **Evaluation of general practitioner referrals to a specialist breast clinic according to the UK national guidelines**. *European journal of surgical oncology : the journal of the European Society of Surgical Oncology and the British Association of Surgical Oncology* 1997, **23**(3):198-201.

25. Nichols S, Waters WE, Wheeler MJ. **Management of female breast disease by Southampton general practioners**. *British Medical Journal* 1980. 281: 1450-1453.

26. Dohrmann PJ, Hugher ESR, McDermott, Price A. **Sympton Duration, tumor staging and survival in patients with carcinoma of the breast**. *Surgery, Gynecology & Obstetrics* 1982. 154: 707-710.

27. Charlson ME. **Delay in the treatment of carcinoma of the breast.** *Surgery, Gynecology & Obstetrics* 1985. 160:393-399.

28. Givio (Interdisciplinary Group for Cancer Care Evaluation) Italy. **Reducing Diagnostic Delay in Breast Cancer: Possible Therapeutic Implications**. *Cancer* 1986. 58:1756-1761

29. Murray DK, Naglie G, Naimark D, Redelmeier DA, Detsky AS. **Primer on Medical Decision Analysis: Part 4-Analyzing the Model and Interpreting the Results**. *Medical Decision Making* 1997. 17(2):142-51.

30. Sendi PP, Clemen RT: **Sensitivity analysis on a chance node with more than two branches**. *Medical decision making : an international journal of the Society for Medical Decision Making* 1999, **19**(4):499-502.

31. StataCorp LP: **STATA User's Guide Release 13**. In. College Station, Texas; 2013.

32. Buyukdamgaci-Alogan G, Elele T, Hayran M, Erman M, Kilickap S: **A decision-analytic model for early stage breast cancer: lumpectomy vs mastectomy**. *Neoplasma* 2008, **55**(3):222-228.

33. Chie WC, Huang CS, Chen JH, Chang KJ: **Utility assessment for different clinical phases of breast cancer in Taiwan**. *Journal of the Formosan Medical Association = Taiwan yi zhi* 2000, **99**(9):677-683.

34. Sinno H, Izadpanah A, Vorstenbosch J, Dionisopoulos T, Ibrahim AM, Tobias AM, Lee BT, Lin SJ: **Living with a unilateral mastectomy defect: a utility assessment and outcomes study**. *Journal of reconstructive microsurgery* 2014, **30**(5):313-318.

For peer review only

35. McCaffery K, Nickel B, Moynihan R, Hersch J, Teixeira-Pinto A, Irwig L, Barratt A: **How different terminology for ductal carcinoma in situ impacts women's concern and treatment preferences: a randomised comparison within a national community survey**. *BMJ open* 2015, **5**(11):e008094.
36. Mukai TO, Bro F, Olesen F, Vedsted P: **To test or not: a registry-based observational study of an online decision support for prostate-specific antigen tests**. *International journal of medical informatics* 2013, **82**(10):973-979.
37. Esserman L, Shieh Y, Thompson I: **Rethinking screening for breast cancer and prostate cancer**. *Jama* 2009, **302**(15):1685-1692.
38. Hall IJ, Lee Smith J: **Evolution of a CDC Public Health Research Agenda for Low-Risk Prostate Cancer**. *American journal of preventive medicine* 2015, **49**(6s5):S483-s488.
39. Hersch J, Jansen J, Barratt A, Irwig L, Houssami N, Howard K, Dhillon H, McCaffery K: **Women's views on overdiagnosis in breast cancer screening: a qualitative study**. *BMJ (Clinical research ed)* 2013, **346**:f158.

For peer review only

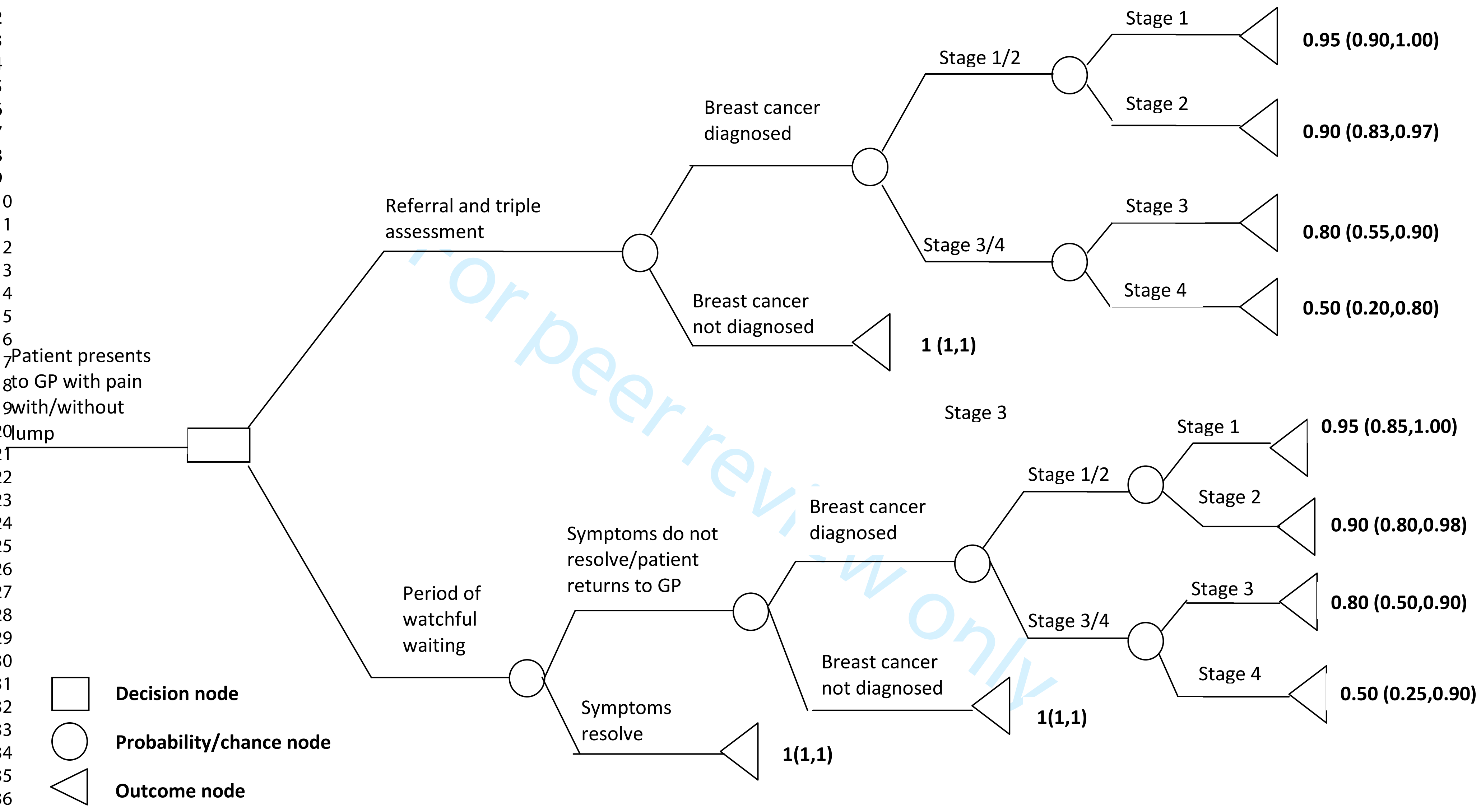

Figure 1. Decision tree for health states in the treatment of breast cancer with utility values (median (interquartile range)) for each state

Table 1 Probability estimates used in the breast cancer decision tree analysis

Treatment path	Outcome	Estimated probability (95% CI)	References
Referral & Triple Assessment (RTA)	Breast cancer diagnosed subsequent to RTA	0.06 (0.05,0.08)	7,22,23,24
	Stage 1 breast cancer among patients diagnosed with breast cancer following RTA	0.34 (0.26,0.42)	15,16,17,18,19,20,21
	Stage 2 breast cancer among patients diagnosed with breast cancer following RTA	0.46 (0.39, 0.52)	15,16,17,18,19,20,21
	Stage 3 breast cancer among patients diagnosed with breast cancer following RTA	0.15 (0.13,0.17)	15,16,17,18,19,20,21
	Stage 4 breast cancer among patients diagnosed with breast cancer following RTA	0.05 (0.04,0.06)	15,16,17,18,19,20,21
Watchful waiting (WW)	Symptoms do not resolve/worsen and patient returns to GP following period of WW	0.34 (0.28,0.41)	25
	Breast cancer diagnosed among patients who return to GP following WW	0.10 (0.05,0.18)	Clinical estimate
	Stage 1 breast cancer among patients diagnosed with breast cancer following WW	0.27 (0.20,0.33)	26,27,28
	Stage 2 breast cancer among patients diagnosed with breast cancer following WW	0.42 (0.36,0.47)	26,27,28
	Stage 3 breast cancer among patients diagnosed with breast cancer following WW	0.20 (0.16,0.22)	26,27,28
	Stage 4 breast cancer among patients diagnosed with breast cancer following WW	0.11 (0.09,0.13)	26,27,28

For peer review only

Table 2. Descriptive statistics for the study sample (n=35 participants)

**Variable**		**Median (Interquartile range)**
Age (years)		41 (35 to 47)
Self-rated knowledge of breast cancer (maximum score 10)		5 (4 to 7)
Actual knowledge of breast cancer (maximum score 7)		3 (2 to 4)
		**Number of participants (%)**
Location	Dublin & surrounds	23 (65.7%)
	West of Ireland	10 (28.6%)
	Overseas	2 (5.7%)
Education	Secondary	3 (8.6%)
	Third level	32 (91.4%)
Employed	Yes	32 (91.4%)
	No	3 (8.6%)
Family carer	Yes	18 (51.4%)
	No	17 (49.6%)
Know someone with breast cancer	Yes	30 (85.7%)
	No	5 (14.3%)
Have had a mammography	Yes	15 (42.9%)
	No	20 (57.1%)

For peer review only

Table 3. Sensitivity analysis for breast cancer patient treatment preference study

Health scenario	Values for sensitivity analysis			Number of patients preferring WW to RTA			Swing	$Swing^2$	$\%Swing^2$
	Lower limit for probability	Reference probability	Upper limit for probability	Lower limit for probability	Reference probability	Upper limit for probability			
Diagnosis of breast cancer following RTA	0.05	0.06	0.08	23	25	27	4	16	3.21
Stage 1 breast cancer among patients diagnosed following RTA	0.26	0.34	0.42	26	25	21	-5	25	5.02
Stage 2 breast cancer among patients diagnosed following RTA	0.39	0.46	0.52	26	25	24	-2	4	0.80
Stage 3 breast cancer among patients diagnosed following RTA	0.13	0.15	0.17	25	25	25	0	0	0.00
Stage 4 breast cancer among patients diagnosed following RTA	0.04	0.05	0.06	25	25	25	0	0	0.00
Symptoms do not resolve/worsen and patient returns to GP following period of WW	0.28	0.34	0.41	27	25	24	-3	9	1.81
Diagnosis of breast cancer among patients returning to GP after period of watchful waiting	0.05	0.10	0.18	31	25	10	-21	441	88.55
Stage 1 breast cancer among patients diagnosed following WW	0.20	0.26	0.33	25	25	26	1	1	0.20
Stage 2 breast cancer among patients diagnosed following WW	0.36	0.41	0.47	25	25	26	1	1	0.20
Stage 3 breast cancer among patients diagnosed following WW	0.16	0.19	0.22	25	25	25	0	0	0
Stage 4 breast cancer among patients diagnosed following WW	0.09	0.11	0.13	26	25	25	1	-1	0.20

RTA: Referral and Triple Assessment ; WW: Watchful Waiting

Swing=Number of patients preferring WW to RTA (upper limit for probability)- number of patients preferring WW to RTA (lower limit for probability)

$Swing^2$=Swing*Swing

%Swing= $Swing^2$ x100 /(Total $Swing^2$ (=498))

For peer review only

Figure 2 Two way sensitivity analysis according to probability under referral and triple asesssment (four levels) at incremental probability levels under watchful waiting

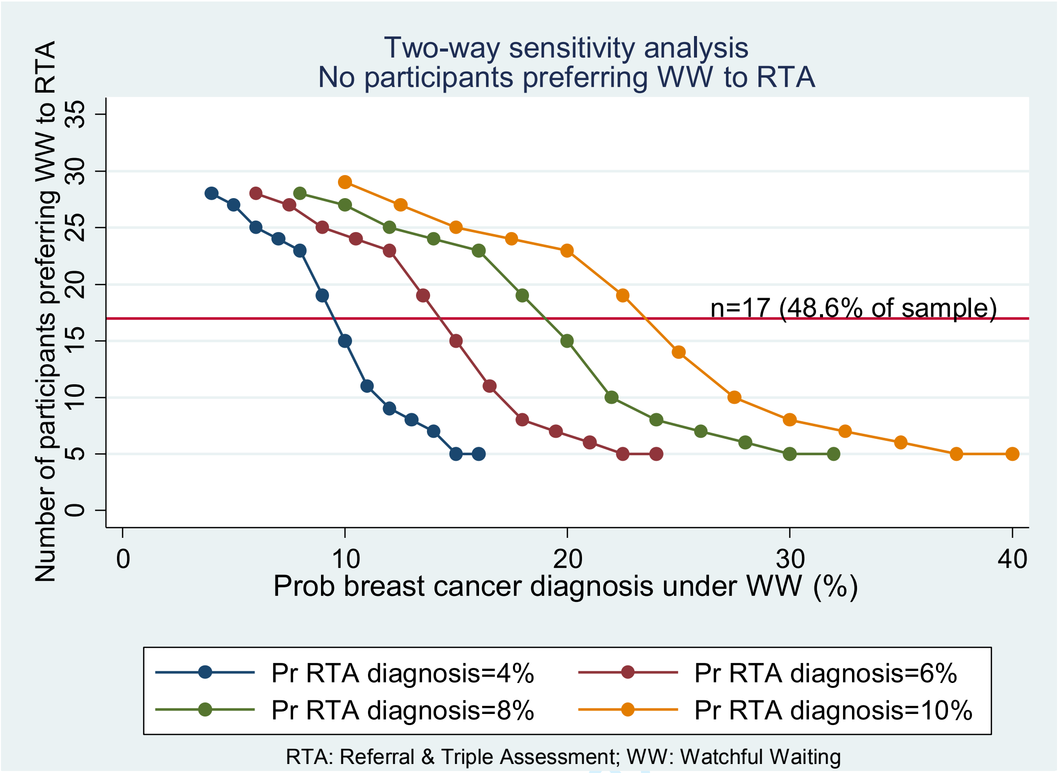

5/5/2015

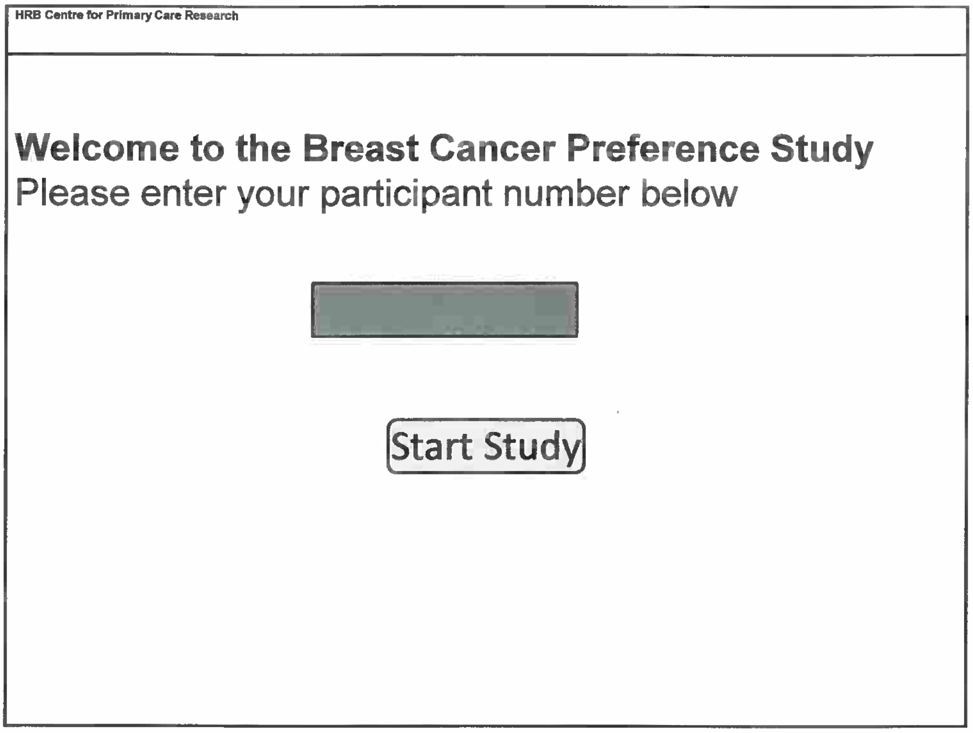
HRB Centre for Primary Care Research
Welcome to the Breast Cancer Preference Study
Please enter your participant number below
Start Study

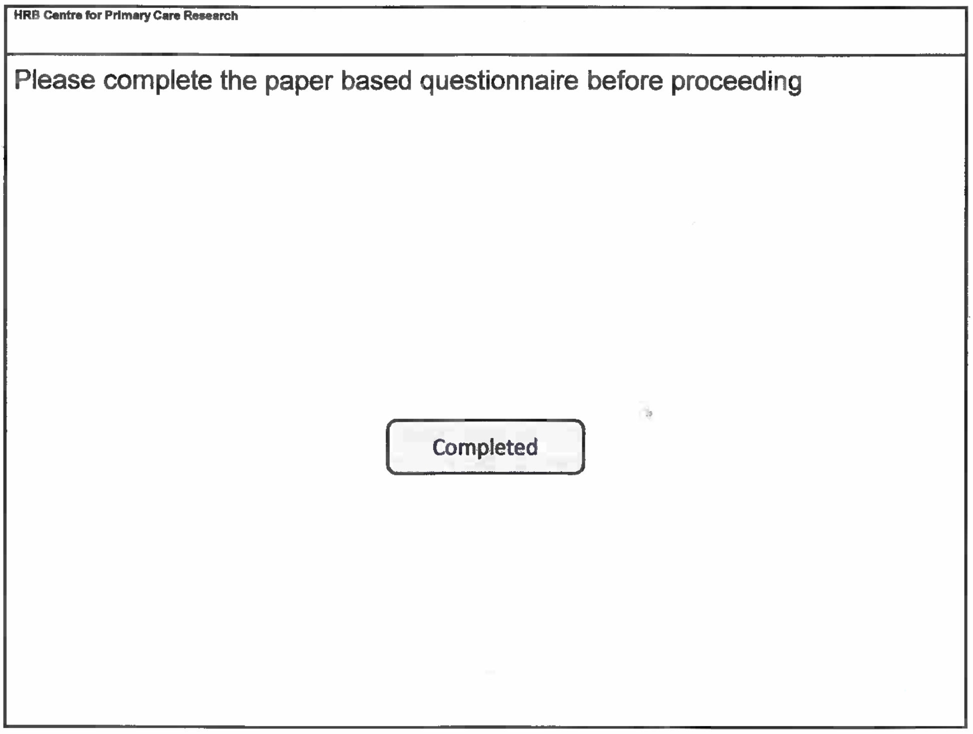
HRB Centre for Primary Care Research
Please complete the paper based questionnaire before proceeding
Completed

5/5/2015

HRB Centre for Primary Care Research

This is a study aimed at understanding womens' perceptions of breast cancer. Women with certain risk factors, signs and symptoms are more likely than others to develop breast cancer.

We will present you with several health scenarios, and ask you to choose between different states of health.

Next

Scenario

You have presented to your GP with symptom(s) of breast cancer.
Your GP will take a history of your symptoms, and perform a breast examination. They may also perform any other examinations/tests that they feel are necessary (listen to your heart, take blood, etc.).
Based on history and examination findings, your GP will either recommend a "watchful waiting" programme, or triple assessment at a specialist breast clinic. However, it is your decision which option you prefer.

Definitions

Beside you are definitions for terms that you will come across in the scenarios. Please read them now, but you will also have a copy of them beside you to refer to at any point. Any word in **blue** in the scenarios has a definition provided.

How will we measure your preferences?

We will use 2 methods (the VAS and standard gamble) to assess how you feel about each of the 11 health states. The health states will be presented to you in random order.

An explanation of each of the methods will follow. You also have laminated cards in front of you with the same information.

You can refer to these at any time that you wish.

Visual Analogue Scale - VAS

The Visual Analogue Scale is a scale from 0-100, with 0 being the worst health scenario you can think of (death), and 100 being the best health scenario (perfect health).

We will ask you to read each health care scenario individually, and then to place it where you think it fits along the scale.

You do not need even gaps between scenarios on the scale, and you can place two or more scenarios on the same number if you wish.

Scale: 0-100

Please include VAS scale here

36 [illegible]
Scen 1-5
Type Asse.
6-11
Watch Wil

Standard Gamble.

You will be presented with 11 different health state scenarios, each scenario represents your current permanent health state. We will ask you to make a gamble between your current health state with two other health states (perfect health or death). I want you to imagine that this carries a certain chance of you living the rest of your life in perfect health and a certain chance of you dying painlessly tomorrow.

It is important to remember your current health scenario, and your current symptoms, treatments, side-effects and prognosis. You will be given different chances of perfect health versus death, against the current scenario, and keep gambling until you reach a point where you cannot choose between the 2 options.

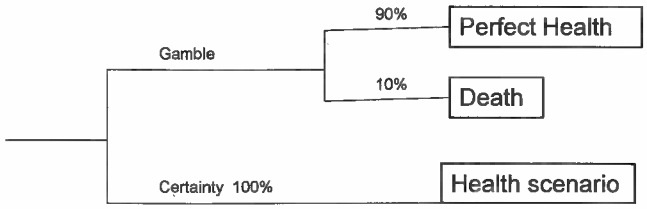

5/5/2015

Example- Standard Gamble

Imagine you have a heart condition. You have a choice between taking medication which has unpleasant side effects or taking no medication.

If you had to choose between taking this medication with side-effects for the rest of your life, or taking no medication and have perfect health, but with the risk of dying, what risk of death would you take to avoid the medication and side effects?

Another way of putting it is - what risk of dying painlessly tomorrow would you be willing to accept (if you didn't take the drugs) before you change your mind and decide you would rather take them?

•If offered a 90% chance of perfect health, no drugs, no side effects, but with a 10% risk of instant death, would you take it?

•If offered a 70% chance of perfect health, no drugs, no side effects, but with a 30% risk of instant death, would you take it?

Below, you will see 2 buttons. When you click on the right button, the chances of perfect health and death vary, these are examples to help you understand. In the text box below, please enter in the risk of death that you are willing to take.

no drugs, no side effects

Live in current Health State.	Versus	50% chance of perfect health and 50% chance of death.

instant

[ ] % chance of death

Enter

The Study

We are going to present you with 11 possible health scenarios in a random order - please follow the instructions and if you have any questions please ask.

Definitions have been provided for any word or phrase in **blue**.

5/5/2015

1.0069

Health State 1: Referred to breast clinic for triple assessment - Stage 1 Breast Cancer

What it means:
You were referred for **triple assessment** and breast cancer has been diagnosed that is confined to the breast and may involve a small number of **lymph nodes**. This is an early stage of breast cancer.

Physical Consequences:
- You have undergone **triple assessment.**
- You will have surgery (**lumpectomy, mastectomy, lymph node removal**).
- **Chemotherapy** and/or **radiation therapy** to prevent the cancer from coming back.
- **Hormonal therapy** or **biological therapy** may be given, depending on the type of cancer cell.

Emotional Consequences:
- Fear over investigations and treatments (pain, side-effects).
- Anxiety or depression associated with diagnosis.
- Worry that cancer may spread or will not be fully cured.

VAS Scale
Assume that you are currently experiencing the health state you just read. On a scale of 0 to 100 where 0 is death and 100 perfect health where would you rate this health state. Please use the scale below.
When you are happy with your selection please click 'Completed'.

Health State 1: Referred to breast clinic for triple assessment - Stage 1 Breast Cancer

What it means:
You were referred for **triple assessment** and breast cancer has been diagnosed that is confined to the breast and may involve a small number of **lymph nodes**. This is an early stage of breast cancer.

Physical Consequences:
- You have undergone **triple assessment.**
- You will have surgery (**lumpectomy, mastectomy, lymph node removal**).
- **Chemotherapy** and/or **radiation therapy** to prevent the cancer from coming back.
- **Hormonal therapy** or **biological therapy** may be given, depending on the type of cancer cell.

Emotional Consequences:
- Fear over investigations and treatments (pain, side-effects).
- Anxiety or depression associated with diagnosis.
- Worry that cancer may spread or will not be fully cured.

Standard Gamble
You have the opportunity to gamble your current health state (as described above) for perfect health, but you also risk death if you take the gamble. It is important to remember about your current health scenario, and your current symptoms, treatments, side-effects and prognosis. Below, you will see 2 buttons. When you click on the right button, the chances of perfect health and death vary, these are examples to help you understand. In the text box below, please enter in the risk of death that you are willing to take in order to avoid this health state.

\ type

5/5/2015

Health State 2: Referred to breast clinic for triple assessment - Stage 2 Breast Cancer

What it means:
Triple assessment has found cancer that is contained within the breast. It may also be in surrounding **lymph nodes**.

Physical Consequences:
• You have undergone **triple assessment.**
• You will have surgery **(lumpectomy, mastectomy, lymph node removal)**.
• You will have **radiation therapy.**
• You may have **chemotherapy** and/or **hormonal therapy,** depending on the type of cancer.

Emotional Consequences:
• Fear over investigations and treatments (pain, side-effects).
• Anxiety or depression associated with diagnosis.
• Fear of recurrence.

<u>VAS Scale</u>
Assume that you are currently experiencing the health state you just read. On a scale of 0 to 100 where 0 is death and 100 perfect health where would you rate this health state. Please use the scale below. When you are happy with your selection please click 'Completed'.

Health State 2: Referred to breast clinic for triple assessment; Stage 2 Breast Cancer

What it means:
Triple assessment has found cancer that is contained within the breast. It may also be in surrounding **lymph nodes**.

Physical Consequences:
• You have undergone **triple assessment.**
• You will have surgery **(lumpectomy, mastectomy, lymph node removal)**.
• You will have **radiation therapy.**
• You may have **chemotherapy** and/or **hormonal therapy,** depending on the type of cancer.

Emotional Consequences:
• Fear over investigations and treatments (pain, side-effects).
• Anxiety or depression associated with diagnosis.
• Fear of recurrence.

<u>Standard Gamble</u>
You have the opportunity to gamble your current health state (as described above) for perfect health, but you also risk death if you take the gamble. It is important to remember about your current health scenario, and your current symptoms, treatments, side-effects and prognosis. Below, you will see 2 buttons. When you click on the right button, the chances of perfect health and death vary, these are examples to help you understand. In the text box below, please enter in the risk of death that you are willing to take in order to avoid this health state.

5/5/2015

Health State 3: Referred to breast clinic for triple assessment - Stage 3 Cancer

What it means:
You were referred for **triple assessment** and cancer was found in the breast, it has spread into surrounding tissues and many **lymph nodes** are involved.

Physical consequences:
- You have undergone **triple assessment.**
- You will have a surgery (**lumpectomy, mastectomy, lymph node removal**).
- You will probably have **radiation therapy** and/or **chemotherapy.**
- You may have **hormonal** or **biological therapy**, depending on the type of cancer cells.

Emotional consequences:
- Fear over investigations and treatments (pain, side-effects).
- Poorer prognosis, worry about survival.
- Fear of recurrence.

VAS Scale
Assume that you are currently experiencing the health state you just read. On a scale of 0 to 100 where 0 is death and 100 perfect health where would you rate this health state. Please use the scale below. When you are happy with your selection please click 'Completed'.

Health State 3: Referred to breast clinic for triple assessment - Stage 3 Cancer

What it means:
You were referred for **triple assessment** and cancer was found in the breast, it has spread into surrounding tissues and many **lymph nodes** are involved.

Physical consequences:
- You have undergone **triple assessment.**
- You will have a surgery (**lumpectomy, mastectomy, lymph node removal**).
- You will probably have **radiation therapy** and/or **chemotherapy.**
- You may have **hormonal** or **biological therapy**, depending on the type of cancer cells.

Emotional consequences:
- Fear over investigations and treatments (pain, side-effects).
- Poorer prognosis, worry about survival .
- Fear of recurrence.

Standard Gamble
You have the opportunity to gamble your current health state (as described above) for perfect health, but you also risk death if you take the gamble. It is important to remember about your current health scenario, and your current symptoms, treatments, side-effects and prognosis. Below, you will see 2 buttons. When you click on the right button, the chances of perfect health and death vary, these are examples to help you understand. In the text box below, please enter in the risk of death that you are willing to take in order to avoid this health state.

5/5/2015

Health State 4: Referred to breast clinic for triple assessment - Stage 4 Breast Cancer found

What it means:
You are referred for **triple assessment**, and cancer is found in your breast, which has also spread to one or more organs in the body. At this stage the cancer cannot be cured, but treatment can slow down the disease and let you live longer.

Physical consequences:
- You have undergone **triple assessment**.
- The main treatment is **chemotherapy.**
- You may have surgery or **radiation therapy** to treat pain and other symptoms.
- Possible **hormonal and/or biological therapy**, depending on type of cancer.

Emotional consequences:
- Fear due to poor prognosis and treatments.
- Fear of dying.

VAS Scale
Assume that you are currently experiencing the health state you just read. On a scale of 0 to 100 where 0 is death and 100 perfect health where would you rate this health state. Please use the scale below. When you are happy with your selection please click 'Completed'.

Health State 4: Referred to breast clinic for triple assessment - Stage 4 Breast Cancer found

What it means:
You are referred for **triple assessment**, and cancer is found in your breast, which has also spread to one or more organs in the body. At this stage the cancer cannot be cured, but treatment can slow down the disease and let you live longer.

Physical consequences:
- You have undergone **triple assessment**.
- The main treatment is **chemotherapy.**
- You may have surgery or **radiation therapy** to treat pain and other symptoms.
- Possible **hormonal and/or biological therapy**, depending on type of cancer.

Emotional consequences:
- Fear due to poor prognosis and treatments.
- Fear of dying.

Standard Gamble
You have the opportunity to gamble your current health state (as described above) for perfect health, but you also risk death if you take the gamble. It is important to remember about your current health scenario, and your current symptoms, treatments, side-effects and prognosis. Below, you will see 2 buttons. When you click on the right button, the chances of perfect health and death vary, these are examples to help you understand. In the text box below, please enter in the risk of death that you are willing to take in order to avoid this health state.

5/5/2015

Health State 5: Referred to breast clinic for triple assessment - No cancer found

What it means:
You were referred for **triple assessment** and no cancer was found.

Physical consequences:
•You have undergone **triple assessment**.

Emotional consequences:
• Fear of investigations.
• Fear that the results were wrong and that you could still have cancer.

VAS Scale
Assume that you are currently experiencing the health state you just read. On a scale of 0 to 100 where 0 is death and 100 perfect health where would you rate this health state. Please use the scale below.
When you are happy with your selection please click 'Completed'.

Health State 5: Referred to breast clinic for triple assessment - No cancer found

What it means:
You were referred for **triple assessment** and no cancer was found.

Physical consequences:
•You have undergone **triple assessment**.

Emotional consequences:
•Fear of investigations
•Fear that the results might be wrong and that you could still have cancer.

Standard Gamble
You have the opportunity to gamble your current health state (as described above) for perfect health, but you also risk death if you take the gamble. It is important to remember about your current health scenario, and your current symptoms, treatments, side-effects and prognosis. Below, you will see 2 buttons. When you click on the right button, the chances of perfect health and death vary, these are examples to help you understand. In the text box below, please enter in the risk of death that you are willing to take in order to avoid this health state.

5/5/2015

Health State 6: Watchful waiting, recurrence/worsening of symptoms, referred to breast clinic for triple assessment - Stage 1 Breast Cancer found

What it means
After a period of **watchful waiting** with your GP, your symptoms were not resolving/becoming worse. You were referred for **triple assessment** and breast cancer has been diagnosed that is confined to the breast and may involve a small number of **lymph nodes**. This is an early stage of breast cancer.

Physical consequences:
- You have undergone **triple assessment**.
- You will have surgery (**lumpectomy, mastectomy, lymph node removal**).
- **Chemotherapy** and/or **radiation therapy** to prevent the cancer from coming back.
- **Hormonal therapy** or **biological therapy** may be given, depending on the type of cancer cells.

Emotional Consequences:
- Anger/worry about delay in diagnosis.
- Fear over investigations and treatments (pain, side-effects).
- Anxiety or depression associated with diagnosis.
- Worry that cancer may spread or will not be fully cured.

__VAS Scale__
Assume that you are currently experiencing the health state you just read. On a scale of 0 to 100 where 0 is death and 100 perfect health where would you rate this health state. Please use the scale below. When you are happy with your selection please click 'Completed

Health State 6: Watchful waiting, recurrence/worsening of symptoms, referred to breast clinic for triple assessment - Stage 1 Breast Cancer found

What it means
After a period of **watchful waiting** with your GP, your symptoms were not resolving/becoming worse. You were referred for **triple assessment** and breast cancer has been diagnosed that is confined to the breast and may involve a small number of **lymph nodes**. This is an early stage of breast cancer.

Physical consequences:
- You have undergone **triple assessment**.
- You will have surgery (**lumpectomy, mastectomy, lymph node removal**).
- **Chemotherapy** and/or **radiation therapy** to prevent the cancer from coming back.
- **Hormonal therapy** or **biological therapy** may be given, depending on the type of cancer cells.

Emotional Consequences:
- Anger/worry about delay in diagnosis.
- Fear over investigations and treatments (pain, side-effects).
- Anxiety or depression associated with diagnosis.
- Worry that cancer may spread or will not be fully cured

__Standard Gamble__
You have the opportunity to gamble your current health state (as described above) for perfect health, but you also risk death if you take the gamble. It is important to remember about your current health scenario, and your current symptoms, treatments, side-effects and prognosis. Below, you will see 2 buttons. When you click on the right button, the chances of perfect health and death vary, these are examples to help you understand. In the text box below, please enter in the risk of death that you are willing to take in order to avoid this health state.

5/5/2015

Health State 7: Watchful waiting, recurrence/worsening of symptoms, referred to breast clinic for triple assessment - Stage 2 Breast Cancer found

What it means:
After a period of **watchful waiting**, your symptoms were not resolving/became worse. You were referred for **triple assessment**, and cancer was found, which is contained within the breast. It may also be in surrounding **lymph nodes.**

Physical consequences:
- You have undergone **triple assessment**.
- You will have surgery (**lumpectomy, mastectomy, lymph node removal**).
- You will have **radiation therapy.**
- You may have **chemotherapy**, and/or **hormonal therapy,** depending on the type of cancer

Emotional consequences:
- Anger/worry about delay in diagnosis.
- Fear over investigations and treatments (pain, side-effects).
- Anxiety or depression due to cancer diagnosis.
- Fear of recurrence.

VAS Scale
Assume that you are currently experiencing the health state you just read. On a scale of 0 to 100 where 0 is death and 100 perfect health where would you rate this health state. Please use the scale below.
When you are happy with your selection please click 'Completed

Health State 7: Watchful waiting, recurrence/worsening of symptoms, referred to breast clinic for triple assessment - Stage 2 Breast Cancer found

What it means:
After a period of **watchful waiting**, your symptoms were not resolving/became worse. You were referred for **triple assessment**, and cancer was found, which is contained within the breast. It may also be in surrounding **lymph nodes.**

Physical consequences:
- You have undergone **triple assessment**.
- You will have surgery (**lumpectomy, mastectomy, lymph node removal**).
- You will have **radiation therapy.**
- You may have **chemotherapy**, and/or **hormonal therapy,** depending on the type of cancer.

Emotional consequences:
- Anger/worry about delay in diagnosis.
- Fear over investigations and treatments (pain, side-effects).
- Anxiety or depression due to cancer diagnosis.
- Fear of recurrence.

Standard Gamble
You have the opportunity to gamble your current health state (as described above) for perfect health, but you also risk death if you take the gamble. It is important to remember about your current health scenario, and your current symptoms, treatments, side-effects and prognosis. Below, you will see 2 buttons. When you click on the right button, the chances of perfect health and death vary, these are examples to help you understand. In the text box below, please enter in the risk of death that you are willing to take in order to avoid this health state.

5/5/2015

Health State 8: Watchful waiting, recurrence/worsening of symptoms, referred to breast clinic for triple assessment - Stage 3 Breast Cancer found

What it means:
After a period of **watchful waiting**, your symptoms have not resolved/become worse. You are referred for **triple assessment**. Cancer is found in the breast, it has spread into surrounding tissues, and many **lymph nodes** are involved.

Physical consequences:
- You have undergone **triple assessment.**
- You will have a surgery (**lumpectomy, mastectomy, lymph node removal**).
- You will probably have **radiation therapy** and/or **chemotherapy.**
- You may have **hormonal** or **biological therapy**, depending on the type of cancer cells.

Emotional consequences:
- Anger/worry about delay in diagnosis.
- Fear over investigations and treatments (pain, side-effects).
- Poorer prognosis, worry about survival.
- Fear of recurrence.

VAS Scale
Assume that you are currently experiencing the health state you just read. On a scale of 0 to 100 where 0 is death and 100 perfect health where would you rate this health state. Please use the scale below. When you are happy with your selection please click 'Completed

Health State 8: Watchful waiting, recurrence/worsening of symptoms, referred to breast clinic for triple assessment - Stage 3 Breast Cancer found

What it means:
After a period of **watchful waiting**, your symptoms have not resolved/become worse. You are referred for **Triple assessment**. Cancer is found in the breast, it has spread into surrounding tissues, and many **lymph nodes** are involved.

Physical consequences:
- You have undergone **triple assessment.**
- You will have a surgery (**lumpectomy, mastectomy, lymph node removal**).
- You will probably have **radiation therapy** and/or **chemotherapy.**
- You may have **hormonal** or **biological therapy**, depending on the type of cancer cells.

Emotional consequences:
- Anger/worry about delay in diagnosis.
- Fear over investigations and treatments (pain, side-effects).
- Poorer prognosis, worry about survival.
- Fear of recurrence.

Standard Gamble
You have the opportunity to gamble your current health state (as described above) for perfect health, but you also risk death if you take the gamble. It is important to remember about your current health scenario, and your current symptoms, treatments, side-effects and prognosis. Below, you will see 2 buttons. When you click on the right button, the chances of perfect health and death vary, these are examples to help you understand. In the text box below, please enter in the risk of death that you are willing to take in order to avoid this health state.

Health State 9: Watchful waiting, recurrence/worsening of symptoms, referred to breast clinic for triple assessment - Stage 4 Breast Cancer found

What it means:
After a period of **watchful waiting** with your GP, your symptoms have not resolved/became worse. You are referred for **triple assessment**, and cancer is found in your breast, which has also spread to one or more organs in the body. At this stage the cancer cannot be cured, but treatment can slow down the disease and let you live longer.

Physical consequences:
- You have undergone **triple assessment**.
- The main treatment is **chemotherapy**.
- You may have surgery or **radiation therapy** to treat pain and other symptoms.
- Possible **hormonal** and/or **biological therapy**, depending on type of cancer.

Emotional Consequences:
- Anger/worry about delay in diagnosis.
- Fear due to poor prognosis and treatments.
- Fear of dying.

VAS Scale
Assume that you are currently experiencing the health state you just read. On a scale of 0 to 100 where 0 is death and 100 perfect health where would you rate this health state. Please use the scale below. When you are happy with your selection please click 'Completed'.

Health State 1
0 ___|________________________ 100

Health State 9: Watchful waiting, recurrence/worsening of symptoms, referred to breast clinic for triple assessment - Stage 4 Breast Cancer found

What it means:
After a period of **watchful waiting** with your GP, your symptoms have not resolved/became worse. You are referred for **triple assessment**, and cancer is found in your breast, which has also spread to one or more organs in the body. At this stage the cancer cannot be cured, but treatment can slow down the disease and let you live longer.

Physical consequences:
- You have undergone **triple assessment**.
- The main treatment is **chemotherapy**.
- You may have surgery or **radiation therapy** to treat pain and other symptoms.
- Possible **hormonal** and/or **biological therapy**, depending on type of cancer.

Emotional Consequences:
- Anger/worry about delay in diagnosis.
- Fear due to poor prognosis and treatments.
- Fear of dying.

Standard Gamble.
You have the opportunity to gamble your current health state (as described above) for perfect health, but you also risk death if you take the gamble. It is important to remember about your current health scenario, and your current symptoms, treatments, side-effects and prognosis. Below, you will see 2 scenarios. When you click on the right button, the chances of perfect health and death vary, these are examples to help you understand. In the text box below, please enter in the risk of death that you are prepared to take in order to avoid this health state.

5/5/2015

Health State 10: Watchful waiting, referred to breast clinic for triple assessment - No cancer found

What it means
After a period **watchful waiting** with your GP. Your symptoms were not resolving/became worse. You are referred for **triple assessment** and no cancer was found.

Physical Consequences:
• You have undergone **triple assessment.**

Emotional Consequences:
• Fear of investigations.
• Worry about the delay in diagnosis.
• Fear the results might be wrong and that you still could have cancer.

VAS Scale
Assume that you are currently experiencing the health state you just read. On a scale of 0 to 100 where 0 is death and 100 perfect health where would you rate this health state. Please use the scale below. When you are happy with your selection please click 'Completed'.

Health State 10 : Watchful waiting, referred to breast clinic for triple assessment - No cancer found

What it means
After a period **watchful waiting** with your GP. Your symptoms were not resolving/became worse. You are referred for **triple assessment** and no cancer was found.

Physical Consequences:
• You have undergone **triple assessment.**

Emotional Consequences:
• Fear of investigations.
• Worry about the delay in diagnosis.
• Fear the results may be wrong and that you still could have cancer.

Standard Gamble.
You have the opportunity to gamble your current health state (as described above) for perfect health, but you also risk death if you take the gamble. It is important to remember about your current health scenario, and your current symptoms, treatments, side-effects and prognosis. Below, you will see 2 buttons. When you click on the right button, the chances of perfect health and death vary, these are examples to help you understand. In the text box below, please enter in the risk of death that you are willing to take in order to avoid this health state.

For peer review only - http://bmjopen.bmj.com/site/about/guidelines.xhtml

5/5/2015

Health State 11: Watchful waiting, resolution of symptoms, no referral needed.

What it means:
After a period of **watchful waiting**, symptoms have stopped or are resolving within 3 month's follow up with GP. You do not need further investigation.

Physical Consequences:
•.No further tests are needed.

Emotional Consequences:
• Relief that symptoms are not serious.
• Worry that there might be a cancer that has been missed.

VAS Scale
Assume that you are currently experiencing the health state you just read. On a scale of 0 to 100 where 0 is death and 100 perfect health where would you rate this health state. Please use the scale below. When you are happy with your selection please click 'Completed'.

Health State 11: Watchful waiting, resolution of symptoms, no referral needed.

What it means:
After a period of **watchful waiting**, symptoms have stopped or are resolving within 3 month's follow up with GP. You do not need further investigation.

Physical Consequences:
•.No further tests are needed.

Emotional Consequences:
• Relief that symptoms are not serious.
• Worry that there might be a cancer that has been missed.

Standard Gamble.

You have the opportunity to gamble your current health state (as described above) for perfect health, but you also risk death if you take the gamble. It is important to remember about your current health scenario, and your current symptoms, treatments, side-effects and prognosis. Below, you will see 2 buttons. When you click on the right button, the chances of perfect health and death vary, these are examples to help you understand. In the text box below, please enter in the risk of death that you are willing to take in order to avoid this health state.

BMJ Open

Quantifying patient preferences for symptomatic breast clinic referral: a decision analysis study

Journal:	*BMJ Open*
Manuscript ID	bmjopen-2017-017286.R1
Article Type:	Research
Date Submitted by the Author:	16-Aug-2017

Complete List of Authors:	Quinlan, Aisling; Royal College of Surgeons in Ireland, Department of General Practice<br>O'Brien, Kirsty; Royal College of Surgeons in Ireland, Department of General Practice<br>Galvin, Rose; University of Limerick, 3Department of Clinical Therapies<br>Hardy, Colin; Royal College of Surgeons in Ireland, Department of General Practice<br>McDonnell, Ronan; Royal College of Surgeons in Ireland, Department of General Practice<br>Joyce, Doireann; Royal College of Surgeons in Ireland, Department of General Practice<br>McDowell, Ronald; Royal College of Surgeons in Ireland, HRB Centre for Primary Care Research<br>Aherne, Emma; Royal College of Surgeons in Ireland, Department of General Practice<br>Keogh, Claire; RCSI, HRB Centre for Primary Care Research<br>O'Sullivan, Katriona; University of Dublin Trinity College<br>Fahey, Tom; Royal College of Surgeons in Ireland, Department of General Practice

**Primary Subject Heading**:	Evidence based practice
Secondary Subject Heading:	Diagnostics, Evidence based practice, Oncology
Keywords:	EPIDEMIOLOGY, ONCOLOGY, Quality in health care < HEALTH SERVICES ADMINISTRATION & MANAGEMENT

SCHOLARONE™
Manuscripts

Quantifying patient preferences for symptomatic breast clinic referral: a decision analysis study

Aisling Quinlan[1], BSc, Kirsty K O'Brien[1], PhD, Rose Galvin[1,3], PhD, Colin Hardy[1], MSc, Ronan McDonnell[1], PhD, Doireann Joyce[2], MB BCh BAO, Ronald D.McDowell[1], PhD, Emma Aherne[1],MB BCh BAO, Claire Keogh[1], PhD, Katriona O'Sullivan[1,4], PhD, Tom Fahey[1], MD

[1]HRB Centre for Primary Care Research, Department of General Practice, Royal College of Surgeons in Ireland (RCSI), 123 St.Stephens Green, Dublin 2, Republic of Ireland.

[2]National University of Ireland Galway, University Road, Galway. Republic of Ireland.

[3]Department of Clinical Therapies, University of Limerick, Limerick.

[4]School of Computer Science and Statistics, Trinity College Dublin.

*Corresponding Author

Tom Fahey, HRB Centre for Primary Care Research, Department of General Practice, Royal College of Surgeons in Ireland, 123 St. Stephen's Green, Dublin 2, Republic of Ireland.

Email: tomfahey@rcsi.ie

Word count:

For peer review only

Abstract

Objectives: Decision analysis study that incorporates patient preferences and probability estimates to investigate the impact of women's preferences for referral or an alternative strategy of watchful waiting if faced with symptoms that could be due to breast cancer.

Setting: Community-based study.

Participants: Asymptomatic women aged 30-60 years.

Interventions: Participants were presented with 11 health scenarios that represent the possible consequences of symptomatic breast problems. Participants were asked the risk of death that they were willing to take in order to avoid the health scenario using the standard gamble (SG) utility method. This process was repeated for all 11 health scenarios. Formal decision analysis for the preferred individual decision was then estimated for each participant.

Primary outcome measure: The preferred diagnostic strategy, either watchful waiting or referral to a breast clinic. Sensitivity analysis was used to examine how each varied according to changes in the probabilities of the health scenarios.

Results: A total of 35 participants completed the interviews, with median age 41 years (Interquartile range 35 to 47 years). The majority of the study sample were employed (n=32, 91.4%), with a third-level education (n=32, 91.4%) and with knowledge of someone with breast cancer (n=30, 85.7%). When individual preferences were accounted for, 25 (71.4%) patients preferred watchful waiting to referral for triple assessment as their preferred initial diagnostic strategy. Sensitivity analysis shows that referral for triple assessment becomes the dominant strategy at the upper probability estimate (18%) of breast cancer in the community.

Conclusions: Watchful waiting is an acceptable strategy for most women who present to their GP with breast symptoms. These findings suggest that current referral guidelines should take more explicit account of women's preferences in relation in terms of the initial diagnostic strategy for symptomatic breast problems.

For peer review only

Strengths and limitations of this study

Strength of this study is that it accounts for women’s preferences in relation to a watchful waiting or immediate referral strategy when faced with symptoms that could be due to breast cancer.

Weakness is that preferences were elicited in asymptomatic women. Women with symptoms may express different preferences.

The impact of eliciting preferences should be done in the context of a randomized trial of a decision aid that uses decision analysis as a form of shared decision making for women with symptoms that could be due to breast cancer.

Taking account of women’s preferences might mean that significantly fewer women would be referred to symptomatic breast clinics with a consequent saving in investigations and costs, and reducing the burden of “medicalization” in women with benign breast disease.

For peer review only

For peer review only - http://bmjopen.bmj.com/site/about/guidelines.xhtml

Introduction

Between 2009-2011, it was reported that 31% of cancers in Irish women were due to breast cancer, making it the most prevalent cancer in Irish women [1]. In response, the National Cancer Control Programme (NCCP) clinical guidelines were introduced to streamline the referral process to symptomatic breast units. At present, GPs act as gatekeepers for referral of women with breast symptoms. Referral to symptomatic breast clinics results in triple assessment: a three step process comprising of clinical, radiological and histological examination after fine needle aspiration [2]. Over the past ten years, the number of women referred to symptomatic breast clinics in Ireland has significantly increased, despite the fact there has been no rise in breast cancer incidence [2]. In 2006, there were 23,575 new referrals with 2137 breast cancers diagnosed, with 32,249 referalls and 1879 breast cancers diagnosed in 2009 [3],and 37,631 new referalls in 2010 with 2012 new breast cancers diagnosed [4]. Consequently the malignant;benign ratio has altered from 1:10 in 2006, to 1:16 in 2009 to 1:18 in 2010 [4]. The rise in these referrals for triple assessment without an increase in breast cancer diagnosis means that more women are being exposed to invasive diagnostic investigations.

Clinical prediction rules (CPRs) are clinical tools that quantify the independent impact of factors from a patients history, physical examination and diagnostic tests and stratify patients according to the probability of having a target disorder [5]. Recently, CPRs have been derived and validated to calculate a patient's risk of breast cancer [6, 7], and have been proposed as methods to discriminate between patients at high risk of breast cancer from low risk patients. Such an approach may serve to decrease the number of unnecessary referrals to symptomatic breast clinics in women with a low probability of breast cancer. Patients who are considered low risk could, as an alternative, undergo a watchful waiting strategy (up to 6-8 weeks) with their GP. However, it is unclear from the current literature if patients who are considered low risk would find this acceptable.

Decision analysis is a technique to aid decision making when uncertainty exists over the balance between benefits and risks of treatment [8]. Decision trees have been utilised to assess how patient's preferences impact on diagnosis and treatment for conditions such as ovarian cancer

For peer review only - http://bmjopen.bmj.com/site/about/guidelines.xhtml

[9] and atrial fibrillation [10]. To date, little is known about how women who have symptomatic breast problems feel about the options of immediate referral or watching waiting in terms of referral to a symptomatic breast clinic. The aim of this study is assess the impact of patient preferences for immediate referral to a specialist breast clinic or watchful waiting in primary care. We developed a decision tree and elicited patients preferences for these alternative diagnostic strategies by combining individual preferences with probability estimates of breast cancer using decision analysis.

Methodology

Study design

This study utilized decision analysis in the form of utility assessment and folding back a decision tree. The STROBE standardized reporting guidelines were followed to ensure the standardized conduct and reporting of the research. Ethical approval was received from the Research Ethics Committee at the Royal College of Surgeons in Ireland.

Developing the decision tree and health scenarios

Literature searches were conducted in medical databases such as PubMed, the Cochrane library and Web of Science to assist with the development of the decision tree and health scenarios. A specialist registrar with clinical experience in a symptomatic breast clinic was also consulted during this development phase. Following these consultations, the decision tree was constructed to portray the temporal course of the two diagnostic options (Figure 1). The decision tree had one decision node with two diagnostic branches (a choice between watchful waiting or immediate referral), nine probability or chance nodes and eleven health outcome nodes. In accordance with the literature the tree was designed such that only two branches emanated from each chance node [11]. The number of outcome nodes differed between the two branches of the tree in order to accommodate the additional step (an observation period before breast cancer assessment among patients who return to their GP) in the watchful waiting branch of the tree. It is not always possible or appropriate for each branch of a decision

For peer review only

For peer review only - http://bmjopen.bmj.com/site/about/guidelines.xhtml

tree to have the same number of outcome nodes as this will be dependent on the nature and temporal order of events in that branch [12]. The decision tree is “balanced” as each branch has advantages and disadvantages and hence it is not possible to readily identify a treatment path that is ideal in all respects [11].

A total of eleven health scenarios were constructed, one for each outcome node. For this study the standard gamble (SG) method of utility assessment was used [13] [14]. Briefly, participants presented with a description of each health state, its physical consequences and emotional consequences, and were given the opportunity to remain in the health state or to gamble it for perfect health, but with the risk of death if they took the gamble. Participants were presented with differing probabilities showing how the chance of perfect health and death varied with each other for each health state (the “ping-pong method”). The utility value was one minus the risk of death they were willing to take in order to avoid the health state described. The 11 health states were presented in a random order. Details of the health states and the standard gamble scenarios seen by the participants are provided in the supplementary materials section.

Probability estimates for each chance node were obtained from the medical literature [7,15-28]. Where possible, relevant probability estimates for each chance node were combined using random effects meta-analysis (Table 1). The same probabilities were used for each participant. It was not possible to estimate from the literature the probability of a diagnosis of breast cancer among patients who returned to their doctor following a period of watchful waiting. Clinical experience suggested this would be several percentage points higher in absolute terms than the probability of a diagnosis of breast cancer following RTA and an estimate of 10% was used.

Participants and setting

Interviews were conducted over a six month period in Dublin from October 2014 to April 2015. Asymptomatic women aged 30-60 years were invited to participate in the study. Participants

For peer review only - http://bmjopen.bmj.com/site/about/guidelines.xhtml

were recruited using staff notice boards, social media and snowball sampling. The majority of interviews were carried out in a meeting room in the research building. Some interviews were conducted in peoples' place of work as this was more convenient for them. Written informed consent was obtained from all participants prior to participation. The interviews were conducted with a researcher present, who was available to answer any questions and to obtain feedback about the interview. Prior to the interview, participants were given a baseline demographics questionnaire and a multiple-choice questionnaire to assess their knowledge of breast cancer. As this is a feasibility study for a proposed randomized controlled trial, no formal sample size was calculated.

Utility assessment

A computer-based assessment programme which presented and recorded participant responses was designed. This programme incorporated an introduction, an explanation of the interview process, and presentation of each clinical scenario in random order using the standard gamble method of utility assessment. An example of the standard gamble was included for orientation and definitions were provided for any medical terminology used. The purpose of using a computer programme was to allow the interviewer to limit their interaction with each participant, standardize utility assessment and minimize interviewer bias. A pilot study was carried out and modifications made to the computer programme and utility assessment process. See supplementary table of health scenarios.

Ethical approval and data Protection

The data from the computer programme was stored with a participant ID only and saved to an encrypted password protected desktop computer. To protect patient identity, a MS excel spreadsheet was created; containing just patient names and identification numbers, and this was stored in a secured, password protected folder on the server and kept separate to the data from the interview.

For peer review only - http://bmjopen.bmj.com/site/about/guidelines.xhtml

Decision analysis

The utility for each outcome, “a number assigned to the quality of life a patient would attach to a particular outcome on a defined scale “ [12], was calculated as 1-the risk of death (expressed as a fraction) a participant was willing to take in order to avoid the health scenario as described. For each participant, an expected utility score for each diagnostic option was obtained by multiplying and summing the relevant probabilities and utilities. The branch which had the highest expected utility score was determined to be the preferred diagnostic strategy- watchful waiting or referral for triple assessment.

Sensitivity analysis was used to determine the robustness of the conclusion drawn from the decision tree [29]. For our study each of the probability estimates derived from the literature was allowed to vary between the lower and upper limits of the 95% confidence interval for that estimate, in order to assess the extent to which the preferred diagnostic strategy changed. The difference between the number of patients preferring watchful waiting using the upper limit of the 95% confidence interval and the number of patients preferring watchful waiting using the lower limit of the 95% confidence interval for each probability estimate (the swing) was squared and totalled; the impact of varying each of the probability estimates was measured as a percentage of the total squared-swing. The sensitivity analyses accounted for the fact that some probability estimates were used more than once by incorporating the conditioning event into the definition of the variables under consideration [30]. An additional two-way sensitivity analysis explored how women’s preferred diagnostic option changed as the probability of diagnosis following watchful waiting varied relative to the probability of diagnosis following referral and triple assessment.

Standard descriptive statistics were used to summarise the sample. Results were held to be significant if they refer to statistical significance on a two-sided test evaluated at the 5% level. All analyses were performed using STATA 13 [31].

For peer review only - http://bmjopen.bmj.com/site/about/guidelines.xhtml

Results

A total of 36 women participated, with 35 interviews completed in full. The median age of participants was 41 years (interquartile range (IQR) 35 to 47 years). The demographic details of participants are described in Table 2. The majority of participants were employed (32, 91.4%), had a third-level education (32, 91.4%) and knew someone with breast cancer (30, 85.7%). Less than half the study sample (15, 42.9%) reported having a prior mammography. There was borderline evidence of a negative correlation between participants' self-reported knowledge of breast cancer and their actual knowledge based on responses to a multiple-choice questionnaire (p=0.06).

When patient preferences were taken into account, 25 patients (71.4%, 95%CI 19%, 30%) preferred watchful waiting to referral as the initial diagnostic strategy. Sensitivity analysis showed that the overall preferred diagnostic strategy changed to referral when the probability of breast cancer following a period of watchful waiting increased; only ten women favoured watchful waiting when the probability of breast cancer following a period of watchful waiting had increased to 18% (Table 3). Swings in the group preference according to changes in the probabilities associated with each of the stage of breast cancer were small in comparison (Table 3).

In terms of two-way sensitivity analysis more than half the participating women still preferred watchful waiting when the probability of a diagnosis of breast cancer following a period of watchful waiting was 2.25 times as high as the probability of diagnosis following referral and triple assessment. (Figure 2) This pattern remained consistent irrespective of the probability of breast cancer following referral and triple assessment, estimated at levels of 4%, 6%, 8% and 10%.

For peer review only - http://bmjopen.bmj.com/site/about/guidelines.xhtml

Discussion

Summary of main findings

This study shows that a strategy of watching waiting in women with breast symptoms in primary care could be considered as an acceptable alternative to immediate referral to a symptomatic breast clinic, but this strategy of shared decision making using decision analysis should be tested in a randomized controlled trial. Our observational findings suggest that women's own preferences should form an important element of the decision to refer to a symptomatic breast clinic and that these preferences should be accounted as part of a shared decision making process. Sensitivity analysis shows that as the probability of breast cancer increases, the diagnostic strategy of referral for triple assessment dominates. Two way sensitivity analysis shows that, across the likely probability range of breast cancer under a watchful waiting strategy, referral for triple assessment becomes the dominant strategy once the probability of breast cancer is two and a half times more likely than under a watchful waiting strategy.

Results in the context of the current literature

Although utility assessment has been used to evaluate the acceptability of various clinical states and treatment regimes in the breast cancer literature [32-34] it has not been used to examine the acceptability of watchful waiting in low risk patients presenting with signs and symptoms of possible breast cancer. A previous study that assessed the effects of alternative terms for ductal carcinoma in-situ (DCIS). found that watchful waiting is acceptable to patients provided that research shows that watchful waiting is a safe and effective option [35]. The preliminary findings from our study suggest that women may beprepared to accept a conservative, non-invasive strategy of watchful waiting when the probability of breast cancer is low.

Watchful waiting has also been described as a key technique for more effective management of other cancers such as prostate cancer [36]. In prostate cancer the use of PSA testing has led to an increase in the incidence of prostate cancer but not a decrease in the mortality rate, suggesting that PSA testing often identifies low risk cancers [37]. Given the serious and

For peer review only - http://bmjopen.bmj.com/site/about/guidelines.xhtml

common side effects associated with treatment in prostate cancer some studies have shown that some men prefer watchful waiting to treatment options [38]. In our study the side effects of further diagnostic testing (triple assessment) are less onerous than treatment of localized prostate cancer, but many women would experience high levels of anxiety and stress upon referral to a symptomatic breast clinic. This study suggests that many women would find it more acceptable to undergo watchful waiting for a period rather than participate in diagnostic testing when their risk of breast cancer is low.

A qualitative study examining women's views on over-diagnosis in breast cancer screening showed that 50% of the population study felt they needed to make more careful personal decisions about screening and would consider watchful waiting as an alternative route if they were found to have a low risk cancer [39]. Like our study watchful waiting was found to be an acceptable strategy when the risk involved is low and the health outcomes are positive.

Strengths and limitations

This study has several strengths. We used an evidence-based approach, quantifying probabilities from the literature, and developing health states that incorporate the common features of breast cancer. We tested and modified the wording of the health states and the computer programme in a pilot group of participants and refined our protocol based on feedback from these participants. We tried to limit any bias caused by the interviewer by using a computer programme and providing the participant with written definitions and information. Examples of the standard gamble were given to the participants prior to the interview, so participants had the opportunity to familiarize themselves with the method. Although the sample was relatively small, it included participants with a range of ages and knowledge of breast cancer. We acknowledge that the sample of participants is not representative of the population at large; that women were asymptomatic and that preferences may change or be different when faced with possible symptoms of breast cancer- a breast lump or nipple change. However, our results suggest that when preferences about a watchful waiting strategy is elicited, many women are happy to adopt this approach. Based on these provisional results, we

For peer review only - http://bmjopen.bmj.com/site/about/guidelines.xhtml

hope to develop a decision aid for women with symptomatic breast problems and test it value in a randomized controlled trial.

Clinical and policy Implications

The current referral process leads to many patients who are at low risk of breast cancer being referred to busy symptomatic breast clinics, with a risk of iatrogenic harm, increased patient anxiety and over-medicalizing of a self-limiting condition. With more emphasis on the development of clinical prediction rules, it will be possible to identify and quantify each individual women's risk of breast cancer. A recent study derived a clinical prediction rule (CPR) for predicting the risk of breast cancer for women presenting to their GP with breast symptoms. [7] A total of 6,590 patients were included in the derivation study and 4.9% were diagnosed with breast cancer. Independent clinical predictors for breast cancer were: increasing age by year (adjusted OR 1.08, 95%CI 1.07-1.09); presence of a lump (5.63, 95%CI 4.2-7.56); nipple change (2.77, 95%CI 1.68-4.58) and nipple discharge (2.09, 95% CI 1.1-3.97). Validation of the rule (n=911) also shows that the probability of breast cancer is higher with an increasing number of these independent clinical variable. By incorporating patient preferences with an individual's probability of breast cancer from a CPR, a strategy of watchful waiting is acceptable, cost effective and likely to avoid considerable iatrogenic harm. Clinical practice guidelines should incorporate probability estimates of breast cancer based on CPRs and urge elicitation of patient preference for alternative diagnostic options- watchful waiting or referral. More formally, decision aids that could be developed in conjunction with the symptomatic breast CPRs to enable the alternative options of watchful waiting or referral based on individual preferences.

Conclusion

This study suggests that watchful waiting may be an acceptable diagnostic strategy for women who present to their general practitioner with breast symptoms. These findings should be

For peer review only - http://bmjopen.bmj.com/site/about/guidelines.xhtml

validated in a larger and more comprehensive study of patient preferences in women with breast symptoms in a randomized controlled trial. Clinical practice guidelines should reflect the importance of eliciting women's preferences for watchful waiting as an alternative to referral to a symptomatic breast clinic, particularly in women with low risk clinical features.

Competing interests

The authors declare that they have no competing interests.

Authors' contributions

All authors were involved in the study conception and design. AQ, RG and KOB acquired data for analysis. RmcD designed the computer-based utility analysis programme. CH and RDMcD performed statistical analysis. AQ interpreted the data and drafted the paper. KOB, RG, RDMcD and TF critically revised the draft manuscript. All authors read and approved the final manuscript.

Funding statement

Health Research Board of Ireland grant number HRC-2007-1.

Data sharing statement

We are happy to share the data from this study

Figure legends:

For peer review only - http://bmjopen.bmj.com/site/about/guidelines.xhtml

Figure 1. Decision tree for health states in the treatment of breast cancer with utility values (median (interquartile range)) for each state

Figure 2 Two way sensitivity analysis according to probability under referral and triple asesssment (four levels) at incremental probability levels under watchful waiting

For peer review only

For peer review only - http://bmjopen.bmj.com/site/about/guidelines.xhtml

References

1. National Cancer Registry: **Cancer in Ireland 1994-2011: Annual report of the National Cancer Registry 2014**. In. Cork: National Cancer Registry; 2014.
2. O'Rourke N: **Review of Referral Patterns and Triage Processes in Symptomatic Breast Units - a Hospital Perspective**. In.: HSE; 2012: 72.
3. National Cancer Control Programme: **Review of activity in the symptomatic breast clinics in the eight designated centres, 2005 and 2006 compared with 2009 year to date.** . In.; 2009.
4. National Cancer Control: **Report on the Symptomatic Breast Disease Key Performance Indicators 2010.** In.; 2011.
5. Keogh C, Wallace E, O'Brien KK, Galvin R, Smith SM, Lewis C, Cummins A, Cousins G, Dimitrov BD, Fahey T: **Developing an international register of clinical prediction rules for use in primary care: a descriptive analysis**. *Annals of family medicine* 2014, **12**(4):359-366.
6. McCowan C, Donnan PT, Dewar J, Thompson A, Fahey T: **Identifying suspected breast cancer: development and validation of a clinical prediction rule**. *The British journal of general practice : the journal of the Royal College of General Practitioners* 2011, **61**(586):e205-214.
7. Galvin R, Joyce D, Downey E, Boland F, Fahey T, Hill AK: **Development and validation of a clinical prediction rule to identify suspected breast cancer: a prospective cohort study**. *BMC cancer* 2014, **14**(1):743.
8. Detsky AS NG, Krahn MD, Naimark D, Redelmeier DA.: **Primer on medical desicion analysis: Part 1 - getting started.** *Medical Decision Making* 1997, **17**(125).
9. Havrilesky LJ BG, Davis DM, Nolte KC, Barnett JC, Myers ER et al. : **Determination of quality of life-related utilities for health states relevant of ovarian cancer diagnosis and treatment.** *Gynecologic Oncology* 2009, **113**:216-220.
10. Protheroe J FT, Montgomery AA, Peters TJ. : **The impact of patients' preferences on the treatment of artial fibrilation: observational study of patient based decision analysis.** *The British Medical Journal* 2000.
11. Allan S, Detsky MD, Naglie G, Krahn MD, Redelmeier DA, Naimark D. **Primer on Medical Decision Analysis: Part 2-Building a Tree**. *Medical Decision Making* 1997. 17(2):126-35.
12. Lee A, Goynt GM, Ho AMH, Keitz S, McGinn T, Wyer P, EBM Teaching Scripts Working Group. **Tips for Teachers of Evidence-based Medicine: Making Sense of Decision Analysis Using a Decision Tree**. *Journal of General Internal Medicine* 2009. 24(5): 642–648
13. von Neumann H, Morgenstern O. **Theory of games and economic behaviour**. 1944. Princeton, NJ: Princeton University Press.
14. Drummond M SM, Torrance GW, O' Brien BJ, Stoddart GL: **Methods for the Economic Evaluation of Health Care Programmes.** Oxford New York: Oxford University Press; 2005.
15. National Cancer Registry: Breast Cancer Incidence, Mortality, Treatment and Survival in Ireland: 1994-2009. National Cancer Registry, Cork, Ireland 2012.
16. Davidson A, Chia S, Olson R, Nichol A, Speers C, Coldman AJ, Bajdik C, Woods R, Tyldesley S: **Stage, treatment and outcomes for patients with breast cancer in British Columbia in 2002: a population-based cohort study**. *CMAJ open* 2013, **1**(4):E134-141.
17. Louwman WJ, Voogd AC, van Dijck JA, Nieuwenhuijzen GA, Ribot J, Pruijt JF, Coebergh JW: **On the rising trends of incidence and prognosis for breast cancer patients diagnosed 1975-2004: a long-term population-based study in southeastern Netherlands**. *Cancer causes & control : CCC* 2008, **19**(1):97-106.

For peer review only - http://bmjopen.bmj.com/site/about/guidelines.xhtml

18. Keegan THM, Press DJ, Tao L, DeRouen MC, Kurian AW, Clarke CA, Gomez SL: **Impact of breast cancer subtypes on3-year survival among adolescent and young adult women**. *Breast Cancer Research* 2013: 15(5):R95.

19. Morris CR, Ramirez CN, Cook SN, Parikh-Patel A, Kizer KW, Bates JH, Snipes KP. **Cancer Stage at Diagnosis**, 2013. Sacramento, CA: California Department of Public Health, California Cancer Registry.

20. Wang Q., Li J, Zheng S, Li J-L, Pang Y, Huang R, Zhang B-N, Zhang B, Yang H-J, Xie X-M, Tang Z-H, Li H, He J-J, Fan J-H, Qiao Y-L. **Breast cancer stage at diagnosis and area-based socioeconomic status: a multicenter 10-year retrospective clinical epidemiological study in China**. *BMC Cancer* 2012. 12:122.

21. Walters S, Maringe C, Butler J, Rachet B, Barrett-Lee P , Bergh J, Boyages J , Christiansen P , Lee M, Wärnberg F, Allemani C , Engholm G , Fornander T, Gjerstorff ML, Johannesen TB, Lawrence G, McGahan CE, Middleton R, Steward J, Tracey E, Turner D, Richards MA, Coleman MP, The ICBP Module 1 Working Group. **Breast cancer survival and stage at diagnosis in Australia, Canada, Denmark, Norway, Sweden and the UK, 2000-2007: a population-based study**. *British Journal of Cancer* 2013. 108, 1195–1208.

22. Campbell C, Durning P, Cheema I, Naisby G. **A simple tool for rapid access to a symptomatic breast clinic**. *European Journal of Surgical Oncology* 2004**.** 30(3):248–251.

23. McCowan C, Donnan PT, Dewar J, Thompson A, Fahey T. **Identifying suspected breast cancer: development and validation of a clinical prediction rule**. *British Journal of General Practice* 2011. 61(585): e205-e214.

24. Cochrane RA, Singhal H, Monypenny IJ, Webster DJ, Lyons K, Mansel RE: **Evaluation of general practitioner referrals to a specialist breast clinic according to the UK national guidelines**. *European journal of surgical oncology : the journal of the European Society of Surgical Oncology and the British Association of Surgical Oncology* 1997, **23**(3):198-201.

25. Nichols S, Waters WE, Wheeler MJ. **Management of female breast disease by Southampton general practioners**. *British Medical Journal* 1980. 281: 1450-1453.

26. Dohrmann PJ, Hugher ESR, McDermott, Price A. **Sympton Duration, tumor staging and survival in patients with carcinoma of the breast**. *Surgery, Gynecology & Obstetrics* 1982. 154: 707-710.

27. Charlson ME. **Delay in the treatment of carcinoma of the breast.** *Surgery, Gynecology & Obstetrics* 1985. 160:393-399.

28. Givio (Interdisciplinary Group for Cancer Care Evaluation) Italy. **Reducing Diagnostic Delay in Breast Cancer: Possible Therapeutic Implications**. *Cancer* 1986. 58:1756-1761

29. Murray DK, Naglie G, Naimark D, Redelmeier DA, Detsky AS. **Primer on Medical Decision Analysis: Part 4-Analyzing the Model and Interpreting the Results**. *Medical Decision Making* 1997. 17(2):142-51.

30. Sendi PP, Clemen RT: **Sensitivity analysis on a chance node with more than two branches**. *Medical decision making : an international journal of the Society for Medical Decision Making* 1999, **19**(4):499-502.

31. StataCorp LP: **STATA User's Guide Release 13**. In. College Station, Texas; 2013.

32. Buyukdamgaci-Alogan G, Elele T, Hayran M, Erman M, Kilickap S: **A decision-analytic model for early stage breast cancer: lumpectomy vs mastectomy**. *Neoplasma* 2008, **55**(3):222-228.

33. Chie WC, Huang CS, Chen JH, Chang KJ: **Utility assessment for different clinical phases of breast cancer in Taiwan**. *Journal of the Formosan Medical Association = Taiwan yi zhi* 2000, **99**(9):677-683.

For peer review only - http://bmjopen.bmj.com/site/about/guidelines.xhtml

34. Sinno H, Izadpanah A, Vorstenbosch J, Dionisopoulos T, Ibrahim AM, Tobias AM, Lee BT, Lin SJ: **Living with a unilateral mastectomy defect: a utility assessment and outcomes study**. *Journal of reconstructive microsurgery* 2014, **30**(5):313-318.
35. McCaffery K, Nickel B, Moynihan R, Hersch J, Teixeira-Pinto A, Irwig L, Barratt A: **How different terminology for ductal carcinoma in situ impacts women's concern and treatment preferences: a randomised comparison within a national community survey**. *BMJ open* 2015, **5**(11):e008094.
36. Mukai TO, Bro F, Olesen F, Vedsted P: **To test or not: a registry-based observational study of an online decision support for prostate-specific antigen tests**. *International journal of medical informatics* 2013, **82**(10):973-979.
37. Esserman L, Shieh Y, Thompson I: **Rethinking screening for breast cancer and prostate cancer**. *Jama* 2009, **302**(15):1685-1692.
38. Hall IJ, Lee Smith J: **Evolution of a CDC Public Health Research Agenda for Low-Risk Prostate Cancer**. *American journal of preventive medicine* 2015, **49**(6s5):S483-s488.
39. Hersch J, Jansen J, Barratt A, Irwig L, Houssami N, Howard K, Dhillon H, McCaffery K: **Women's views on overdiagnosis in breast cancer screening: a qualitative study**. *BMJ (Clinical research ed)* 2013, **346**:f158.

For peer review only

For peer review only - http://bmjopen.bmj.com/site/about/guidelines.xhtml

Table 1 Probability estimates used in the breast cancer decision tree analysis

Treatment path	Outcome	Estimated probability (95% CI)	References
Referral & Triple Assessment (RTA)	Breast cancer diagnosed subsequent to RTA	0.06 (0.05,0.08)	7,22,23,24
	Stage 1 breast cancer among patients diagnosed with breast cancer following RTA	0.34 (0.26,0.42)	15,16,17,18,19,20,21
	Stage 2 breast cancer among patients diagnosed with breast cancer following RTA	0.46 (0.39, 0.52)	15,16,17,18,19,20,21
	Stage 3 breast cancer among patients diagnosed with breast cancer following RTA	0.15 (0.13,0.17)	15,16,17,18,19,20,21
	Stage 4 breast cancer among patients diagnosed with breast cancer following RTA	0.05 (0.04,0.06)	15,16,17,18,19,20,21
Watchful waiting (WW)	Symptoms do not resolve/worsen and patient returns to GP following period of WW	0.34 (0.28,0.41)	25
	Breast cancer diagnosed among patients who return to GP following WW	0.10 (0.05,0.18)	Clinical estimate
	Stage 1 breast cancer among patients diagnosed with breast cancer following WW	0.27 (0.20,0.33)	26,27,28
	Stage 2 breast cancer among patients diagnosed with breast cancer following WW	0.42 (0.36,0.47)	26,27,28
	Stage 3 breast cancer among patients diagnosed with breast cancer following WW	0.20 (0.16,0.22)	26,27,28
	Stage 4 breast cancer among patients diagnosed with breast cancer following WW	0.11 (0.09,0.13)	26,27,28

For peer review only

Table 2. Descriptive statistics for the study sample (n=35 participants)

**Variable**		**Median (Interquartile range)**
Age (years)		41 (35 to 47)
Self-rated knowledge of breast cancer (maximum score 10)		5 (4 to 7)
Actual knowledge of breast cancer (maximum score 7)		3 (2 to 4)
		**Number of participants (%)**
Location	Dublin & surrounds	23 (65.7%)
	West of Ireland	10 (28.6%)
	Overseas	2 (5.7%)
Education	Secondary	3 (8.6%)
	Third level	32 (91.4%)
Employed	Yes	32 (91.4%)
	No	3 (8.6%)
Family carer	Yes	18 (51.4%)
	No	17 (49.6%)
Know someone with breast cancer	Yes	30 (85.7%)
	No	5 (14.3%)
Have had a mammography	Yes	15 (42.9%)
	No	20 (57.1%)

For peer review only - http://bmjopen.bmj.com/site/about/guidelines.xhtml

Table 3. Sensitivity analysis for breast cancer patient treatment preference study

Health scenario	Values for sensitivity analysis			Number of patients preferring WW to RTA			Swing	$Swing^2$	$\%Swing^2$
	Lower limit for probability	Reference probability	Upper limit for probability	Lower limit for probability	Reference probability	Upper limit for probability			
Diagnosis of breast cancer following RTA	0.05	0.06	0.08	23	25	27	4	16	3.21
Stage 1 breast cancer among patients diagnosed following RTA	0.26	0.34	0.42	26	25	21	-5	25	5.02
Stage 2 breast cancer among patients diagnosed following RTA	0.39	0.46	0.52	26	25	24	-2	4	0.80
Stage 3 breast cancer among patients diagnosed following RTA	0.13	0.15	0.17	25	25	25	0	0	0.00
Stage 4 breast cancer among patients diagnosed following RTA	0.04	0.05	0.06	25	25	25	0	0	0.00
Symptoms do not resolve/worsen and patient returns to GP following period of WW	0.28	0.34	0.41	27	25	24	-3	9	1.81
Diagnosis of breast cancer among patients returning to GP after period of watchful waiting	0.05	0.10	0.18	31	25	10	-21	441	88.55
Stage 1 breast cancer among patients diagnosed following WW	0.20	0.26	0.33	25	25	26	1	1	0.20
Stage 2 breast cancer among patients diagnosed following WW	0.36	0.41	0.47	25	25	26	1	1	0.20
Stage 3 breast cancer among patients diagnosed following WW	0.16	0.19	0.22	25	25	25	0	0	0
Stage 4 breast cancer among patients diagnosed following WW	0.09	0.11	0.13	26	25	25	1	-1	0.20

RTA: Referral and Triple Assessment ; WW: Watchful Waiting

Swing=Number of patients preferring WW to RTA (upper limit for probability)- number of patients preferring WW to RTA (lower limit for probability)

$Swing^2$=Swing*Swing

%Swing= $Swing^2$ x100 /(Total $Swing^2$ (=498))

For peer review only

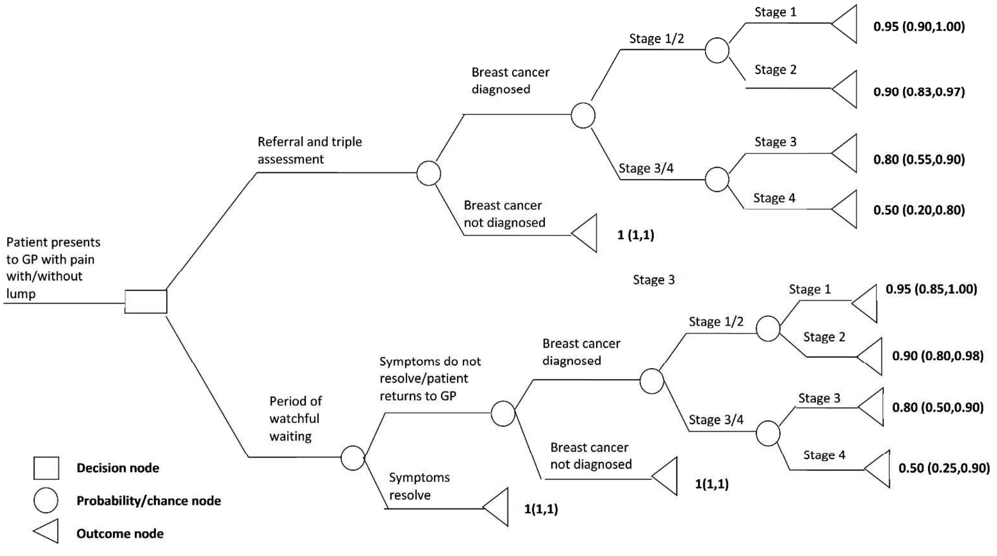

549x300mm (300 x 300 DPI)

review only

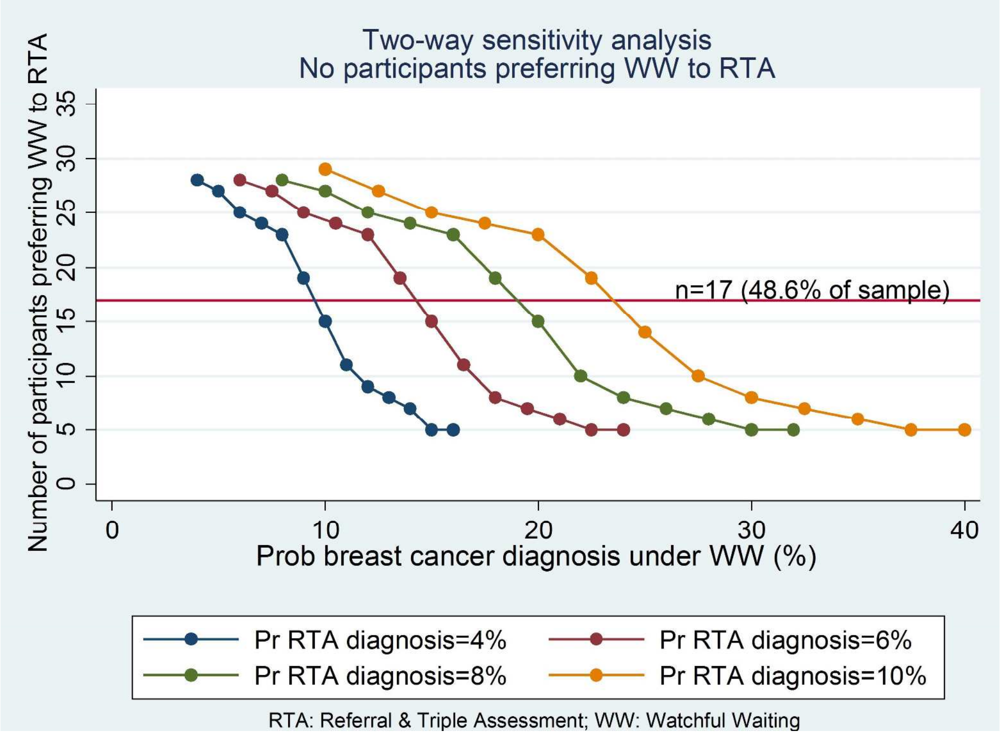

276x201mm (300 x 300 DPI)

5/5/2015

HRB Centre for Primary Care Research

Welcome to the Breast Cancer Preference Study

Please enter your participant number below

Start Study

HRB Centre for Primary Care Research

Please complete the paper based questionnaire before proceeding

Completed

5/5/2015

HRB Centre for Primary Care Research

This is a study aimed at understanding womens' perceptions of breast cancer. Women with certain risk factors, signs and symptoms are more likely than others to develop breast cancer.

We will present you with several health scenarios, and ask you to choose between different states of health.

Next

Scenario

You have presented to your GP with symptom(s) of breast cancer.
Your GP will take a history of your symptoms, and perform a breast examination. They may also perform any other examinations/tests that they feel are necessary (listen to your heart, take blood, etc.).
Based on history and examination findings, your GP will either recommend a "watchful waiting" programme, or triple assessment at a specialist breast clinic. However, it is your decision which option you prefer.

Definitions

Beside you are definitions for terms that you will come across in the scenarios. Please read them now, but you will also have a copy of them beside you to refer to at any point. Any word in **blue** in the scenarios has a definition provided.

5/5/2015

How will we measure your preferences?

We will use 2 methods (the VAS and standard gamble) to assess how you feel about each of the 11 health states. The health states will be presented to you in random order.
An explanation of each of the methods will follow. You also have laminated cards in front of you with the same information.
You can refer to these at any time that you wish.

Visual Analogue Scale - VAS

The Visual Analogue Scale is a scale from 0-100, with 0 being the worst health scenario you can think of (death), and 100 being the best health scenario (perfect health).
We will ask you to read each health care scenario individually, and then to place it where you think it fits along the scale.
You do not need even gaps between scenarios on the scale, and you can place two or more scenarios on the same number if you wish.
Scale: 0-100

Please include VAS scale here

36 people. Scen 1-5 Type Asse. 6-11 Watch Wil

Standard Gamble.

You will be presented with 11 different health state scenarios, each scenario represents your current permanent health state. We will ask you to make a gamble between your current health state with two other health states (perfect health or death). I want you to imagine that this carries a certain chance of you living the rest of your life in perfect health and a certain chance of you dying painlessly tomorrow.

It is important to remember your current health scenario, and your current symptoms, treatments, side-effects and prognosis. You will be given different chances of perfect health versus death, against the current scenario, and keep gambling until you reach a point where you cannot choose between the 2 options.

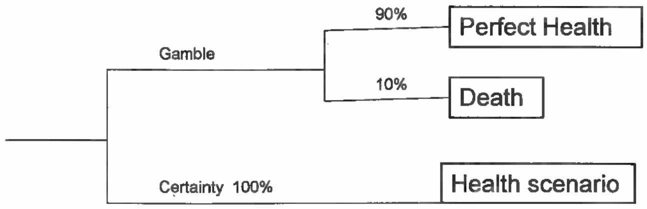

5/5/2015

Example- Standard Gamble

Imagine you have a heart condition. You have a choice between taking medication which has unpleasant side effects or taking no medication.

If you had to choose between taking this medication with side-effects for the rest of your life, or taking no medication and have perfect health, but with the risk of dying, what risk of death would you take to avoid the medication and side effects?

Another way of putting it is - what risk of dying painlessly tomorrow would you be willing to accept (if you didn't take the drugs) before you change your mind and decide you would rather take them?

•If offered a 90% chance of perfect health, no drugs, no side effects, but with a 10% risk of instant death, would you take it?

•If offered a 70% chance of perfect health, no drugs, no side effects, but with a 30% risk of instant death, would you take it?

Below, you will see 2 buttons. When you click on the right button, the chances of perfect health and death vary, these are examples to help you understand. In the text box below, please enter in the risk of death that you are willing to take.

Live in current Health State.	Versus	50% chance of perfect health and 50% chance of death.

[ ] % chance of death

(handwritten: no drugs, no side effects; instant; Enter)

The Study

We are going to present you with 11 possible health scenarios in a random order - please follow the instructions and if you have any questions please ask.

Definitions have been provided for any word or phrase in blue.

5/5/2015

1.0069

Health State 1: Referred to breast clinic for triple assessment - Stage 1 Breast Cancer

What it means:
You were referred for **triple assessment** and breast cancer has been diagnosed that is confined to the breast and may involve a small number of **lymph nodes**. This is an early stage of breast cancer.

Physical Consequences:
- You have undergone **triple assessment.**
- You will have surgery (**lumpectomy, mastectomy, lymph node removal**).
- **Chemotherapy** and/or **radiation therapy** to prevent the cancer from coming back.
- **Hormonal therapy** or **biological therapy** may be given, depending on the type of cancer cell.

Emotional Consequences:
- Fear over investigations and treatments (pain, side-effects).
- Anxiety or depression associated with diagnosis.
- Worry that cancer may spread or will not be fully cured.

VAS Scale

Assume that you are currently experiencing the health state you just read. On a scale of 0 to 100 where 0 is death and 100 perfect health where would you rate this health state. Please use the scale below.
When you are happy with your selection please click 'Completed'.

Health State 1: Referred to breast clinic for triple assessment - Stage 1 Breast Cancer

What it means:
You were referred for **triple assessment** and breast cancer has been diagnosed that is confined to the breast and may involve a small number of **lymph nodes**. This is an early stage of breast cancer.

Physical Consequences:
- You have undergone **triple assessment.**
- You will have surgery (**lumpectomy, mastectomy, lymph node removal**).
- **Chemotherapy** and/or **radiation therapy** to prevent the cancer from coming back.
- **Hormonal therapy** or **biological therapy** may be given, depending on the type of cancer cell.

Emotional Consequences:
- Fear over investigations and treatments (pain, side-effects).
- Anxiety or depression associated with diagnosis.
- Worry that cancer may spread or will not be fully cured.

Standard Gamble

You have the opportunity to gamble your current health state (as described above) for perfect health, but you also risk death if you take the gamble. It is important to remember about your current health scenario, and your current symptoms, treatments, side-effects and prognosis. Below, you will see 2 buttons. When you click on the right button, the chances of perfect health and death vary, these are examples to help you understand. In the text box below, please enter in the risk of death that you are willing to take in order to avoid this health state.

type

5/5/2015

Health State 2: Referred to breast clinic for triple assessment - Stage 2 Breast Cancer

What it means:
Triple assessment has found cancer that is contained within the breast. It may also be in surrounding **lymph nodes**.

Physical Consequences:
- You have undergone **triple assessment.**
- You will have surgery (**lumpectomy, mastectomy, lymph node removal**).
- You will have **radiation therapy.**
- You may have **chemotherapy** and/or **hormonal therapy,** depending on the type of cancer.

Emotional Consequences:
- Fear over investigations and treatments (pain, side-effects).
- Anxiety or depression associated with diagnosis.
- Fear of recurrence.

VAS Scale
Assume that you are currently experiencing the health state you just read. On a scale of 0 to 100 where 0 is death and 100 perfect health where would you rate this health state. Please use the scale below. When you are happy with your selection please click 'Completed'.

Health State 2: Referred to breast clinic for triple assessment; Stage 2 Breast Cancer

What it means:
Triple assessment has found cancer that is contained within the breast. It may also be in surrounding **lymph nodes**.

Physical Consequences:
- You have undergone **triple assessment.**
- You will have surgery (**lumpectomy, mastectomy, lymph node removal**).
- You will have **radiation therapy.**
- You may have **chemotherapy** and/or **hormonal therapy**, depending on the type of cancer.

Emotional Consequences:
- Fear over investigations and treatments (pain, side-effects).
- Anxiety or depression associated with diagnosis.
- Fear of recurrence.

Standard Gamble
You have the opportunity to gamble your current health state (as described above) for perfect health, but you also risk death if you take the gamble. It is important to remember about your current health scenario, and your current symptoms, treatments, side-effects and prognosis. Below, you will see 2 buttons. When you click on the right button, the chances of perfect health and death vary, these are examples to help you understand. In the text box below, please enter in the risk of death that you are willing to take in order to avoid this health state.

5/5/2015

Health State 3: Referred to breast clinic for triple assessment - Stage 3 Cancer

What it means:
You were referred for **triple assessment** and cancer was found in the breast, it has spread into surrounding tissues and many **lymph nodes** are involved.

Physical consequences:
- You have undergone **triple assessment.**
- You will have a surgery (**lumpectomy, mastectomy, lymph node removal**).
- You will probably have **radiation therapy** and/or **chemotherapy.**
- You may have **hormonal** or **biological therapy**, depending on the type of cancer cells.

Emotional consequences:
- Fear over investigations and treatments (pain, side-effects).
- Poorer prognosis, worry about survival.
- Fear of recurrence.

VAS Scale
Assume that you are currently experiencing the health state you just read. On a scale of 0 to 100 where 0 is death and 100 perfect health where would you rate this health state. Please use the scale below.
When you are happy with your selection please click 'Completed'.

Health State 3: Referred to breast clinic for triple assessment - Stage 3 Cancer

What it means:
You were referred for **triple assessment** and cancer was found in the breast, it has spread into surrounding tissues and many **lymph nodes** are involved.

Physical consequences:
- You have undergone **triple assessment.**
- You will have a surgery (**lumpectomy, mastectomy, lymph node removal**).
- You will probably have **radiation therapy** and/or **chemotherapy.**
- You may have **hormonal** or **biological therapy**, depending on the type of cancer cells.

Emotional consequences:
- Fear over investigations and treatments (pain, side-effects).
- Poorer prognosis, worry about survival .
- Fear of recurrence.

Standard Gamble
You have the opportunity to gamble your current health state (as described above) for perfect health, but you also risk death if you take the gamble. It is important to remember about your current health scenario, and your current symptoms, treatments, side-effects and prognosis. Below, you will see 2 buttons. When you click on the right button, the chances of perfect health and death vary, these are examples to help you understand. In the text box below, please enter in the risk of death that you are willing to take in order to avoid this health state.

5/5/2015

Health State 4: Referred to breast clinic for triple assessment - Stage 4 Breast Cancer found

What it means:
You are referred for **triple assessment**, and cancer is found in your breast, which has also spread to one or more organs in the body. At this stage the cancer cannot be cured, but treatment can slow down the disease and let you live longer.

Physical consequences:
- You have undergone **triple assessment**.
- The main treatment is **chemotherapy.**
- You may have surgery or **radiation therapy** to treat pain and other symptoms.
- Possible **hormonal and/or biological therapy**, depending on type of cancer.

Emotional consequences:
- Fear due to poor prognosis and treatments.
- Fear of dying.

VAS Scale
Assume that you are currently experiencing the health state you just read. On a scale of 0 to 100 where 0 is death and 100 perfect health where would you rate this health state. Please use the scale below. When you are happy with your selection please click 'Completed'.

Health State 4: Referred to breast clinic for triple assessment - Stage 4 Breast Cancer found

What it means:
You are referred for **triple assessment**, and cancer is found in your breast, which has also spread to one or more organs in the body. At this stage the cancer cannot be cured, but treatment can slow down the disease and let you live longer.

Physical consequences:
- You have undergone **triple assessment**.
- The main treatment is **chemotherapy.**
- You may have surgery or **radiation therapy** to treat pain and other symptoms.
- Possible **hormonal and/or biological therapy**, depending on type of cancer.

Emotional consequences:
- Fear due to poor prognosis and treatments.
- Fear of dying.

Standard Gamble
You have the opportunity to gamble your current health state (as described above) for perfect health, but you also risk death if you take the gamble. It is important to remember about your current health scenario, and your current symptoms, treatments, side-effects and prognosis. Below, you will see 2 buttons. When you click on the right button, the chances of perfect health and death vary, these are examples to help you understand. In the text box below, please enter in the risk of death that you are willing to take in order to avoid this health state.

5/5/2015

Health State 5: Referred to breast clinic for triple assessment - No cancer found

What it means:
You were referred for **triple assessment** and no cancer was found.

Physical consequences:
•You have undergone **triple assessment**.

Emotional consequences:
• Fear of investigations.
• Fear that the results were wrong and that you could still have cancer.

VAS Scale
Assume that you are currently experiencing the health state you just read. On a scale of 0 to 100 where 0 is death and 100 perfect health where would you rate this health state. Please use the scale below.
When you are happy with your selection please click 'Completed'.

Health State 5: Referred to breast clinic for triple assessment - No cancer found

What it means:
You were referred for **triple assessment** and no cancer was found.

Physical consequences:
•You have undergone **triple assessment**.

Emotional consequences:
•Fear of investigations
•Fear that the results might be wrong and that you could still have cancer.

Standard Gamble
You have the opportunity to gamble your current health state (as described above) for perfect health, but you also risk death if you take the gamble. It is important to remember about your current health scenario, and your current symptoms, treatments, side-effects and prognosis. Below, you will see 2 buttons. When you click on the right button, the chances of perfect health and death vary, these are examples to help you understand. In the text box below, please enter in the risk of death that you are willing to take in order to avoid this health state.

5/5/2015

Health State 6: Watchful waiting, recurrence/worsening of symptoms, referred to breast clinic for triple assessment - Stage 1 Breast Cancer found

What it means
After a period of **watchful waiting** with your GP, your symptoms were not resolving/becoming worse. You were referred for **triple assessment** and breast cancer has been diagnosed that is confined to the breast and may involve a small number of **lymph nodes**. This is an early stage of breast cancer.

Physical consequences:
- You have undergone **triple assessment**.
- You will have surgery (**lumpectomy, mastectomy, lymph node removal**).
- **Chemotherapy** and/or **radiation therapy** to prevent the cancer from coming back.
- **Hormonal therapy** or **biological therapy** may be given, depending on the type of cancer cells.

Emotional Consequences:
- Anger/worry about delay in diagnosis.
- Fear over investigations and treatments (pain, side-effects).
- Anxiety or depression associated with diagnosis.
- Worry that cancer may spread or will not be fully cured.

VAS Scale
Assume that you are currently experiencing the health state you just read. On a scale of 0 to 100 where 0 is death and 100 perfect health where would you rate this health state. Please use the scale below. When you are happy with your selection please click 'Completed

Health State 6: Watchful waiting, recurrence/worsening of symptoms, referred to breast clinic for triple assessment - Stage 1 Breast Cancer found

What it means
After a period of **watchful waiting** with your GP, your symptoms were not resolving/becoming worse. You were referred for **triple assessment** and breast cancer has been diagnosed that is confined to the breast and may involve a small number of **lymph nodes**. This is an early stage of breast cancer.

Physical consequences:
- You have undergone **triple assessment**.
- You will have surgery (**lumpectomy, mastectomy, lymph node removal**).
- **Chemotherapy** and/or **radiation therapy** to prevent the cancer from coming back.
- **Hormonal therapy** or **biological therapy** may be given, depending on the type of cancer cells.

Emotional Consequences:
- Anger/worry about delay in diagnosis.
- Fear over investigations and treatments (pain, side-effects).
- Anxiety or depression associated with diagnosis.
- Worry that cancer may spread or will not be fully cured

Standard Gamble
You have the opportunity to gamble your current health state (as described above) for perfect health, but you also risk death if you take the gamble. It is important to remember about your current health scenario, and your current symptoms, treatments, side-effects and prognosis. Below, you will see 2 buttons. When you click on the right button, the chances of perfect health and death vary, these are examples to help you understand. In the text box below, please enter in the risk of death that you are willing to take in order to avoid this health state.

5/5/2015

Health State 7: Watchful waiting, recurrence/worsening of symptoms, referred to breast clinic for triple assessment - Stage 2 Breast Cancer found

What it means:
After a period of **watchful waiting**, your symptoms were not resolving/became worse. You were referred for **triple assessment**, and cancer was found, which is contained within the breast. It may also be in surrounding **lymph nodes.**

Physical consequences:
- You have undergone **triple assessment**.
- You will have surgery (**lumpectomy, mastectomy, lymph node removal**).
- You will have **radiation therapy.**
- You may have **chemotherapy**, and/or **hormonal therapy,** depending on the type of cancer

Emotional consequences:
- Anger/worry about delay in diagnosis.
- Fear over investigations and treatments (pain, side-effects).
- Anxiety or depression due to cancer diagnosis.
- Fear of recurrence.

VAS Scale
Assume that you are currently experiencing the health state you just read. On a scale of 0 to 100 where 0 is death and 100 perfect health where would you rate this health state. Please use the scale below.
When you are happy with your selection please click 'Completed

Health State 7: Watchful waiting, recurrence/worsening of symptoms, referred to breast clinic for triple assessment - Stage 2 Breast Cancer found

What it means:
After a period of **watchful waiting**, your symptoms were not resolving/became worse. You were referred for **triple assessment**, and cancer was found, which is contained within the breast. It may also be in surrounding **lymph nodes.**

Physical consequences:
- You have undergone **triple assessment**.
- You will have surgery (**lumpectomy, mastectomy, lymph node removal**).
- You will have **radiation therapy.**
- You may have **chemotherapy**, and/or **hormonal therapy,** depending on the type of cancer.

Emotional consequences:
- Anger/worry about delay in diagnosis.
- Fear over investigations and treatments (pain, side-effects).
- Anxiety or depression due to cancer diagnosis.
- Fear of recurrence.

Standard Gamble
You have the opportunity to gamble your current health state (as described above) for perfect health, but you also risk death if you take the gamble. It is important to remember about your current health scenario, and your current symptoms, treatments, side-effects and prognosis. Below, you will see 2 buttons. When you click on the right button, the chances of perfect health and death vary, these are examples to help you understand. In the text box below, please enter in the risk of death that you are willing to take in order to avoid this health state.

5/5/2015

Health State 8: Watchful waiting, recurrence/worsening of symptoms, referred to breast clinic for triple assessment - Stage 3 Breast Cancer found

What it means:
After a period of **watchful waiting**, your symptoms have not resolved/become worse. You are referred for **triple assessment**. Cancer is found in the breast, it has spread into surrounding tissues, and many **lymph nodes** are involved.

Physical consequences:
- You have undergone **triple assessment.**
- You will have a surgery (**lumpectomy, mastectomy, lymph node removal**).
- You will probably have **radiation therapy** and/or **chemotherapy.**
- You may have **hormonal** or **biological therapy**, depending on the type of cancer cells.

Emotional consequences:
- Anger/worry about delay in diagnosis.
- Fear over investigations and treatments (pain, side-effects).
- Poorer prognosis, worry about survival.
- Fear of recurrence.

VAS Scale
Assume that you are currently experiencing the health state you just read. On a scale of 0 to 100 where 0 is death and 100 perfect health where would you rate this health state. Please use the scale below. When you are happy with your selection please click 'Completed

Health State 8: Watchful waiting, recurrence/worsening of symptoms, referred to breast clinic for triple assessment - Stage 3 Breast Cancer found

What it means:
After a period of **watchful waiting**, your symptoms have not resolved/become worse. You are referred for **Triple assessment**. Cancer is found in the breast, it has spread into surrounding tissues, and many **lymph nodes** are involved.

Physical consequences:
- You have undergone **triple assessment.**
- You will have a surgery (**lumpectomy, mastectomy, lymph node removal**).
- You will probably have **radiation therapy** and/or **chemotherapy.**
- You may have **hormonal** or **biological therapy**, depending on the type of cancer cells.

Emotional consequences:
- Anger/worry about delay in diagnosis.
- Fear over investigations and treatments (pain, side-effects).
- Poorer prognosis, worry about survival.
- Fear of recurrence.

Standard Gamble
You have the opportunity to gamble your current health state (as described above) for perfect health, but you also risk death if you take the gamble. It is important to remember about your current health scenario, and your current symptoms, treatments, side-effects and prognosis. Below, you will see 2 buttons. When you click on the right button, the chances of perfect health and death vary, these are examples to help you understand. In the text box below, please enter in the risk of death that you are willing to take in order to avoid this health state.

12

Health State 9: Watchful waiting, recurrence/worsening of symptoms, referred to breast clinic for triple assessment - Stage 4 Breast Cancer found

What it means:
After a period of **watchful waiting** with your GP, your symptoms have not resolved/became worse. You are referred for **triple assessment**, and cancer is found in your breast, which has also spread to one or more organs in the body. At this stage the cancer cannot be cured, but treatment can slow down the disease and let you live longer.

Physical consequences:
- You have undergone **triple assessment**.
- The main treatment is **chemotherapy**.
- You may have surgery or **radiation therapy** to treat pain and other symptoms.
- Possible **hormonal** and/or **biological therapy**, depending on type of cancer.

Emotional Consequences:
- Anger/worry about delay in diagnosis.
- Fear due to poor prognosis and treatments.
- Fear of dying.

VAS Scale
Assume that you are currently experiencing the health state you just read. On a scale of 0 to 100 where 0 is death and 100 perfect health where would you rate this health state. Please use the scale below. When you are happy with your selection please click 'Completed'.

Health State 1
0 ___|____________________________ 100

Health State 9: Watchful waiting, recurrence/worsening of symptoms, referred to breast clinic for triple assessment - Stage 4 Breast Cancer found

What it means:
After a period of **watchful waiting** with your GP, your symptoms have not resolved/became worse. You are referred for **triple assessment**, and cancer is found in your breast, which has also spread to one or more organs in the body. At this stage the cancer cannot be cured, but treatment can slow down the disease and let you live longer.

Physical consequences:
- You have undergone **triple assessment**.
- The main treatment is **chemotherapy**.
- You may have surgery or **radiation therapy** to treat pain and other symptoms.
- Possible **hormonal** and/or **biological therapy**, depending on type of cancer.

Emotional Consequences:
- Anger/worry about delay in diagnosis.
- Fear due to poor prognosis and treatments.
- Fear of dying.

Standard Gamble.
You have the opportunity to gamble your current health state (as described above) for perfect health, but you also risk death if you take the gamble. It is important to remember about your current health scenario, and your current symptoms, treatments, side-effects and prognosis. Below, you will see 2 examples. When you click on the right button, the chances of perfect health and death vary, these are examples to help you understand. In the text box below, please enter in the risk of death that you are prepared to take in order to avoid this health state.

5/5/2015

Health State 10: Watchful waiting, referred to breast clinic for triple assessment - No cancer found

What it means
After a period **watchful waiting** with your GP. Your symptoms were not resolving/became worse. You are referred for **triple assessment** and no cancer was found.

Physical Consequences:
- You have undergone **triple assessment.**

Emotional Consequences:
- Fear of investigations.
- Worry about the delay in diagnosis.
- Fear the results might be wrong and that you still could have cancer.

VAS Scale
Assume that you are currently experiencing the health state you just read. On a scale of 0 to 100 where 0 is death and 100 perfect health where would you rate this health state. Please use the scale below.
When you are happy with your selection please click 'Completed'.

Health State 10 : Watchful waiting, referred to breast clinic for triple assessment - No cancer found

What it means
After a period **watchful waiting** with your GP. Your symptoms were not resolving/became worse. You are referred for **triple assessment** and no cancer was found.

Physical Consequences:
- You have undergone **triple assessment.**

Emotional Consequences:
- Fear of investigations.
- Worry about the delay in diagnosis.
- Fear the results may be wrong and that you still could have cancer.

Standard Gamble.
You have the opportunity to gamble your current health state (as described above) for perfect health, but you also risk death if you take the gamble. It is important to remember about your current health scenario, and your current symptoms, treatments, side-effects and prognosis. Below, you will see 2 buttons. When you click on the right button, the chances of perfect health and death vary, these are examples to help you understand. In the text box below, please enter in the risk of death that you are willing to take in order to avoid this health state.

For peer review only - http://bmjopen.bmj.com/site/about/guidelines.xhtml

5/5/2015

Health State 11: Watchful waiting, resolution of symptoms, no referral needed.

What it means:
After a period of **watchful waiting**, symptoms have stopped or are resolving within 3 month's follow up with GP. You do not need further investigation.

Physical Consequences:
•.No further tests are needed.

Emotional Consequences:
• Relief that symptoms are not serious.
• Worry that there might be a cancer that has been missed.

VAS Scale
Assume that you are currently experiencing the health state you just read. On a scale of 0 to 100 where 0 is death and 100 perfect health where would you rate this health state. Please use the scale below. When you are happy with your selection please click 'Completed'.

Health State 11: Watchful waiting, resolution of symptoms, no referral needed.

What it means:
After a period of **watchful waiting**, symptoms have stopped or are resolving within 3 month's follow up with GP. You do not need further investigation.

Physical Consequences:
•.No further tests are needed.

Emotional Consequences:
• Relief that symptoms are not serious.
• Worry that there might be a cancer that has been missed.

Standard Gamble.

You have the opportunity to gamble your current health state (as described above) for perfect health, but you also risk death if you take the gamble. It is important to remember about your current health scenario, and your current symptoms, treatments, side-effects and prognosis. Below, you will see 2 buttons. When you click on the right button, the chances of perfect health and death vary, these are examples to help you understand. In the text box below, please enter in the risk of death that you are willing to take in order to avoid this health state.

STROBE Statement—checklist of items that should be included in reports of observational studies

	Item No	Recommendation
**Title and abstract**	1	Decision analysis mentioned in title and abstract
		(*b*) Provide in the abstract an informative and balanced summary of what was done and what was found- see abstract
**Introduction**		
Background/rationale	2	Explain the scientific background and rationale for the investigation being reported
Objectives	3	State specific objectives, including any prespecified hypotheses See pages 4-8
**Methods**		
Study design	4	Present key elements of study design early in the paper- page 4-8
Setting	5	Describe the setting, locations, and relevant dates, including periods of recruitment, exposure, follow-up, and data collection- page 4-8
Participants	6	Page 4-8
Variables	7	Details of decision tree and utility states in methods pages 4-8 and in supplementary file of health states computerized programme that performed utility assessment
Data sources/ measurement	8*	Table 1
Bias	9	Limitations discussed page 11
Study size	10	Explanation re: feasibility study in methods section page 7
Quantitative variables	11	Explanation, methods page 6-8
Statistical methods	12	Plan of analysis pages 6-8; includes sensitivity analysis

Continued on next page

For peer review only

**Results**		
Participants	13*	Page 9
Descriptive data	14*	Table 2
Main results	16	Page 9, Table 3, Figure 2
**Discussion**		
Key results	18	Page 10-11
Limitations	19	Page 11
Interpretation	20	Page 11-12
Generalisability	21	Page 11-12
**Other information**		
Funding	22	Page 13 Give the source of funding and the role of the funders for the present study and, if applicable, for the original study on which the present article is based

For peer review only

For peer review only - http://bmjopen.bmj.com/site/about/guidelines.xhtml

Quantifying patient preferences for symptomatic breast clinic referral: a decision analysis study

Aisling Quinlan[1], BSc, Kirsty K O'Brien[1], PhD, Rose Galvin[1,3], PhD, Colin Hardy[1], MSc, Ronan McDonnell[1], PhD, Doireann Joyce[2], MB BCh BAO, Ronald D.McDowell[1], PhD, Emma Aherne[1],MB BCh BAO, Claire Keogh[1], PhD, Katriona O'Sullivan[1,4], PhD, Tom Fahey[1], MD

[1]HRB Centre for Primary Care Research, Department of General Practice, Royal College of Surgeons in Ireland (RCSI), 123 St.Stephens Green, Dublin 2, Republic of Ireland.

[2]National University of Ireland Galway, University Road, Galway. Republic of Ireland.

[3]Department of Clinical Therapies, University of Limerick, Limerick.

[4]School of Computer Science and Statistics, Trinity College Dublin.

*Corresponding Author

Tom Fahey, HRB Centre for Primary Care Research, Department of General Practice, Royal College of Surgeons in Ireland, 123 St. Stephen's Green, Dublin 2, Republic of Ireland.

Email: tomfahey@rcsi.ie

Word count:

For peer review only

Abstract

Objectives: Decision analysis study that incorporates patient preferences and probability estimates Tto investigate the impact of women's preferences for referral or an alternative strategy of watchful waiting if faced with symptoms that could be due to breast cancer.

Setting: Community-based study.

Participants: Asymptomatic women aged 30-60 years.

Interventions: Participants were presented with 11 health scenarios that represent the possible consequences of symptomatic breast problems. Participants were asked the risk of death that they were willing to take in order to avoid the health scenario using the standard gamble (SG) utility method. This process was repeated for all 11 health scenarios. Formal decision analysis for the preferred individual decision was then estimated for each participant.

Primary outcome measure: The preferred diagnostic strategy, either watchful waiting or referral to a breast clinic. Sensitivity analysis was used to examine how each varied according to changes in the probabilities of the health scenarios.

Results: A total of 35 participants completed the interviews, with median age 41 years (Interquartile range 35 to 47 years). The majority of the study sample were employed (n=32, 91.4%), with a third-level education (n=32, 91.4%) and with knowledge of someone with breast cancer (n=30, 85.7%). When individual preferences were accounted for, 25 (71.4%) patients preferred watchful waiting to referral for triple assessment as their preferred initial diagnostic strategy. Sensitivity analysis shows that referral for triple assessment becomes the dominant strategy at the upper probability estimate (18%) of breast cancer in the community.

Conclusions: Watchful waiting is an acceptable strategy for most women who present to their GP with breast symptoms. These findings suggest that current referral guidelines should take more explicit account of women's preferences in relation in terms of the initial diagnostic strategy for symptomatic breast problems.

For peer review only - http://bmjopen.bmj.com/site/about/guidelines.xhtml

Strengths and limitations of this study

Strength of this study is that it accounts for women's preferences in relation to a watchful waiting or immediate referral strategy when faced with symptoms that could be due to breast cancer.

Weakness is that preferences were elicited in asymptomatic women. Women with symptoms may express different preferences.

The impact of eliciting preferences should be done in the context of a randomized trial of a decision aid that uses decision analysis as a form of shared decision making for women with symptoms that could be due to breast cancer.

Taking account of women's preferences might mean that ~~far~~ significantly fewer women would be referred to symptomatic breast clinics with a consequent saving in investigations and costs, and reducing the burden of "medicalization" in women with benign breast disease.

For peer review only - http://bmjopen.bmj.com/site/about/guidelines.xhtml

Introduction

Between 2009-2011, it was reported that 31% of cancers in Irish women were due to breast cancer, making it the most prevalent cancer in Irish women [1]. In response, the National Cancer Control Programme (NCCP) clinical guidelines were introduced to streamline the referral process to symptomatic breast units. At present, GPs act as gatekeepers for referral of women with breast symptoms. Referral to symptomatic breast clinics results in triple assessment: a three step process comprising of clinical, radiological and histological examination after fine needle aspiration [2]. Over the past ten years, the number of women referred to symptomatic breast clinics in Ireland has significantly increased, despite the fact there has been no rise in breast cancer incidence [2]. ~~For example i~~In 2006, there were 23,575 new referrals with 2137 breast cancers diagnosed, ~~increasing~~ with~~to~~ 32,249 referalls and 1879 breast cancers diagnosed in 2009 [3],and 37,631 new referalls in 2010 with 2012 new breast cancers diagnosed [4]. ~~Out of the 37,631 referrals in 2010, only 2,012 were diagnosed with breast cancer~~Consequently the malignant;benign ratio has altered from 1:10 in 2006, to 1:16 in 2009 to 1:18 in 2010 [4]. The rise in these referrals for triple assessment without an increase in breast cancer diagnosis ~~indicates~~ means that more women are being exposed to invasive diagnostic investigations.

Clinical prediction rules (CPRs) are clinical tools that quantify the independent impact of factors from a patients history, physical examination and diagnostic tests and stratify patients according to the probability of having a target disorder [5]. Recently, CPRs have been derived and validated to calculate a patient's risk of breast cancer [6, 7], and have been proposed as methods to discriminate between patients at high risk of breast cancer from low risk patients. Such an approach may serve to decrease the number of unnecessary referrals to symptomatic breast clinics in women with a low probability of breast cancer. Patients who are considered low risk could, as an alternative, undergo a watchful waiting strategy (up to 6-8 weeks) with their GP. However, it is unclear from the current literature if patients who are considered low risk would find this acceptable.

Decision analysis is a technique to aid decision making when uncertainty exists over the balance between benefits and risks of treatment [8]. Decision trees have been utilised to assess how

For peer review only

For peer review only - http://bmjopen.bmj.com/site/about/guidelines.xhtml

patient's preferences impact on diagnosis and treatment for conditions such as ovarian cancer [9] and atrial fibrillation [10]. To date, little is known about how women who have symptomatic breast problems feel about the options of immediate referral or watching waiting in terms of referral to a symptomatic breast clinic. The aim of this study is assess the impact of patient preferences for immediate referral to a specialist breast clinic or watchful waiting in primary care. We developed a decision tree and elicited patients preferences for these alternative diagnostic strategies by combining individual preferences with probability estimates of breast cancer using decision analysis.

Methodology

Study design

This study utilized decision analysis in the form of utility assessment and folding back a decision tree. The STROBE standardized reporting guidelines were followed to ensure the standardized conduct and reporting of the research. Ethical approval was received from the Research Ethics Committee at the Royal College of Surgeons in Ireland.

Developing the decision tree and health scenarios

Literature searches were conducted in medical databases such as PubMed, the Cochrane library and Web of Science to assist with the development of the decision tree and health scenarios. A specialist registrar with clinical experience in a symptomatic breast clinic was also consulted during this development phase. Following these consultations, the decision tree was constructed to portray the temporal course of the two diagnostic options (Figure 1). The decision tree had one decision node with two diagnostic branches (a choice between watchful waiting or immediate referral), nine probability or chance nodes and eleven health outcome nodes. In accordance with the literature the tree was designed such that only two branches emanated from each chance node [11]. The number of outcome nodes differed between the two branches of the tree in order to accommodate the additional step (an observation period before breast cancer assessment among patients who return to their GP) in the watchful

For peer review only - http://bmjopen.bmj.com/site/about/guidelines.xhtml

waiting branch of the tree. It is not always possible or appropriate for each branch of a decision tree to have the same number of outcome nodes as this will be dependent on the nature and temporal order of events in that branch [12]. The decision tree is "balanced" as each branch has advantages and disadvantages and hence it is not possible to readily identify a treatment path that is ideal in all respects [11].

A total of eleven health scenarios were constructed, one for each outcome node. For this study the standard gamble (SG) method of utility assessment was used [13] [14]. Briefly, participants presented with a description of each health state, its physical consequences and emotional consequences, and were given the opportunity to remain in the health state or to gamble it for perfect health, but with the risk of death if they took the gamble. Participants were presented with differing probabilities showing how the chance of perfect health and death varied with each other for each health state (the "ping-pong method"). The utility value was one minus the risk of death they were willing to take in order to avoid the health state described. The 11 health states were presented in a random order. Details of the health states and the standard gamble scenarios seen by the participants are provided in the supplementary materials section.

Probability estimates for each chance node were obtained from the medical literature [7,15-28]. Where possible, relevant probability estimates for each chance node were combined using random effects meta-analysis (Table 1). The same probabilities were used for each participant. It was not possible to estimate from the literature the probability of a diagnosis of breast cancer among patients who returned to their doctor following a period of watchful waiting. Clinical experience suggested this would be several percentage points higher in absolute terms than the probability of a diagnosis of breast cancer following RTA and an estimate of 10% was used.

For peer review only - http://bmjopen.bmj.com/site/about/guidelines.xhtml

Participants and setting

Interviews were conducted over a six month period in Dublin from October 2014 to April 2015. Asymptomatic women aged 30-60 years were invited to participate in the study. Participants were recruited using staff notice boards, social media and snowball sampling. The majority of interviews were carried out in a meeting room in the research building. Some interviews were conducted in peoples' place of work as this was more convenient for them. Written informed consent was obtained from all participants prior to participation. The interviews were conducted with a researcher present, who was available to answer any questions and to obtain feedback about the interview. Prior to the interview, participants were given a baseline demographics questionnaire and a multiple-choice questionnaire to assess their knowledge of breast cancer. As this is a feasibility study for a proposed randomized controlled trial, no formal sample size was calculated.

Utility assessment

A computer-based assessment programme which presented and recorded participant responses was designed. This programme incorporated an introduction, an explanation of the interview process, and presentation of each clinical scenario in random order using the standard gamble method of utility assessment. An example of the standard gamble was included for orientation and definitions were provided for any medical terminology used. The purpose of using a computer programme was to allow the interviewer to limit their interaction with each participant, standardize utility assessment and minimize interviewer bias. A pilot study was carried out and modifications made to the computer programme and utility assessment process. See supplementary table of health scenarios.

Ethical approval and data Protection

The data from the computer programme was stored with a participant ID only and saved to an encrypted password protected desktop computer. To protect patient identity, a MS excel spreadsheet was created; containing just patient names and identification numbers, and this

For peer review only - http://bmjopen.bmj.com/site/about/guidelines.xhtml

was stored in a secured, password protected folder on the server and kept separate to the data from the interview. The study was approved by the RCSI institutional ethics committee.

Decision analysis

The utility for each outcome, “a number assigned to the quality of life a patient would attach to a particular outcome on a defined scale “ [12], was calculated as 1-the risk of death (expressed as a fraction) a participant was willing to take in order to avoid the health scenario as described. For each participant, an expected utility score for each diagnostic option was obtained by multiplying and summing the relevant probabilities and utilities. The branch which had the highest expected utility score was determined to be the preferred diagnostic strategy- watchful waiting or referral for triple assessment.

Sensitivity analysis was used to determine the robustness of the conclusion drawn from the decision tree [29]. For our study each of the probability estimates derived from the literature was allowed to vary between the lower and upper limits of the 95% confidence interval for that estimate, in order to assess the extent to which the preferred diagnostic strategy changed. The difference between the number of patients preferring watchful waiting using the upper limit of the 95% confidence interval and the number of patients preferring watchful waiting using the lower limit of the 95% confidence interval for each probability estimate (the swing) was squared and totalled; the impact of varying each of the probability estimates was measured as a percentage of the total squared-swing. The sensitivity analyses accounted for the fact that some probability estimates were used more than once by incorporating the conditioning event into the definition of the variables under consideration [30]. An additional two-way sensitivity analysis explored how women’s preferred diagnostic option changed as the probability of diagnosis following watchful waiting varied relative to the probability of diagnosis following referral and triple assessment.

For peer review only - http://bmjopen.bmj.com/site/about/guidelines.xhtml

Standard descriptive statistics were used to summarise the sample. Results were held to be significant if they refer to statistical significance on a two-sided test evaluated at the 5% level. All analyses were performed using STATA 13 [31].

For peer review only

For peer review only - http://bmjopen.bmj.com/site/about/guidelines.xhtml

Results

A total of 36 women participated, with 35 interviews completed in full. The median age of participants was 41 years (interquartile range (IQR) 35 to 47 years). The demographic details of participants are described in Table 2. The majority of participants were employed (32, 91.4%), had a third-level education (32, 91.4%) and knew someone with breast cancer (30, 85.7%). Less than half the study sample (15, 42.9%) reported having a prior mammography. There was borderline evidence of a negative correlation between participants' self-reported knowledge of breast cancer and their actual knowledge based on responses to a multiple-choice questionnaire (p=0.06).

When patient preferences were taken into account, 25 patients (71.4%, 95%CI 19%, 30%) preferred watchful waiting to referral as the initial diagnostic strategy. Sensitivity analysis showed that the overall preferred diagnostic strategy changed to referral when the probability of breast cancer following a period of watchful waiting increased; only ten women favoured watchful waiting when the probability of breast cancer following a period of watchful waiting had increased to 18% (Table 3). Swings in the group preference according to changes in the probabilities associated with each of the stage of breast cancer were small in comparison (Table 3).

In terms of two-way sensitivity analysis more than half the participating women still preferred watchful waiting when the probability of a diagnosis of breast cancer following a period of watchful waiting was 2.25 times as high as the probability of diagnosis following referral and triple assessment. (Figure 2) This pattern remained consistent irrespective of the probability of breast cancer following referral and triple assessment, estimated at levels of 4%, 6%, 8% and 10%.

For peer review only - http://bmjopen.bmj.com/site/about/guidelines.xhtml

Discussion

Summary of main findings

This study shows that a strategy of watching waiting in women with breast symptoms in primary care should could be considered as an acceptable alternative to immediate referral to a symptomatic breast clinic, but this strategy of shared decision making using decision analysis should be tested in a randomized controlled trial. These Our observational findings suggest that women's own preferences should form an important element of the decision to refer to a symptomatic breast clinic and that these preferences should be accounted as part of a shared decision making process. Sensitivity analysis shows that as the probability of breast cancer increases, the diagnostic strategy of referral for triple assessment dominates. Two way sensitivity analysis shows that, across the likely probability range of breast cancer under a watchful waiting strategy, referral for triple assessment becomes the dominant strategy once the probability of breast cancer is two and a half times more likely than under a watchful waiting strategy.

Results in the context of the current literature

Although utility assessment has been used to evaluate the acceptability of various clinical states and treatment regimes in the breast cancer literature [32-34] it has not been used to examine the acceptability of watchful waiting in low risk patients presenting with signs and symptoms of possible breast cancer. A previous study by McCafferty et alstudy that assessed the effects of alternative terms for ductal carcinoma in-situ (DCIS). found that watchful waiting is acceptable to patients with low gradeprovided that research shows that watchful waiting is a safe and effective option ductal carcinoma in-situ (DCIS) but this depended on the terminology used for the disease [35]. These findings show thaThe preliminary findings from our study suggest thatt women may beare prepared to accept a conservative, non-invasive strategy of watchful waiting when the probability of breast cancer is low.

Watchful waiting has also been described as a key technique for more effective management of other cancers such as prostate cancer [36]. In prostate cancer the use of PSA testing has led to

For peer review only - http://bmjopen.bmj.com/site/about/guidelines.xhtml

an increase in the incidence of prostate cancer but not a decrease in the mortality rate, suggesting that PSA testing often identifies low risk cancers [37]. Given the serious and common side effects associated with treatment in prostate cancer some studies have shown that some men prefer watchful waiting to treatment options [38]. In our study the side effects of further diagnostic testing (triple assessment) are less onerous than treatment of localized prostate cancer, but many women would experience high levels of anxiety and stress upon referral to a symptomatic breast clinic. This study suggests that many women would find it more acceptable to undergo watchful waiting for a period rather than participate in diagnostic testing when their risk of breast cancer is low.

A qualitative study examining women's views on over-diagnosis in breast cancer screening showed that 50% of the population study felt they needed to make more careful personal decisions about screening and would consider watchful waiting as an alternative route if they were found to have a low risk cancer [39]. Like our study watchful waiting was found to be an acceptable strategy when the risk involved is low and the health outcomes are positive.

Strengths and limitations

This study has several strengths. We used an evidence-based approach, quantifying probabilities from the literature, and developing health states that incorporate the common features of breast cancer. We tested and modified the wording of the health states and the computer programme in a pilot group of participants and refined our protocol based on feedback from these participants. We tried to limit any bias caused by the interviewer by using a computer programme and providing the participant with written definitions and information. Examples of the standard gamble were given to the participants prior to the interview, so participants had the opportunity to familiarize themselves with the method. Although the sample was relatively small, it included participants with a range of ages and knowledge of breast cancer. We acknowledge that the sample of participants is not representative of the population at large; that women were asymptomatic and that preferences may change or be different when faced with possible symptoms of breast cancer- a breast lump or nipple change.

For peer review only - http://bmjopen.bmj.com/site/about/guidelines.xhtml

However, our results suggest that when preferences about a watchful waiting strategy is elicited, many women are happy to adopt this approach. Based on these provisional results, we hope to develop a decision aid for women with symptomatic breast problems and test it value in a randomized controlled trial.

Clinical and policy Implications

The current referral process leads to many patients who are at low risk of breast cancer being referred to busy symptomatic breast clinics, with a risk of iatrogenic harm, increased patient anxiety and over-medicalizing of a self-limiting condition. With more emphasis on the development of clinical prediction rules, it will be possible to identify and quantify each individual women's risk of breast cancer. A recent study derived a clinical prediction rule (CPR) for predicting the risk of breast cancer for women presenting to their GP with breast symptoms. [7] A total of 6,590 patients were included in the derivation study and 4.9% were diagnosed with breast cancer. Independent clinical predictors for breast cancer were: increasing age by year (adjusted OR 1.08, 95%CI 1.07-1.09); presence of a lump (5.63, 95%CI 4.2-7.56); nipple change (2.77, 95%CI 1.68-4.58) and nipple discharge (2.09, 95% CI 1.1-3.97). Validation of the rule (n=911) also shows that the probability of breast cancer is higher with an increasing number of these independent clinical variable. By incorporating patient preferences with an individual's probability of breast cancer from a CPR, a strategy of watchful waiting is acceptable, cost effective and likely to avoid considerable iatrogenic harm. Clinical practice guidelines should incorporate probability estimates of breast cancer based on CPRs and urge elicitation of patient preference for alternative diagnostic options- watchful waiting or referral. More formally, decision aids that could be developed in conjunction with the symptomatic breast CPRs to enable the alternative options of watchful waiting or referral based on individual preferences.

Conclusion

For peer review only - http://bmjopen.bmj.com/site/about/guidelines.xhtml

This study suggests that ~~W~~watchful waiting ~~appears~~ may~~to~~ be an acceptable diagnostic strategy for women who present to their general practitioner with breast symptoms. These findings should be validated in a larger and more comprehensive study of patient preferences in women with breast symptoms in a randomized controlled trial. Clinical practice guidelines should reflect the importance of eliciting women's preferences for watchful waiting as an alternative to referral to a symptomatic breast clinic, particularly in women with low risk clinical features.

Competing interests

The authors declare that they have no competing interests.

Authors' contributions

All authors were involved in the study conception and design. AQ, RG and KOB acquired data for analysis. RmcD designed the computer-based utility analysis programme. CH and RDMcD performed statistical analysis. AQ interpreted the data and drafted the paper. KOB, RG, RDMcD and TF critically revised the draft manuscript. All authors read and approved the final manuscript.

Funding statement

Health Research Board of Ireland grant number HRC-2007-1.

Data sharing statement

We are happy to share the data from this study

For peer review only

For peer review only - http://bmjopen.bmj.com/site/about/guidelines.xhtml

Figure legends:

Figure 1. Decision tree for health states in the treatment of breast cancer with utility values (median (interquartile range)) for each state

Figure 2 Two way sensitivity analysis according to probability under referral and triple asesssment (four levels) at incremental probability levels under watchful waiting

For peer review only

For peer review only - http://bmjopen.bmj.com/site/about/guidelines.xhtml

References

1. National Cancer Registry: **Cancer in Ireland 1994-2011: Annual report of the National Cancer Registry 2014**. In. Cork: National Cancer Registry; 2014.
2. O'Rourke N: **Review of Referral Patterns and Triage Processes in Symptomatic Breast Units - a Hospital Perspective**. In.: HSE; 2012: 72.
3. National Cancer Control Programme: **Review of activity in the symptomatic breast clinics in the eight designated centres, 2005 and 2006 compared with 2009 year to date.** . In.; 2009.
4. National Cancer Control: **Report on the Symptomatic Breast Disease Key Performance Indicators 2010.** In.; 2011.
5. Keogh C, Wallace E, O'Brien KK, Galvin R, Smith SM, Lewis C, Cummins A, Cousins G, Dimitrov BD, Fahey T: **Developing an international register of clinical prediction rules for use in primary care: a descriptive analysis**. *Annals of family medicine* 2014, **12**(4):359-366.
6. McCowan C, Donnan PT, Dewar J, Thompson A, Fahey T: **Identifying suspected breast cancer: development and validation of a clinical prediction rule**. *The British journal of general practice : the journal of the Royal College of General Practitioners* 2011, **61**(586):e205-214.
7. Galvin R, Joyce D, Downey E, Boland F, Fahey T, Hill AK: **Development and validation of a clinical prediction rule to identify suspected breast cancer: a prospective cohort study**. *BMC cancer* 2014, **14**(1):743.
8. Detsky AS NG, Krahn MD, Naimark D, Redelmeier DA.: **Primer on medical desicion analysis: Part 1 - getting started.** *Medical Decision Making* 1997, **17**(125).
9. Havrilesky LJ BG, Davis DM, Nolte KC, Barnett JC, Myers ER et al. : **Determination of quality of life-related utilities for health states relevant of ovarian cancer diagnosis and treatment.** *Gynecologic Oncology* 2009, **113**:216-220.
10. Protheroe J FT, Montgomery AA, Peters TJ. : **The impact of patients' preferences on the treatment of artial fibrilation: observational study of patient based decision analysis.** *The British Medical Journal* 2000.
11. Allan S, Detsky MD, Naglie G, Krahn MD, Redelmeier DA, Naimark D. **Primer on Medical Decision Analysis: Part 2-Building a Tree**. *Medical Decision Making* 1997. 17(2):126-35.
12. Lee A, Goynt GM, Ho AMH, Keitz S, McGinn T, Wyer P, EBM Teaching Scripts Working Group. **Tips for Teachers of Evidence-based Medicine: Making Sense of Decision Analysis Using a Decision Tree**. *Journal of General Internal Medicine* 2009. 24(5): 642–648
13. von Neumann H, Morgenstern O. **Theory of games and economic behaviour**. 1944. Princeton, NJ: Princeton University Press.
14. Drummond M SM, Torrance GW, O' Brien BJ, Stoddart GL: **Methods for the Economic Evaluation of Health Care Programmes.** Oxford New York: Oxford University Press; 2005.
15. National Cancer Registry: Breast Cancer Incidence, Mortality, Treatment and Survival in Ireland: 1994-2009. National Cancer Registry, Cork, Ireland 2012.
16. Davidson A, Chia S, Olson R, Nichol A, Speers C, Coldman AJ, Bajdik C, Woods R, Tyldesley S: **Stage, treatment and outcomes for patients with breast cancer in British Columbia in 2002: a population-based cohort study**. *CMAJ open* 2013, **1**(4):E134-141.
17. Louwman WJ, Voogd AC, van Dijck JA, Nieuwenhuijzen GA, Ribot J, Pruijt JF, Coebergh JW: **On the rising trends of incidence and prognosis for breast cancer patients diagnosed 1975-2004: a long-term population-based study in southeastern Netherlands**. *Cancer causes & control : CCC* 2008, **19**(1):97-106.

For peer review only - http://bmjopen.bmj.com/site/about/guidelines.xhtml

18. Keegan THM, Press DJ, Tao L, DeRouen MC, Kurian AW, Clarke CA, Gomez SL: **Impact of breast cancer subtypes on3-year survival among adolescent and young adult women**. *Breast Cancer Research* 2013: 15(5):R95.

19. Morris CR, Ramirez CN, Cook SN, Parikh-Patel A, Kizer KW, Bates JH, Snipes KP. **Cancer Stage at Diagnosis**, 2013. Sacramento, CA: California Department of Public Health, California Cancer Registry.

20. Wang Q., Li J, Zheng S, Li J-L, Pang Y, Huang R, Zhang B-N, Zhang B, Yang H-J, Xie X-M, Tang Z-H, Li H, He J-J, Fan J-H, Qiao Y-L. **Breast cancer stage at diagnosis and area-based socioeconomic status: a multicenter 10-year retrospective clinical epidemiological study in China**. *BMC Cancer* 2012. 12:122.

21. Walters S, Maringe C, Butler J, Rachet B, Barrett-Lee P , Bergh J, Boyages J , Christiansen P , Lee M, Wärnberg F, Allemani C , Engholm G , Fornander T, Gjerstorff ML, Johannesen TB, Lawrence G, McGahan CE, Middleton R, Steward J, Tracey E, Turner D, Richards MA, Coleman MP, The ICBP Module 1 Working Group. **Breast cancer survival and stage at diagnosis in Australia, Canada, Denmark, Norway, Sweden and the UK, 2000-2007: a population-based study**. *British Journal of Cancer* 2013. 108, 1195–1208.

22. Campbell C, Durning P, Cheema I, Naisby G. **A simple tool for rapid access to a symptomatic breast clinic**. *European Journal of Surgical Oncology* 2004**.** 30(3):248–251.

23. McCowan C, Donnan PT, Dewar J, Thompson A, Fahey T. **Identifying suspected breast cancer: development and validation of a clinical prediction rule**. *British Journal of General Practice* 2011. 61(585): e205-e214.

24. Cochrane RA, Singhal H, Monypenny IJ, Webster DJ, Lyons K, Mansel RE: **Evaluation of general practitioner referrals to a specialist breast clinic according to the UK national guidelines**. *European journal of surgical oncology : the journal of the European Society of Surgical Oncology and the British Association of Surgical Oncology* 1997, **23**(3):198-201.

25. Nichols S, Waters WE, Wheeler MJ. **Management of female breast disease by Southampton general practioners**. *British Medical Journal* 1980. 281: 1450-1453.

26. Dohrmann PJ, Hugher ESR, McDermott, Price A. **Sympton Duration, tumor staging and survival in patients with carcinoma of the breast**. *Surgery, Gynecology & Obstetrics* 1982. 154: 707-710.

27. Charlson ME. **Delay in the treatment of carcinoma of the breast.** *Surgery, Gynecology & Obstetrics* 1985. 160:393-399.

28. Givio (Interdisciplinary Group for Cancer Care Evaluation) Italy. **Reducing Diagnostic Delay in Breast Cancer: Possible Therapeutic Implications**. *Cancer* 1986. 58:1756-1761

29. Murray DK, Naglie G, Naimark D, Redelmeier DA, Detsky AS. **Primer on Medical Decision Analysis: Part 4-Analyzing the Model and Interpreting the Results**. *Medical Decision Making* 1997. 17(2):142-51.

30. Sendi PP, Clemen RT: **Sensitivity analysis on a chance node with more than two branches**. *Medical decision making : an international journal of the Society for Medical Decision Making* 1999, **19**(4):499-502.

31. StataCorp LP: **STATA User's Guide Release 13**. In. College Station, Texas; 2013.

32. Buyukdamgaci-Alogan G, Elele T, Hayran M, Erman M, Kilickap S: **A decision-analytic model for early stage breast cancer: lumpectomy vs mastectomy**. *Neoplasma* 2008, **55**(3):222-228.

33. Chie WC, Huang CS, Chen JH, Chang KJ: **Utility assessment for different clinical phases of breast cancer in Taiwan**. *Journal of the Formosan Medical Association = Taiwan yi zhi* 2000, **99**(9):677-683.

For peer review only - http://bmjopen.bmj.com/site/about/guidelines.xhtml

34. Sinno H, Izadpanah A, Vorstenbosch J, Dionisopoulos T, Ibrahim AM, Tobias AM, Lee BT, Lin SJ: **Living with a unilateral mastectomy defect: a utility assessment and outcomes study**. *Journal of reconstructive microsurgery* 2014, **30**(5):313-318.
35. McCaffery K, Nickel B, Moynihan R, Hersch J, Teixeira-Pinto A, Irwig L, Barratt A: **How different terminology for ductal carcinoma in situ impacts women's concern and treatment preferences: a randomised comparison within a national community survey**. *BMJ open* 2015, **5**(11):e008094.
36. Mukai TO, Bro F, Olesen F, Vedsted P: **To test or not: a registry-based observational study of an online decision support for prostate-specific antigen tests**. *International journal of medical informatics* 2013, **82**(10):973-979.
37. Esserman L, Shieh Y, Thompson I: **Rethinking screening for breast cancer and prostate cancer**. *Jama* 2009, **302**(15):1685-1692.
38. Hall IJ, Lee Smith J: **Evolution of a CDC Public Health Research Agenda for Low-Risk Prostate Cancer**. *American journal of preventive medicine* 2015, **49**(6s5):S483-s488.
39. Hersch J, Jansen J, Barratt A, Irwig L, Houssami N, Howard K, Dhillon H, McCaffery K: **Women's views on overdiagnosis in breast cancer screening: a qualitative study**. *BMJ (Clinical research ed)* 2013, **346**:f158.

For peer review only

For peer review only - http://bmjopen.bmj.com/site/about/guidelines.xhtml

Table 1 Probability estimates used in the breast cancer decision tree analysis

Treatment path	Outcome	Estimated probability (95% CI)	References
Referral & Triple Assessment (RTA)	Breast cancer diagnosed subsequent to RTA	0.06 (0.05,0.08)	7,22,23,24
	Stage 1 breast cancer among patients diagnosed with breast cancer following RTA	0.34 (0.26,0.42)	15,16,17,18,19,20,21
	Stage 2 breast cancer among patients diagnosed with breast cancer following RTA	0.46 (0.39, 0.52)	15,16,17,18,19,20,21
	Stage 3 breast cancer among patients diagnosed with breast cancer following RTA	0.15 (0.13,0.17)	15,16,17,18,19,20,21
	Stage 4 breast cancer among patients diagnosed with breast cancer following RTA	0.05 (0.04,0.06)	15,16,17,18,19,20,21
Watchful waiting (WW)	Symptoms do not resolve/worsen and patient returns to GP following period of WW	0.34 (0.28,0.41)	25
	Breast cancer diagnosed among patients who return to GP following WW	0.10 (0.05,0.18)	Clinical estimate
	Stage 1 breast cancer among patients diagnosed with breast cancer following WW	0.27 (0.20,0.33)	26,27,28
	Stage 2 breast cancer among patients diagnosed with breast cancer following WW	0.42 (0.36,0.47)	26,27,28
	Stage 3 breast cancer among patients diagnosed with breast cancer following WW	0.20 (0.16,0.22)	26,27,28
	Stage 4 breast cancer among patients diagnosed with breast cancer following WW	0.11 (0.09,0.13)	26,27,28

For peer review only

Table 2. Descriptive statistics for the study sample (n=35 participants)

Variable		Median (Interquartile range)
Age (years)		41 (35 to 47)
Self-rated knowledge of breast cancer (maximum score 10)		5 (4 to 7)
Actual knowledge of breast cancer (maximum score 7)		3 (2 to 4)
		**Number of participants (%)**
Location	Dublin & surrounds	23 (65.7%)
	West of Ireland	10 (28.6%)
	Overseas	2 (5.7%)
Education	Secondary	3 (8.6%)
	Third level	32 (91.4%)
Employed	Yes	32 (91.4%)
	No	3 (8.6%)
Family carer	Yes	18 (51.4%)
	No	17 (49.6%)
Know someone with breast cancer	Yes	30 (85.7%)
	No	5 (14.3%)
Have had a mammography	Yes	15 (42.9%)
	No	20 (57.1%)

For peer review only

Table 3. Sensitivity analysis for breast cancer patient treatment preference study

Health scenario	Values for sensitivity analysis			Number of patients preferring WW to RTA			Swing	$Swing^2$	$\%Swing^2$
	Lower limit for probability	Reference probability	Upper limit for probability	Lower limit for probability	Reference probability	Upper limit for probability			
Diagnosis of breast cancer following RTA	0.05	0.06	0.08	23	25	27	4	16	3.21
Stage 1 breast cancer among patients diagnosed following RTA	0.26	0.34	0.42	26	25	21	-5	25	5.02
Stage 2 breast cancer among patients diagnosed following RTA	0.39	0.46	0.52	26	25	24	-2	4	0.80
Stage 3 breast cancer among patients diagnosed following RTA	0.13	0.15	0.17	25	25	25	0	0	0.00
Stage 4 breast cancer among patients diagnosed following RTA	0.04	0.05	0.06	25	25	25	0	0	0.00
Symptoms do not resolve/worsen and patient returns to GP following period of WW	0.28	0.34	0.41	27	25	24	-3	9	1.81
Diagnosis of breast cancer among patients returning to GP after period of watchful waiting	0.05	0.10	0.18	31	25	10	-21	441	88.55
Stage 1 breast cancer among patients diagnosed following WW	0.20	0.26	0.33	25	25	26	1	1	0.20
Stage 2 breast cancer among patients diagnosed following WW	0.36	0.41	0.47	25	25	26	1	1	0.20
Stage 3 breast cancer among patients diagnosed following WW	0.16	0.19	0.22	25	25	25	0	0	0
Stage 4 breast cancer among patients diagnosed following WW	0.09	0.11	0.13	26	25	25	1	-1	0.20

RTA: Referral and Triple Assessment ; WW: Watchful Waiting

Swing=Number of patients preferring WW to RTA (upper limit for probability)- number of patients preferring WW to RTA (lower limit for probability)

$Swing^2$=Swing*Swing

%Swing= $Swing^2$ x100 /(Total $Swing^2$ (=498))

For peer review only

For peer review only

BMJ Open

Quantifying patient preferences for symptomatic breast clinic referral: a decision analysis study

Journal:	*BMJ Open*
Manuscript ID	bmjopen-2017-017286.R2
Article Type:	Research
Date Submitted by the Author:	02-Feb-2018
Complete List of Authors:	Quinlan, Aisling; Royal College of Surgeons in Ireland, Department of General Practice<br>O'Brien, Kirsty; Royal College of Surgeons in Ireland, Department of General Practice<br>Galvin, Rose; University of Limerick, 3Department of Clinical Therapies<br>Hardy, Colin; Royal College of Surgeons in Ireland, Department of General Practice<br>McDonnell, Ronan; Royal College of Surgeons in Ireland, Department of General Practice<br>Joyce, Doireann; Royal College of Surgeons in Ireland, Department of General Practice<br>McDowell, Ronald; Royal College of Surgeons in Ireland, HRB Centre for Primary Care Research<br>Aherne, Emma; Royal College of Surgeons in Ireland, Department of General Practice<br>Keogh, Claire; RCSI, HRB Centre for Primary Care Research<br>O'Sullivan, Katriona; University of Dublin Trinity College<br>Fahey, Tom; Royal College of Surgeons in Ireland, Department of General Practice
**Primary Subject Heading**:	Evidence based practice
Secondary Subject Heading:	Diagnostics, Evidence based practice, Oncology
Keywords:	EPIDEMIOLOGY, ONCOLOGY, Quality in health care < HEALTH SERVICES ADMINISTRATION & MANAGEMENT

SCHOLARONE™ Manuscripts

Quantifying patient preferences for symptomatic breast clinic referral: a decision analysis study

Aisling Quinlan[1], BSc, Kirsty K O'Brien[1], PhD, Rose Galvin[1,3], PhD, Colin Hardy[1], MSc, Ronan McDonnell[1], PhD, Doireann Joyce[2], MB Ronald D.McDowell[1], PhD, Emma Aherne[1],MB, Claire Keogh[1], PhD, Katriona O'Sullivan[1,4], PhD, Tom Fahey[1], MD

[1]HRB Centre for Primary Care Research, Department of General Practice, Royal College of Surgeons in Ireland (RCSI), 123 St.Stephens Green, Dublin 2, Republic of Ireland.

[2]National University of Ireland Galway, University Road, Galway. Republic of Ireland.

[3]Department of Clinical Therapies, University of Limerick, Limerick.

[4]School of Computer Science and Statistics, Trinity College Dublin.

*Corresponding Author

Tom Fahey, HRB Centre for Primary Care Research, Department of General Practice, Royal College of Surgeons in Ireland, 123 St. Stephen's Green, Dublin 2, Republic of Ireland.

Email: tomfahey@rcsi.ie

Word count:

For peer review only

Abstract

Objectives: Decision analysis study that incorporates patient preferences and probability estimates to investigate the impact of women's preferences for referral or an alternative strategy of watchful waiting if faced with symptoms that could be due to breast cancer.

Setting: Community-based study.

Participants: Asymptomatic women aged 30-60 years.

Interventions: Participants were presented with 11 health scenarios that represent the possible consequences of symptomatic breast problems. Participants were asked the risk of death that they were willing to take in order to avoid the health scenario using the standard gamble (SG) utility method. This process was repeated for all 11 health scenarios. Formal decision analysis for the preferred individual decision was then estimated for each participant.

Primary outcome measure: The preferred diagnostic strategy, either watchful waiting or referral to a breast clinic. Sensitivity analysis was used to examine how each varied according to changes in the probabilities of the health scenarios.

Results: A total of 35 participants completed the interviews, with median age 41 years (Interquartile range 35 to 47 years). The majority of the study sample were employed (n=32, 91.4%), with a third-level (university) education (n=32, 91.4%) and with knowledge of someone with breast cancer (n=30, 85.7%). When individual preferences were accounted for, 25 (71.4%) patients preferred watchful waiting to referral for triple assessment as their preferred initial diagnostic strategy. Sensitivity analysis shows that referral for triple assessment becomes the dominant strategy at the upper probability estimate (18%) of breast cancer in the community.

Conclusions: Watchful waiting is an acceptable strategy for most women who present to their GP with breast symptoms. These findings suggest that current referral guidelines should take more explicit account of women's preferences in relation in terms of the initial diagnostic strategy for symptomatic breast problems.

For peer review only - http://bmjopen.bmj.com/site/about/guidelines.xhtml

Strengths and limitations of this study

Strength of this study is that it accounts for women's preferences in relation to a watchful waiting or immediate referral strategy when faced with symptoms that could be due to breast cancer.

Weakness is that preferences were elicited in asymptomatic women. Women with symptoms may express different preferences.

The impact of eliciting preferences should be done in the context of a randomized trial of a decision aid that uses decision analysis as a form of shared decision making for women with symptoms that could be due to breast cancer.

Taking account of women's preferences might mean that significantly fewer women would be referred to symptomatic breast clinics with a consequent saving in investigations and costs, and reducing the burden of "medicalization" in women with benign breast disease.

For peer review only

For peer review only - http://bmjopen.bmj.com/site/about/guidelines.xhtml

Introduction

Between 2009-2011, it was reported that 31% of cancers in Irish women were due to breast cancer, making it the most prevalent cancer in Irish women [1]. In response, the National Cancer Control Programme (NCCP) clinical guidelines were introduced to streamline the referral process to symptomatic breast units. At present, GPs act as gatekeepers for referral of women with breast symptoms. Referral to symptomatic breast clinics results in triple assessment: a three step process comprising of clinical, radiological and histological examination after fine needle aspiration [2]. Over the past ten years, the number of women referred to symptomatic breast clinics in Ireland has significantly increased, despite the fact there has been no rise in breast cancer incidence [2]. In 2006, there were 23,575 new referrals with 2137 breast cancers cases diagnosed; in 2009 there were32,249 referrals and 1879 breast cancer cases diagnosed; and in 2010, 37,631 new referrals with 2012 new breast cases cancers diagnosed [3][4]. Consequently the malignant:benign ratio has altered from 1:10 in 2006, to 1:16 in 2009 to 1:18 in 2010 [4]. The rise in these referrals for triple assessment without an increase in breast cancer diagnosis means that more women are being exposed to invasive diagnostic investigations.

Clinical prediction rules (CPRs) are clinical tools that quantify the independent impact of factors from a patients history, physical examination and diagnostic tests and stratify patients according to the probability of having a target disorder [5]. Recently, CPRs have been derived and validated to calculate a patient's risk of breast cancer [6, 7], and have been proposed as methods to discriminate between patients at high risk of breast cancer from low risk patients. Such an approach may serve to decrease the number of unnecessary referrals to symptomatic breast clinics in women with a low probability of breast cancer. Patients who are considered low risk could, as an alternative, undergo a watchful waiting strategy (up to 6-8 weeks) with their GP. However, it is unclear from the current literature if patients who are considered low risk would find this acceptable.

Decision analysis is a technique to aid decision making when uncertainty exists over the balance between benefits and risks of treatment [8]. Decision trees have been utilised to assess how patient's preferences impact on diagnosis and treatment for conditions such as ovarian cancer

For peer review only - http://bmjopen.bmj.com/site/about/guidelines.xhtml

[9] and atrial fibrillation [10]. To date, little is known about how women who have symptomatic breast problems feel about the options of immediate referral or watching waiting in terms of referral to a symptomatic breast clinic. The aim of this study is assess the impact of patient preferences for immediate referral to a specialist breast clinic or watchful waiting in primary care. We developed a decision tree and elicited patient's preferences for these alternative diagnostic strategies by combining individual preferences with probability estimates of breast cancer using decision analysis.

Methodology

Study design

This study utilized decision analysis in the form of utility assessment and folding back a decision tree. The STROBE standardized reporting guidelines were followed to ensure the standardized conduct and reporting of the research. Ethical approval was received from the Research Ethics Committee at the Royal College of Surgeons in Ireland.

Developing the decision tree and health scenarios

Literature searches were conducted in medical databases such as PubMed, the Cochrane library and Web of Science to assist with the development of the decision tree and health scenarios. A specialist registrar with clinical experience in a symptomatic breast clinic was also consulted during this development phase. Following these consultations, the decision tree was constructed to portray the temporal course of the two diagnostic options (Figure 1). The decision tree had one decision node with two diagnostic branches (a choice between watchful waiting or immediate referral), nine probability or chance nodes and eleven health outcome nodes. In accordance with the literature the tree was designed such that only two branches emanated from each chance node [11]. The number of outcome nodes differed between the two branches of the tree in order to accommodate the additional step (an observation period before breast cancer assessment among patients who return to their GP) in the watchful waiting branch of the tree. It is not always possible or appropriate for each branch of a decision

For peer review only - http://bmjopen.bmj.com/site/about/guidelines.xhtml

tree to have the same number of outcome nodes as this will be dependent on the nature and temporal order of events in that branch [12]. The decision tree is “balanced” as each branch has advantages and disadvantages and hence it is not possible to readily identify a treatment path that is ideal in all respects [11].

A total of eleven health scenarios were constructed; one for each outcome node. For this study the standard gamble (SG) method of utility assessment was used [13] [14]. Briefly, participants were presented with a description of each health state, and its physical consequences and emotional consequences, and were given the opportunity to remain in the health state or to gamble it for perfect health, but with the risk of death if they took the gamble. Participants were presented with differing probabilities showing how the chance of perfect health and death varied with each other for each health state (the “ping-pong method”). The utility value was one minus the risk of death they were willing to take in order to avoid the health state described. The 11 health states were presented in a random order. Details of the health states and the standard gamble scenarios seen by the participants are provided in the supplementary materials section.

Probability estimates for each chance node were obtained from the medical literature [7,15-28]. Where possible, relevant probability estimates for each chance node were combined using random effects meta-analysis (Table 1). The same probabilities were used for each participant. It was not possible to estimate from the literature the probability of a diagnosis of breast cancer among patients who returned to their doctor following a period of watchful waiting. Clinical experience suggested this would be several percentage points higher in absolute terms than the probability of a diagnosis of breast cancer following RTA and an estimate of 10% was used.

For peer review only - http://bmjopen.bmj.com/site/about/guidelines.xhtml

Participants and setting

Interviews were conducted over a six month period in Dublin from October 2014 to April 2015. Asymptomatic women aged 30-60 years were invited to participate in the study. Participants were recruited using university staff notice boards, social media and snowball sampling. The majority of interviews were carried out in a meeting room in the research building. Some interviews were conducted in peoples' place of work as this was more convenient for them. Written informed consent was obtained from all participants prior to participation. The interviews were conducted with a researcher present, who was available to answer any questions and to obtain feedback about the interview. Prior to the interview, participants were given a baseline demographics questionnaire and a multiple-choice questionnaire to assess their knowledge of breast cancer. As this is a feasibility study for a proposed randomized controlled trial, no formal sample size was calculated. We excluded women with a current or past history of breast cancer.

Utility assessment

A computer-based assessment programme which presented and recorded participant responses was designed. This programme incorporated an introduction, an explanation of the interview process, and presentation of each clinical scenario in random order using the standard gamble method of utility assessment. An example of the standard gamble was included for orientation and definitions were provided for any medical terminology used. The purpose of using a computer programme was to allow the interviewer to limit their interaction with each participant, standardize utility assessment and minimize interviewer bias. A pilot study was carried out and modifications made to the computer programme and utility assessment process. See supplementary table of health scenarios.

Ethical approval and data Protection

The data from the computer programme was stored with a participant ID only and saved to an encrypted password protected desktop computer. To protect patient identity, a MS excel

For peer review only - http://bmjopen.bmj.com/site/about/guidelines.xhtml

spreadsheet was created; containing just patient names and identification numbers, and this was stored in a secured, password protected folder on the server and kept separate to the data from the interview.

Decision analysis

The utility for each outcome, “a number assigned to the quality of life a patient would attach to a particular outcome on a defined scale “ [12], was calculated as 1-the risk of death (expressed as a fraction) a participant was willing to take in order to avoid the health scenario as described. For each participant, an expected utility score for each diagnostic option was obtained by multiplying and summing the relevant probabilities and utilities. The branch which had the highest expected utility score was determined to be the preferred diagnostic strategy- watchful waiting or referral for triple assessment.

Sensitivity analysis was used to determine the robustness of the conclusion drawn from the decision tree [29]. For our study each of the probability estimates derived from the literature was allowed to vary between the lower and upper limits of the 95% confidence interval for that estimate, in order to assess the extent to which the preferred diagnostic strategy changed. The difference between the number of patients preferring watchful waiting using the upper limit of the 95% confidence interval and the number of patients preferring watchful waiting using the lower limit of the 95% confidence interval for each probability estimate (the swing) was squared and totalled; the impact of varying each of the probability estimates was measured as a percentage of the total squared-swing. The sensitivity analyses accounted for the fact that some probability estimates were used more than once by incorporating the conditioning event into the definition of the variables under consideration [30]. An additional two-way sensitivity analysis explored how women’s preferred diagnostic option changed as the probability of diagnosis following watchful waiting varied relative to the probability of diagnosis following referral and triple assessment.

For peer review only - http://bmjopen.bmj.com/site/about/guidelines.xhtml

Standard descriptive statistics were used to summarise the sample. Results were held to be significant if they refer to statistical significance on a two-sided test evaluated at the 5% level. All analyses were performed using STATA 13 [31].

For peer review only

For peer review only - http://bmjopen.bmj.com/site/about/guidelines.xhtml

Results

A total of 36 women participated, with 35 interviews completed in full. The median age of participants was 41 years (interquartile range (IQR) 35 to 47 years). The demographic details of participants are described in Table 2. The majority of participants were employed (32, 91.4%), had a third-level (university) education (32, 91.4%) and knew someone with breast cancer (30, 85.7%). Less than half the study sample (15, 42.9%) reported having a prior mammography. There was borderline evidence of a negative correlation between participants' self-reported knowledge of breast cancer and their actual knowledge based on responses to a multiple-choice questionnaire (p=0.06).

When patient preferences were taken into account, 25 patients (71.4%, 95%CI 19%, 30%) preferred watchful waiting to referral as the initial diagnostic strategy. Sensitivity analysis showed that the overall preferred diagnostic strategy changed to referral when the probability of breast cancer following a period of watchful waiting increased; only ten women favoured watchful waiting when the probability of breast cancer following a period of watchful waiting had increased to 18% (Table 3). Swings in the group preference according to changes in the probabilities associated with each of the stage of breast cancer were small in comparison (Table 3).

In terms of two-way sensitivity analysis more than half the participating women still preferred watchful waiting when the probability of a diagnosis of breast cancer following a period of watchful waiting was 2.25 times as high as the probability of diagnosis following referral and triple assessment. (Figure 2) This pattern remained consistent irrespective of the probability of breast cancer following referral and triple assessment, estimated at levels of 4%, 6%, 8% and 10%.

For peer review only - http://bmjopen.bmj.com/site/about/guidelines.xhtml

Discussion

Summary of main findings

This study shows that a strategy of watching waiting in women with breast symptoms in primary care could be considered as an acceptable alternative to immediate referral to a symptomatic breast clinic, but this strategy of shared decision making using decision analysis should be tested in a randomized controlled trial. Our observational findings suggest that women's own preferences should form an important element of the decision to refer to a symptomatic breast clinic and that these preferences should be accounted as part of a shared decision making process. Sensitivity analysis shows that as the probability of breast cancer increases, the diagnostic strategy of referral for triple assessment dominates. Two way sensitivity analysis shows that, across the likely probability range of breast cancer under a watchful waiting strategy, referral for triple assessment becomes the dominant strategy once the probability of breast cancer is two and a half times more likely than under a watchful waiting strategy.

Results in the context of the current literature

Although utility assessment has been used to evaluate the acceptability of various clinical states and treatment regimes in the breast cancer literature [32-34] it has not been used to examine the acceptability of watchful waiting in low risk patients presenting with signs and symptoms of possible breast cancer. A previous study that assessed the effects of alternative terms for ductal carcinoma in-situ (DCIS). found that watchful waiting is acceptable to patients provided that research shows that watchful waiting is a safe and effective option [35]. The preliminary findings from our study suggest that women may be prepared to accept a conservative, non-invasive strategy of watchful waiting when the probability of breast cancer is low.

Watchful waiting has also been described as a key technique for more effective management of other cancers such as prostate cancer [36]. In prostate cancer the use of PSA testing has led to an increase in the incidence of prostate cancer but not a decrease in the mortality rate, suggesting that PSA testing often identifies low risk cancers [37]. Given the serious and

For peer review only - http://bmjopen.bmj.com/site/about/guidelines.xhtml

common side effects associated with treatment in prostate cancer some studies have shown that some men prefer watchful waiting to treatment options [38]. In our study the side effects of further diagnostic testing (triple assessment) are less onerous than treatment of localized prostate cancer, but many women would experience high levels of anxiety and stress upon referral to a symptomatic breast clinic. This study suggests that many women would find it more acceptable to undergo watchful waiting for a period rather than participate in diagnostic testing when their risk of breast cancer is low.

A qualitative study examining women's views on over-diagnosis in breast cancer screening showed that 50% of the study population felt they needed to make more careful personal decisions about screening and would consider watchful waiting as an alternative route if they were found to have a low risk cancer [39]. Like our study watchful waiting was found to be an acceptable strategy when the risk involved is low and the health outcomes are positive.

Strengths and limitations

This study has several strengths. We used an evidence-based approach, quantifying probabilities from the literature, and developing health states that incorporate the common features of breast cancer. We tested and modified the wording of the health states and the computer programme in a pilot group of participants and refined our protocol based on feedback from these participants. We tried to limit any bias caused by the interviewer by using a computer programme and providing the participant with written definitions and information. Examples of the standard gamble were given to the participants prior to the interview, so participants had the opportunity to familiarize themselves with the method. Although the sample was relatively small, it included participants with a range of ages and knowledge of breast cancer. We acknowledge that the sample of participants is not representative of the population at large; that women were asymptomatic and that preferences may change or be different when faced with possible symptoms of breast cancer- a breast lump or nipple change. However, our results suggest that when preferences about a watchful waiting strategy is elicited, many women are happy to adopt this approach. Based on these provisional results, we

For peer review only - http://bmjopen.bmj.com/site/about/guidelines.xhtml

For peer review only

hope to develop a decision aid for women with symptomatic breast problems and test its value in a randomized controlled trial.

Clinical and policy Implications

The current referral process leads to many patients who are at low risk of breast cancer being referred to busy symptomatic breast clinics, with a risk of iatrogenic harm, increased patient anxiety and over-medicalizing of a self-limiting condition. With more emphasis on the development of clinical prediction rules, it will be possible to identify and quantify each individual women's risk of breast cancer. A recent study derived a clinical prediction rule (CPR) for predicting the risk of breast cancer for women presenting to their GP with breast symptoms.[7] A total of 6,590 patients were included in the derivation study and 4.9% were diagnosed with breast cancer. Independent clinical predictors for breast cancer were: increasing age by year (adjusted OR 1.08, 95%CI 1.07-1.09); presence of a lump (5.63, 95%CI 4.2-7.56); nipple change (2.77, 95%CI 1.68-4.58) and nipple discharge (2.09, 95% CI 1.1-3.97). Validation of the rule (n=911) also shows that the probability of breast cancer is higher with an increasing number of these independent clinical variables. By incorporating patient preferences with an individual's probability of breast cancer from a CPR, a strategy of watchful waiting is acceptable, cost effective and likely to avoid considerable iatrogenic harm. Clinical practice guidelines should incorporate probability estimates of breast cancer based on CPRs and urge elicitation of patient preference for alternative diagnostic options- watchful waiting or referral. More formally, decision aids that could be developed in conjunction with the symptomatic breast CPRs to enable the alternative options of watchful waiting or referral based on individual preferences.

Conclusion

This study suggests that watchful waiting may be an acceptable diagnostic strategy for women who present to their general practitioner with breast symptoms. These findings should be

For peer review only - http://bmjopen.bmj.com/site/about/guidelines.xhtml

validated in a larger and more comprehensive study of patient preferences in women with breast symptoms in a randomized controlled trial. Clinical practice guidelines should reflect the importance of eliciting women's preferences for watchful waiting as an alternative to referral to a symptomatic breast clinic, particularly in women with low risk clinical features.

Competing interests

The authors declare that they have no competing interests.

Authors' contributions

All authors AQ, KOB, RG, CH, RMcD, DJ, RDMcD, EA, CK, KO'S, TF were involved in the study conception and design. AQ, RG and KOB acquired data for analysis. RMcD designed the computer-based utility analysis programme. CH and RDMcD performed statistical analysis. AQ interpreted the data and drafted the paper. KOB, RG, RDMcD and TF critically revised the draft manuscript. All authors read and approved the final manuscript.

Funding statement

Health Research Board of Ireland grant number HRC-2007-1.

Data sharing statement

Extra data can be accessed via the Dryad data repository at DOI: doi:10.5061/dryad.k15f3t5

Figure legends:

Figure 1. Decision tree for health states in the treatment of breast cancer with utility values (median (interquartile range)) for each state

For peer review only - http://bmjopen.bmj.com/site/about/guidelines.xhtml

Figure 2 Two way sensitivity analysis according to probability under referral and triple asesssment (four levels) at incremental probability levels under watchful waiting

For peer review only

For peer review only - http://bmjopen.bmj.com/site/about/guidelines.xhtml

References

1. National Cancer Registry: **Cancer in Ireland 1994-2011: Annual report of the National Cancer Registry 2014**. In. Cork: National Cancer Registry; 2014.
2. O'Rourke N: **Review of Referral Patterns and Triage Processes in Symptomatic Breast Units - a Hospital Perspective**. In.: HSE; 2012: 72.
3. National Cancer Control Programme: **Review of activity in the symptomatic breast clinics in the eight designated centres, 2005 and 2006 compared with 2009 year to date.** . In.; 2009.
4. National Cancer Control: **Report on the Symptomatic Breast Disease Key Performance Indicators 2010.** In.; 2011.
5. Keogh C, Wallace E, O'Brien KK, Galvin R, Smith SM, Lewis C, Cummins A, Cousins G, Dimitrov BD, Fahey T: **Developing an international register of clinical prediction rules for use in primary care: a descriptive analysis**. *Annals of family medicine* 2014, **12**(4):359-366.
6. McCowan C, Donnan PT, Dewar J, Thompson A, Fahey T: **Identifying suspected breast cancer: development and validation of a clinical prediction rule**. *The British journal of general practice : the journal of the Royal College of General Practitioners* 2011, **61**(586):e205-214.
7. Galvin R, Joyce D, Downey E, Boland F, Fahey T, Hill AK: **Development and validation of a clinical prediction rule to identify suspected breast cancer: a prospective cohort study**. *BMC cancer* 2014, **14**(1):743.
8. Detsky AS NG, Krahn MD, Naimark D, Redelmeier DA.: **Primer on medical desicion analysis: Part 1 - getting started.** *Medical Decision Making* 1997, **17**(125).
9. Havrilesky LJ BG, Davis DM, Nolte KC, Barnett JC, Myers ER et al. : **Determination of quality of life-related utilities for health states relevant of ovarian cancer diagnosis and treatment.** *Gynecologic Oncology* 2009, **113**:216-220.
10. Protheroe J FT, Montgomery AA, Peters TJ. : **The impact of patients' preferences on the treatment of artial fibrilation: observational study of patient based decision analysis.** *The British Medical Journal* 2000.
11. Allan S, Detsky MD, Naglie G, Krahn MD, Redelmeier DA, Naimark D. **Primer on Medical Decision Analysis: Part 2-Building a Tree**. *Medical Decision Making* 1997. 17(2):126-35.
12. Lee A, Goynt GM, Ho AMH, Keitz S, McGinn T, Wyer P, EBM Teaching Scripts Working Group. **Tips for Teachers of Evidence-based Medicine: Making Sense of Decision Analysis Using a Decision Tree**. *Journal of General Internal Medicine* 2009. 24(5): 642–648
13. von Neumann H, Morgenstern O. **Theory of games and economic behaviour**. 1944. Princeton, NJ: Princeton University Press.
14. Drummond M SM, Torrance GW, O' Brien BJ, Stoddart GL: **Methods for the Economic Evaluation of Health Care Programmes.** Oxford New York: Oxford University Press; 2005.
15. National Cancer Registry: Breast Cancer Incidence, Mortality, Treatment and Survival in Ireland: 1994-2009. National Cancer Registry, Cork, Ireland 2012.
16. Davidson A, Chia S, Olson R, Nichol A, Speers C, Coldman AJ, Bajdik C, Woods R, Tyldesley S: **Stage, treatment and outcomes for patients with breast cancer in British Columbia in 2002: a population-based cohort study**. *CMAJ open* 2013, **1**(4):E134-141.
17. Louwman WJ, Voogd AC, van Dijck JA, Nieuwenhuijzen GA, Ribot J, Pruijt JF, Coebergh JW: **On the rising trends of incidence and prognosis for breast cancer patients diagnosed 1975-2004: a long-term population-based study in southeastern Netherlands**. *Cancer causes & control : CCC* 2008, **19**(1):97-106.

For peer review only - http://bmjopen.bmj.com/site/about/guidelines.xhtml

18. Keegan THM, Press DJ, Tao L, DeRouen MC, Kurian AW, Clarke CA, Gomez SL: **Impact of breast cancer subtypes on3-year survival among adolescent and young adult women**. *Breast Cancer Research* 2013: 15(5):R95.
19. Morris CR, Ramirez CN, Cook SN, Parikh-Patel A, Kizer KW, Bates JH, Snipes KP. **Cancer Stage at Diagnosis**, 2013. Sacramento, CA: California Department of Public Health, California Cancer Registry.
20. Wang Q., Li J, Zheng S, Li J-L, Pang Y, Huang R, Zhang B-N, Zhang B, Yang H-J, Xie X-M, Tang Z-H, Li H, He J-J, Fan J-H, Qiao Y-L. **Breast cancer stage at diagnosis and area-based socioeconomic status: a multicenter 10-year retrospective clinical epidemiological study in China**. *BMC Cancer* 2012. 12:122.
21. Walters S, Maringe C, Butler J, Rachet B, Barrett-Lee P , Bergh J, Boyages J , Christiansen P , Lee M, Wärnberg F, Allemani C , Engholm G , Fornander T, Gjerstorff ML, Johannesen TB, Lawrence G, McGahan CE, Middleton R, Steward J, Tracey E, Turner D, Richards MA, Coleman MP, The ICBP Module 1 Working Group. **Breast cancer survival and stage at diagnosis in Australia, Canada, Denmark, Norway, Sweden and the UK, 2000-2007: a population-based study**. *British Journal of Cancer* 2013. 108, 1195–1208.
22. Campbell C, Durning P, Cheema I, Naisby G. **A simple tool for rapid access to a symptomatic breast clinic**. *European Journal of Surgical Oncology* 2004**.** 30(3):248–251.
23. McCowan C, Donnan PT, Dewar J, Thompson A, Fahey T. **Identifying suspected breast cancer: development and validation of a clinical prediction rule**. *British Journal of General Practice* 2011. 61(585): e205-e214.
24. Cochrane RA, Singhal H, Monypenny IJ, Webster DJ, Lyons K, Mansel RE: **Evaluation of general practitioner referrals to a specialist breast clinic according to the UK national guidelines**. *European journal of surgical oncology : the journal of the European Society of Surgical Oncology and the British Association of Surgical Oncology* 1997, **23**(3):198-201.
25. Nichols S, Waters WE, Wheeler MJ. **Management of female breast disease by Southampton general practioners**. *British Medical Journal* 1980. 281: 1450-1453.
26. Dohrmann PJ, Hugher ESR, McDermott, Price A. **Sympton Duration, tumor staging and survival in patients with carcinoma of the breast**. *Surgery, Gynecology & Obstetrics* 1982. 154: 707-710.
27. Charlson ME. **Delay in the treatment of carcinoma of the breast.** *Surgery, Gynecology & Obstetrics* 1985. 160:393-399.
28. Givio (Interdisciplinary Group for Cancer Care Evaluation) Italy. **Reducing Diagnostic Delay in Breast Cancer: Possible Therapeutic Implications**. *Cancer* 1986. 58:1756-1761
29. Murray DK, Naglie G, Naimark D, Redelmeier DA, Detsky AS. **Primer on Medical Decision Analysis: Part 4-Analyzing the Model and Interpreting the Results**. *Medical Decision Making* 1997. 17(2):142-51.
30. Sendi PP, Clemen RT: **Sensitivity analysis on a chance node with more than two branches**. *Medical decision making : an international journal of the Society for Medical Decision Making* 1999, **19**(4):499-502.
31. StataCorp LP: **STATA User's Guide Release 13**. In. College Station, Texas; 2013.
32. Buyukdamgaci-Alogan G, Elele T, Hayran M, Erman M, Kilickap S: **A decision-analytic model for early stage breast cancer: lumpectomy vs mastectomy**. *Neoplasma* 2008, **55**(3):222-228.
33. Chie WC, Huang CS, Chen JH, Chang KJ: **Utility assessment for different clinical phases of breast cancer in Taiwan**. *Journal of the Formosan Medical Association = Taiwan yi zhi* 2000, **99**(9):677-683.

For peer review only - http://bmjopen.bmj.com/site/about/guidelines.xhtml

34. Sinno H, Izadpanah A, Vorstenbosch J, Dionisopoulos T, Ibrahim AM, Tobias AM, Lee BT, Lin SJ: **Living with a unilateral mastectomy defect: a utility assessment and outcomes study**. *Journal of reconstructive microsurgery* 2014, **30**(5):313-318.
35. McCaffery K, Nickel B, Moynihan R, Hersch J, Teixeira-Pinto A, Irwig L, Barratt A: **How different terminology for ductal carcinoma in situ impacts women's concern and treatment preferences: a randomised comparison within a national community survey**. *BMJ open* 2015, **5**(11):e008094.
36. Mukai TO, Bro F, Olesen F, Vedsted P: **To test or not: a registry-based observational study of an online decision support for prostate-specific antigen tests**. *International journal of medical informatics* 2013, **82**(10):973-979.
37. Esserman L, Shieh Y, Thompson I: **Rethinking screening for breast cancer and prostate cancer**. *Jama* 2009, **302**(15):1685-1692.
38. Hall IJ, Lee Smith J: **Evolution of a CDC Public Health Research Agenda for Low-Risk Prostate Cancer**. *American journal of preventive medicine* 2015, **49**(6s5):S483-s488.
39. Hersch J, Jansen J, Barratt A, Irwig L, Houssami N, Howard K, Dhillon H, McCaffery K: **Women's views on overdiagnosis in breast cancer screening: a qualitative study**. *BMJ (Clinical research ed)* 2013, **346**:f158.

For peer review only

For peer review only - http://bmjopen.bmj.com/site/about/guidelines.xhtml

Table 1 Probability estimates used in the breast cancer decision tree analysis

Treatment path	Outcome	Estimated probability (95% CI)	References
Referral & Triple Assessment (RTA)	Breast cancer diagnosed subsequent to RTA	0.06 (0.05,0.08)	7,22,23,24
	Stage 1 breast cancer among patients diagnosed with breast cancer following RTA	0.34 (0.26,0.42)	15,16,17,18,19,20,21
	Stage 2 breast cancer among patients diagnosed with breast cancer following RTA	0.46 (0.39, 0.52)	15,16,17,18,19,20,21
	Stage 3 breast cancer among patients diagnosed with breast cancer following RTA	0.15 (0.13,0.17)	15,16,17,18,19,20,21
	Stage 4 breast cancer among patients diagnosed with breast cancer following RTA	0.05 (0.04,0.06)	15,16,17,18,19,20,21
Watchful waiting (WW)	Symptoms do not resolve/worsen and patient returns to GP following period of WW	0.34 (0.28,0.41)	25
	Breast cancer diagnosed among patients who return to GP following WW	0.10 (0.05,0.18)	Clinical estimate
	Stage 1 breast cancer among patients diagnosed with breast cancer following WW	0.27 (0.20,0.33)	26,27,28
	Stage 2 breast cancer among patients diagnosed with breast cancer following WW	0.42 (0.36,0.47)	26,27,28
	Stage 3 breast cancer among patients diagnosed with breast cancer following WW	0.20 (0.16,0.22)	26,27,28
	Stage 4 breast cancer among patients diagnosed with breast cancer following WW	0.11 (0.09,0.13)	26,27,28

For peer review only

Table 2. Descriptive statistics for the study sample (n=35 participants)

**Variable**		**Median (Interquartile range)**
Age (years)		41 (35 to 47)
Self-rated knowledge of breast cancer (maximum score 10)		5 (4 to 7)
Actual knowledge of breast cancer (maximum score 7)		3 (2 to 4)
		**Number of participants (%)**
Location	Dublin & surrounds	23 (65.7%)
	West of Ireland	10 (28.6%)
	Overseas	2 (5.7%)
Education	Secondary school	3 (8.6%)
	Third level (university)	32 (91.4%)
Employed	Yes	32 (91.4%)
	No	3 (8.6%)
Family carer	Yes	18 (51.4%)
	No	17 (49.6%)
Know someone with breast cancer	Yes	30 (85.7%)
	No	5 (14.3%)
Have had a mammography	Yes	15 (42.9%)
	No	20 (57.1%)

For peer review only

For peer review only - http://bmjopen.bmj.com/site/about/guidelines.xhtml

Table 3. Sensitivity analysis for breast cancer patient treatment preference study

Health scenario	Values for sensitivity analysis			Number of patients preferring WW to RTA			Swing	$Swing^2$	$\%Swing^2$
	Lower limit for probability	Reference probability	Upper limit for probability	Lower limit for probability	Reference probability	Upper limit for probability			
Diagnosis of breast cancer following RTA	0.05	0.06	0.08	23	25	27	4	16	3.21
Stage 1 breast cancer among patients diagnosed following RTA	0.26	0.34	0.42	26	25	21	-5	25	5.02
Stage 2 breast cancer among patients diagnosed following RTA	0.39	0.46	0.52	26	25	24	-2	4	0.80
Stage 3 breast cancer among patients diagnosed following RTA	0.13	0.15	0.17	25	25	25	0	0	0.00
Stage 4 breast cancer among patients diagnosed following RTA	0.04	0.05	0.06	25	25	25	0	0	0.00
Symptoms do not resolve/worsen and patient returns to GP following period of WW	0.28	0.34	0.41	27	25	24	-3	9	1.81
Diagnosis of breast cancer among patients returning to GP after period of watchful waiting	0.05	0.10	0.18	31	25	10	-21	441	88.55
Stage 1 breast cancer among patients diagnosed following WW	0.20	0.26	0.33	25	25	26	1	1	0.20
Stage 2 breast cancer among patients diagnosed following WW	0.36	0.41	0.47	25	25	26	1	1	0.20
Stage 3 breast cancer among patients diagnosed following WW	0.16	0.19	0.22	25	25	25	0	0	0
Stage 4 breast cancer among patients diagnosed following WW	0.09	0.11	0.13	26	25	25	1	-1	0.20

RTA: Referral and Triple Assessment ; WW: Watchful Waiting

Swing=Number of patients preferring WW to RTA (upper limit for probability)- number of patients preferring WW to RTA (lower limit for probability)

$Swing^2$=Swing*Swing

%Swing= $Swing^2$ x100 /(Total $Swing^2$ (=498))

For peer review only

For peer review only

For peer review only - http://bmjopen.bmj.com/site/about/guidelines.xhtml

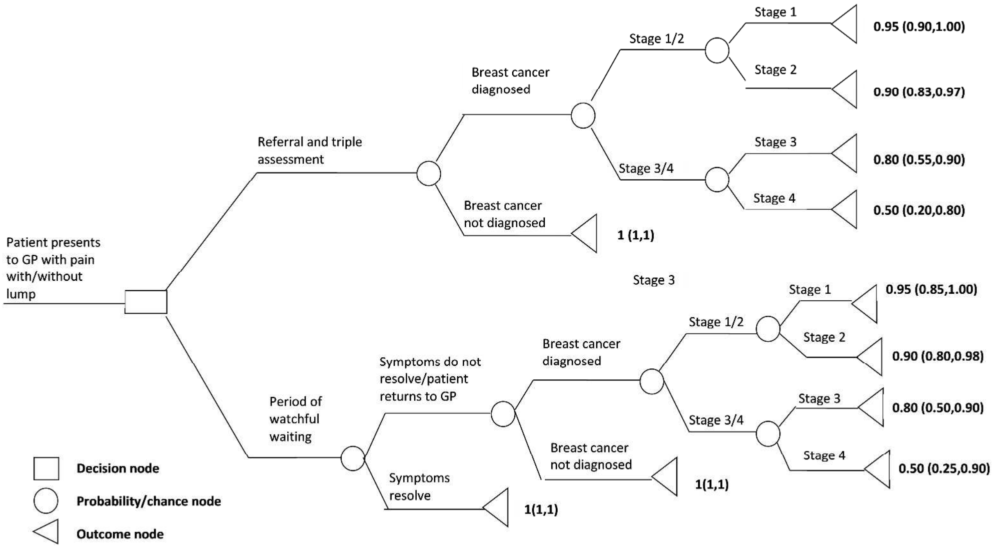

549x300mm (300 x 300 DPI)

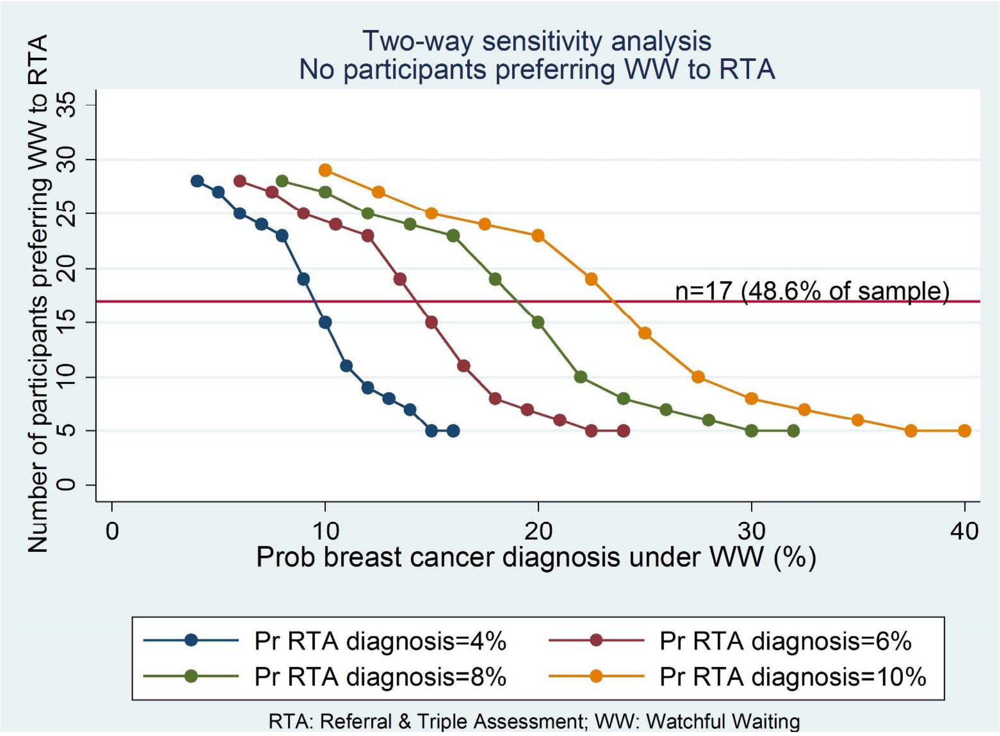

276x201mm (300 x 300 DPI)

5/5/2015

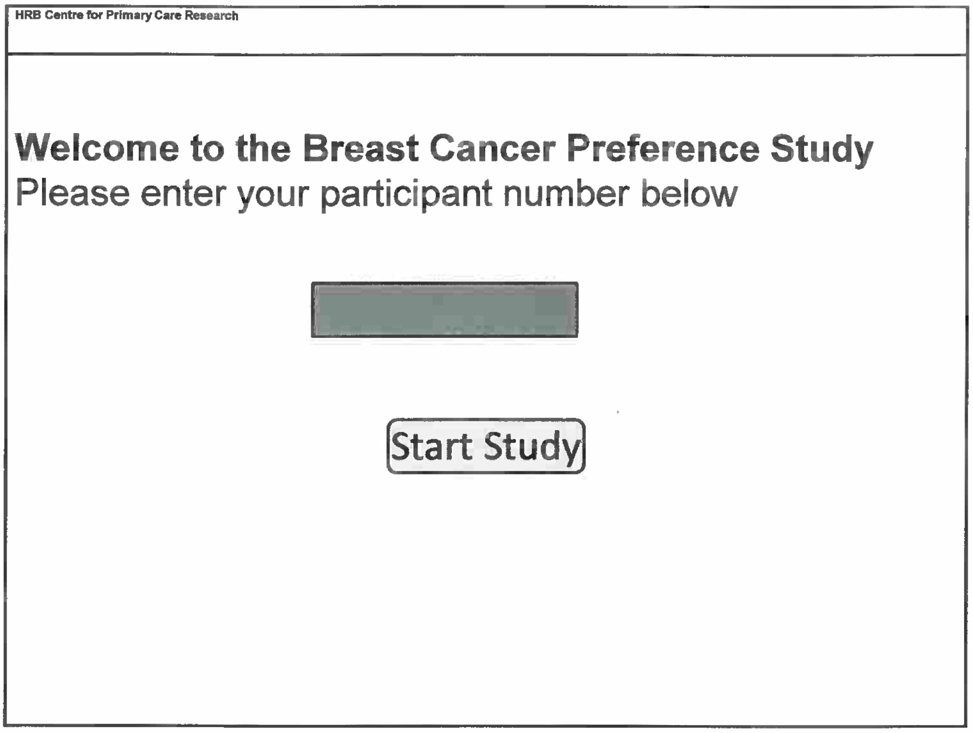
HRB Centre for Primary Care Research
Welcome to the Breast Cancer Preference Study
Please enter your participant number below
Start Study

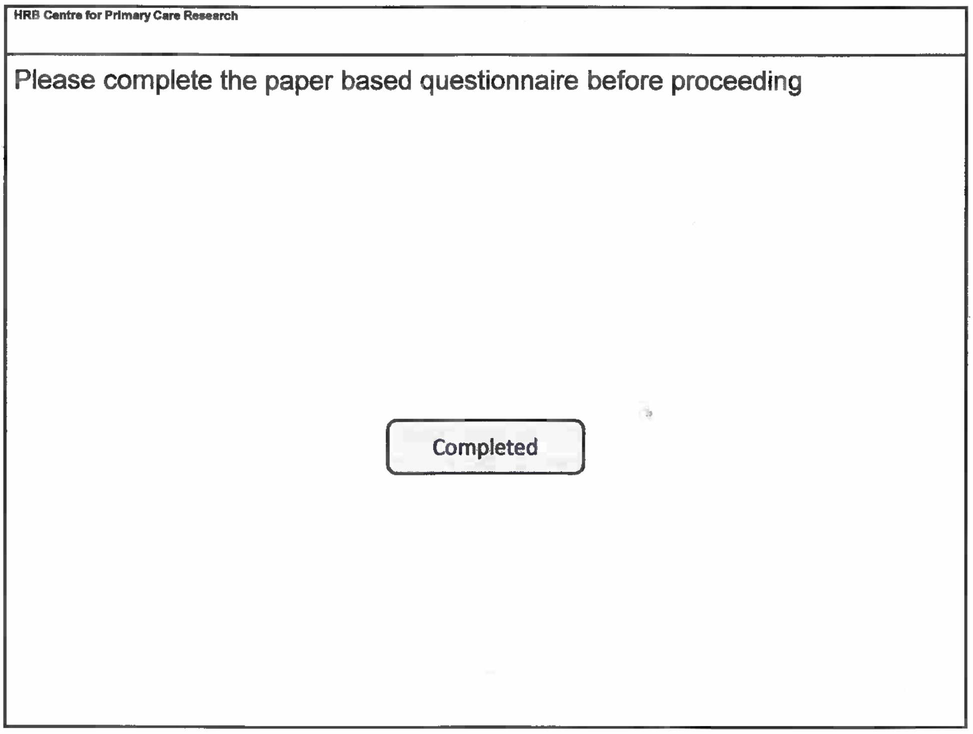
HRB Centre for Primary Care Research
Please complete the paper based questionnaire before proceeding
Completed

5/5/2015

HRB Centre for Primary Care Research

This is a study aimed at understanding womens' perceptions of breast cancer. Women with certain risk factors, signs and symptoms are more likely than others to develop breast cancer.

We will present you with several health scenarios, and ask you to choose between different states of health.

Next

Scenario

You have presented to your GP with symptom(s) of breast cancer.
Your GP will take a history of your symptoms, and perform a breast examination. They may also perform any other examinations/tests that they feel are necessary (listen to your heart, take blood, etc.).
Based on history and examination findings, your GP will either recommend a "watchful waiting" programme, or triple assessment at a specialist breast clinic.
However, it is your decision which option you prefer.

Definitions

Beside you are definitions for terms that you will come across in the scenarios. Please read them now, but you will also have a copy of them beside you to refer to at any point. Any word in **blue** in the scenarios has a definition provided.

5/5/2015

How will we measure your preferences?

We will use 2 methods (the VAS and standard gamble) to assess how you feel about each of the 11 health states. The health states will be presented to you in random order.

An explanation of each of the methods will follow. You also have laminated cards in front of you with the same information.

You can refer to these at any time that you wish.

Visual Analogue Scale - VAS

The Visual Analogue Scale is a scale from 0-100, with 0 being the worst health scenario you can think of (death), and 100 being the best health scenario (perfect health).

We will ask you to read each health care scenario individually, and then to place it where you think it fits along the scale.

You do not need even gaps between scenarios on the scale, and you can place two or more scenarios on the same number if you wish.

Scale: 0-100

Please include VAS scale here

36 peo[illegible]
Scen 1-5
Type Asse.
6-11
Watch Wi[illegible]

Standard Gamble.

You will be presented with 11 different health state scenarios, each scenario represents your current permanent health state. We will ask you to make a gamble between your current health state with two other health states (perfect health or death). I want you to imagine that this carries a certain chance of you living the rest of your life in perfect health and a certain chance of you dying painlessly tomorrow.

It is important to remember your current health scenario, and your current symptoms, treatments, side-effects and prognosis. You will be given different chances of perfect health versus death, against the current scenario, and keep gambling until you reach a point where you cannot choose between the 2 options.

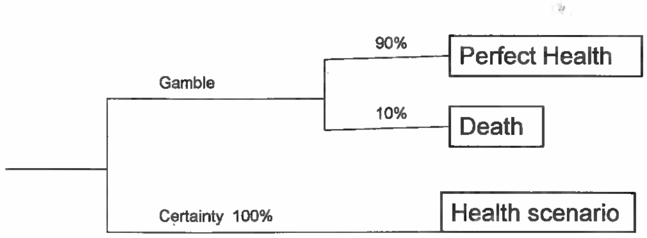

5/5/2015

Example- Standard Gamble

Imagine you have a heart condition. You have a choice between taking medication which has unpleasant side effects or taking no medication.

If you had to choose between taking this medication with side-effects for the rest of your life, or taking no medication and have perfect health, but with the risk of dying, what risk of death would you take to avoid the medication and side effects?

Another way of putting it is - what risk of dying painlessly tomorrow would you be willing to accept (if you didn't take the drugs) before you change your mind and decide you would rather take them?

•If offered a 90% chance of perfect health, no drugs, no side effects, but with a 10% risk of instant death, would you take it?

•If offered a 70% chance of perfect health, no drugs, no side effects, but with a 30% risk of instant death, would you take it?

Below, you will see 2 buttons. When you click on the right button, the chances of perfect health and death vary, these are examples to help you understand. In the text box below, please enter in the risk of death that you are willing to take.

no drugs, no side effects

Live in current Health State. — Versus — 50% chance of perfect health and 50% chance of death. instant

[ ] % chance of death

Enter

The Study

We are going to present you with 11 possible health scenarios in a random order - please follow the instructions and if you have any questions please ask.

Definitions have been provided for any word or phrase in blue.

5/5/2015

1,0069

Health State 1: Referred to breast clinic for triple assessment - Stage 1 Breast Cancer

What it means:
You were referred for **triple assessment** and breast cancer has been diagnosed that is confined to the breast and may involve a small number of **lymph nodes**. This is an early stage of breast cancer.

Physical Consequences:
- You have undergone **triple assessment.**
- You will have surgery (**lumpectomy, mastectomy, lymph node removal**).
- **Chemotherapy** and/or **radiation therapy** to prevent the cancer from coming back.
- **Hormonal therapy** or **biological therapy** may be given, depending on the type of cancer cell.

Emotional Consequences:
- Fear over investigations and treatments (pain, side-effects).
- Anxiety or depression associated with diagnosis.
- Worry that cancer may spread or will not be fully cured.

VAS Scale
Assume that you are currently experiencing the health state you just read. On a scale of 0 to 100 where 0 is death and 100 perfect health where would you rate this health state. Please use the scale below.
When you are happy with your selection please click 'Completed'.

Health State 1: Referred to breast clinic for triple assessment - Stage 1 Breast Cancer

What it means:
You were referred for **triple assessment** and breast cancer has been diagnosed that is confined to the breast and may involve a small number of **lymph nodes**. This is an early stage of breast cancer.

Physical Consequences:
- You have undergone **triple assessment.**
- You will have surgery (**lumpectomy, mastectomy, lymph node removal**).
- **Chemotherapy** and/or **radiation therapy** to prevent the cancer from coming back.
- **Hormonal therapy** or **biological therapy** may be given, depending on the type of cancer cell.

Emotional Consequences:
- Fear over investigations and treatments (pain, side-effects).
- Anxiety or depression associated with diagnosis.
- Worry that cancer may spread or will not be fully cured.

Standard Gamble
You have the opportunity to gamble your current health state (as described above) for perfect health, but you also risk death if you take the gamble. It is important to remember about your current health scenario, and your current symptoms, treatments, side-effects and prognosis. Below, you will see 2 buttons. When you click on the right button, the chances of perfect health and death vary, these are examples to help you understand. In the text box below, please enter in the risk of death that you are willing to take in order to avoid this health state.

\ type

5/5/2015

Health State 2: Referred to breast clinic for triple assessment - Stage 2 Breast Cancer

What it means:
Triple assessment has found cancer that is contained within the breast. It may also be in surrounding **lymph nodes**.

Physical Consequences:
• You have undergone **triple assessment.**
• You will have surgery (**lumpectomy, mastectomy, lymph node removal**).
• You will have **radiation therapy.**
• You may have **chemotherapy** and/or **hormonal therapy,** depending on the type of cancer.

Emotional Consequences:
• Fear over investigations and treatments (pain, side-effects).
• Anxiety or depression associated with diagnosis.
• Fear of recurrence.

VAS Scale
Assume that you are currently experiencing the health state you just read. On a scale of 0 to 100 where 0 is death and 100 perfect health where would you rate this health state. Please use the scale below. When you are happy with your selection please click 'Completed'.

Health State 2: Referred to breast clinic for triple assessment; Stage 2 Breast Cancer

What it means:
Triple assessment has found cancer that is contained within the breast. It may also be in surrounding **lymph nodes**.

Physical Consequences:
• You have undergone **triple assessment.**
• You will have surgery (**lumpectomy, mastectomy, lymph node removal**).
• You will have **radiation therapy.**
• You may have **chemotherapy** and/or **hormonal therapy,** depending on the type of cancer.

Emotional Consequences:
• Fear over investigations and treatments (pain, side-effects).
• Anxiety or depression associated with diagnosis.
• Fear of recurrence.

Standard Gamble
You have the opportunity to gamble your current health state (as described above) for perfect health, but you also risk death if you take the gamble. It is important to remember about your current health scenario, and your current symptoms, treatments, side-effects and prognosis. Below, you will see 2 buttons. When you click on the right button, the chances of perfect health and death vary, these are examples to help you understand. In the text box below, please enter in the risk of death that you are willing to take in order to avoid this health state.

For peer review only - http://bmjopen.bmj.com/site/about/guidelines.xhtml

5/5/2015

Health State 3: Referred to breast clinic for triple assessment - Stage 3 Cancer

What it means:
You were referred for **triple assessment** and cancer was found in the breast, it has spread into surrounding tissues and many **lymph nodes** are involved.

Physical consequences:
- You have undergone **triple assessment.**
- You will have a surgery (**lumpectomy, mastectomy, lymph node removal**).
- You will probably have **radiation therapy** and/or **chemotherapy.**
- You may have **hormonal** or **biological therapy**, depending on the type of cancer cells.

Emotional consequences:
- Fear over investigations and treatments (pain, side-effects).
- Poorer prognosis, worry about survival.
- Fear of recurrence.

VAS Scale
Assume that you are currently experiencing the health state you just read. On a scale of 0 to 100 where 0 is death and 100 perfect health where would you rate this health state. Please use the scale below. When you are happy with your selection please click 'Completed'.

Health State 3: Referred to breast clinic for triple assessment - Stage 3 Cancer

What it means:
You were referred for **triple assessment** and cancer was found in the breast, it has spread into surrounding tissues and many **lymph nodes** are involved.

Physical consequences:
- You have undergone **triple assessment.**
- You will have a surgery (**lumpectomy, mastectomy, lymph node removal**).
- You will probably have **radiation therapy** and/or **chemotherapy.**
- You may have **hormonal** or **biological therapy**, depending on the type of cancer cells.

Emotional consequences:
- Fear over investigations and treatments (pain, side-effects).
- Poorer prognosis, worry about survival .
- Fear of recurrence.

Standard Gamble
You have the opportunity to gamble your current health state (as described above) for perfect health, but you also risk death if you take the gamble. It is important to remember about your current health scenario, and your current symptoms, treatments, side-effects and prognosis. Below, you will see 2 buttons. When you click on the right button, the chances of perfect health and death vary, these are examples to help you understand. In the text box below, please enter in the risk of death that you are willing to take in order to avoid this health state.

5/5/2015

Health State 4: Referred to breast clinic for triple assessment - Stage 4 Breast Cancer found

What it means:
You are referred for **triple assessment**, and cancer is found in your breast, which has also spread to one or more organs in the body. At this stage the cancer cannot be cured, but treatment can slow down the disease and let you live longer.

Physical consequences:
- You have undergone **triple assessment**.
- The main treatment is **chemotherapy.**
- You may have surgery or **radiation therapy** to treat pain and other symptoms.
- Possible **hormonal and/or biological therapy**, depending on type of cancer.

Emotional consequences:
- Fear due to poor prognosis and treatments.
- Fear of dying.

VAS Scale
Assume that you are currently experiencing the health state you just read. On a scale of 0 to 100 where 0 is death and 100 perfect health where would you rate this health state. Please use the scale below. When you are happy with your selection please click 'Completed'.

Health State 4: Referred to breast clinic for triple assessment - Stage 4 Breast Cancer found

What it means:
You are referred for **triple assessment**, and cancer is found in your breast, which has also spread to one or more organs in the body. At this stage the cancer cannot be cured, but treatment can slow down the disease and let you live longer.

Physical consequences:
- You have undergone **triple assessment**.
- The main treatment is **chemotherapy.**
- You may have surgery or **radiation therapy** to treat pain and other symptoms.
- Possible **hormonal and/or biological therapy**, depending on type of cancer.

Emotional consequences:
- Fear due to poor prognosis and treatments.
- Fear of dying.

Standard Gamble
You have the opportunity to gamble your current health state (as described above) for perfect health, but you also risk death if you take the gamble. It is important to remember about your current health scenario, and your current symptoms, treatments, side-effects and prognosis. Below, you will see 2 buttons. When you click on the right button, the chances of perfect health and death vary, these are examples to help you understand. In the text box below, please enter in the risk of death that you are willing to take in order to avoid this health state.

5/5/2015

Health State 5: Referred to breast clinic for triple assessment - No cancer found

What it means:
You were referred for **triple assessment** and no cancer was found.

Physical consequences:
•You have undergone **triple assessment**.

Emotional consequences:
• Fear of investigations.
• Fear that the results were wrong and that you could still have cancer.

VAS Scale
Assume that you are currently experiencing the health state you just read. On a scale of 0 to 100 where 0 is death and 100 perfect health where would you rate this health state. Please use the scale below.
When you are happy with your selection please click 'Completed'.

Health State 5: Referred to breast clinic for triple assessment - No cancer found

What it means:
You were referred for **triple assessment** and no cancer was found.

Physical consequences:
•You have undergone **triple assessment**.

Emotional consequences:
•Fear of investigations
•Fear that the results might be wrong and that you could still have cancer.

Standard Gamble
You have the opportunity to gamble your current health state (as described above) for perfect health, but you also risk death if you take the gamble. It is important to remember about your current health scenario, and your current symptoms, treatments, side-effects and prognosis. Below, you will see 2 buttons. When you click on the right button, the chances of perfect health and death vary, these are examples to help you understand. In the text box below, please enter in the risk of death that you are willing to take in order to avoid this health state.

5/5/2015

Health State 6: Watchful waiting, recurrence/worsening of symptoms, referred to breast clinic for triple assessment - Stage 1 Breast Cancer found

What it means
After a period of **watchful waiting** with your GP, your symptoms were not resolving/becoming worse. You were referred for **triple assessment** and breast cancer has been diagnosed that is confined to the breast and may involve a small number of **lymph nodes**. This is an early stage of breast cancer.

Physical consequences:
- You have undergone **triple assessment**.
- You will have surgery (**lumpectomy, mastectomy, lymph node removal**).
- **Chemotherapy** and/or **radiation therapy** to prevent the cancer from coming back.
- **Hormonal therapy** or **biological therapy** may be given, depending on the type of cancer cells.

Emotional Consequences:
- Anger/worry about delay in diagnosis.
- Fear over investigations and treatments (pain, side-effects).
- Anxiety or depression associated with diagnosis.
- Worry that cancer may spread or will not be fully cured.

VAS Scale
Assume that you are currently experiencing the health state you just read. On a scale of 0 to 100 where 0 is death and 100 perfect health where would you rate this health state. Please use the scale below. When you are happy with your selection please click 'Completed

Health State 6: Watchful waiting, recurrence/worsening of symptoms, referred to breast clinic for triple assessment - Stage 1 Breast Cancer found

What it means
After a period of **watchful waiting** with your GP, your symptoms were not resolving/becoming worse. You were referred for **triple assessment** and breast cancer has been diagnosed that is confined to the breast and may involve a small number of **lymph nodes**. This is an early stage of breast cancer.

Physical consequences:
- You have undergone **triple assessment**.
- You will have surgery (**lumpectomy, mastectomy, lymph node removal**).
- **Chemotherapy** and/or **radiation therapy** to prevent the cancer from coming back.
- **Hormonal therapy** or **biological therapy** may be given, depending on the type of cancer cells.

Emotional Consequences:
- Anger/worry about delay in diagnosis.
- Fear over investigations and treatments (pain, side-effects).
- Anxiety or depression associated with diagnosis.
- Worry that cancer may spread or will not be fully cured

Standard Gamble
You have the opportunity to gamble your current health state (as described above) for perfect health, but you also risk death if you take the gamble. It is important to remember about your current health scenario, and your current symptoms, treatments, side-effects and prognosis. Below, you will see 2 buttons. When you click on the right button, the chances of perfect health and death vary, these are examples to help you understand. In the text box below, please enter in the risk of death that you are willing to take in order to avoid this health state.

10

5/5/2015

Health State 7: Watchful waiting, recurrence/worsening of symptoms, referred to breast clinic for triple assessment - Stage 2 Breast Cancer found

What it means:
After a period of **watchful waiting**, your symptoms were not resolving/became worse. You were referred for **triple assessment**, and cancer was found, which is contained within the breast. It may also be in surrounding **lymph nodes.**

Physical consequences:
- You have undergone **triple assessment**.
- You will have surgery (**lumpectomy, mastectomy, lymph node removal**).
- You will have **radiation therapy.**
- You may have **chemotherapy**, and/or **hormonal therapy,** depending on the type of cancer

Emotional consequences:
- Anger/worry about delay in diagnosis.
- Fear over investigations and treatments (pain, side-effects).
- Anxiety or depression due to cancer diagnosis.
- Fear of recurrence.

VAS Scale
Assume that you are currently experiencing the health state you just read. On a scale of 0 to 100 where 0 is death and 100 perfect health where would you rate this health state. Please use the scale below.
When you are happy with your selection please click 'Completed

Health State 7: Watchful waiting, recurrence/worsening of symptoms, referred to breast clinic for triple assessment - Stage 2 Breast Cancer found

What it means:
After a period of **watchful waiting**, your symptoms were not resolving/became worse. You were referred for **triple assessment**, and cancer was found, which is contained within the breast. It may also be in surrounding **lymph nodes.**

Physical consequences:
- You have undergone **triple assessment**.
- You will have surgery (**lumpectomy, mastectomy, lymph node removal**).
- You will have **radiation therapy.**
- You may have **chemotherapy**, and/or **hormonal therapy,** depending on the type of cancer.

Emotional consequences:
- Anger/worry about delay in diagnosis.
- Fear over investigations and treatments (pain, side-effects).
- Anxiety or depression due to cancer diagnosis.
- Fear of recurrence.

Standard Gamble
You have the opportunity to gamble your current health state (as described above) for perfect health, but you also risk death if you take the gamble. It is important to remember about your current health scenario, and your current symptoms, treatments, side-effects and prognosis. Below, you will see 2 buttons. When you click on the right button, the chances of perfect health and death vary, these are examples to help you understand. In the text box below, please enter in the risk of death that you are willing to take in order to avoid this health state.

5/5/2015

Health State 8: Watchful waiting, recurrence/worsening of symptoms, referred to breast clinic for triple assessment - Stage 3 Breast Cancer found

What it means:
After a period of **watchful waiting**, your symptoms have not resolved/become worse. You are referred for **triple assessment**. Cancer is found in the breast, it has spread into surrounding tissues, and many **lymph nodes** are involved.

Physical consequences:
- You have undergone **triple assessment.**
- You will have a surgery (**lumpectomy, mastectomy, lymph node removal**).
- You will probably have **radiation therapy** and/or **chemotherapy.**
- You may have **hormonal** or **biological therapy**, depending on the type of cancer cells.

Emotional consequences:
- Anger/worry about delay in diagnosis.
- Fear over investigations and treatments (pain, side-effects).
- Poorer prognosis, worry about survival.
- Fear of recurrence.

VAS Scale
Assume that you are currently experiencing the health state you just read. On a scale of 0 to 100 where 0 is death and 100 perfect health where would you rate this health state. Please use the scale below. When you are happy with your selection please click 'Completed

Health State 8: Watchful waiting, recurrence/worsening of symptoms, referred to breast clinic for triple assessment - Stage 3 Breast Cancer found

What it means:
After a period of **watchful waiting**, your symptoms have not resolved/become worse. You are referred for **Triple assessment**. Cancer is found in the breast, it has spread into surrounding tissues, and many **lymph nodes** are involved.

Physical consequences:
- You have undergone **triple assessment.**
- You will have a surgery (**lumpectomy, mastectomy, lymph node removal**).
- You will probably have **radiation therapy** and/or **chemotherapy.**
- You may have **hormonal** or **biological therapy**, depending on the type of cancer cells.

Emotional consequences:
- Anger/worry about delay in diagnosis.
- Fear over investigations and treatments (pain, side-effects).
- Poorer prognosis, worry about survival.
- Fear of recurrence.

Standard Gamble
You have the opportunity to gamble your current health state (as described above) for perfect health, but you also risk death if you take the gamble. It is important to remember about your current health scenario, and your current symptoms, treatments, side-effects and prognosis. Below, you will see 2 buttons. When you click on the right button, the chances of perfect health and death vary, these are examples to help you understand. In the text box below, please enter in the risk of death that you are willing to take in order to avoid this health state.

For peer review only - http://bmjopen.bmj.com/site/about/guidelines.xhtml

Health State 9: Watchful waiting, recurrence/worsening of symptoms, referred to breast clinic for triple assessment - Stage 4 Breast Cancer found

What it means:
After a period of **watchful waiting** with your GP, your symptoms have not resolved/became worse. You are referred for **triple assessment**, and cancer is found in your breast, which has also spread to one or more organs in the body. At this stage the cancer cannot be cured, but treatment can slow down the disease and let you live longer.

Physical consequences:
- You have undergone **triple assessment**.
- The main treatment is **chemotherapy.**
- You may have surgery or **radiation therapy** to treat pain and other symptoms.
- Possible **hormonal** and/or **biological therapy**, depending on type of cancer.

Emotional Consequences:
- Anger/worry about delay in diagnosis.
- Fear due to poor prognosis and treatments.
- Fear of dying.

VAS Scale
Assume that you are currently experiencing the health state you just read. On a scale of 0 to 100 where 0 is death and 100 perfect health where would you rate this health state. Please use the scale below. When you are happy with your selection please click 'Completed'.

Health State 1
0 ——|—————————————— 100

Health State 9: Watchful waiting, recurrence/worsening of symptoms, referred to breast clinic for triple assessment - Stage 4 Breast Cancer found

What it means:
After a period of **watchful waiting** with your GP, your symptoms have not resolved/became worse. You are referred for **triple assessment**, and cancer is found in your breast, which has also spread to one or more organs in the body. At this stage the cancer cannot be cured, but treatment can slow down the disease and let you live longer.

Physical consequences:
- You have undergone **triple assessment**.
- The main treatment is **chemotherapy.**
- You may have surgery or **radiation therapy** to treat pain and other symptoms.
- Possible **hormonal** and/or **biological therapy**, depending on type of cancer.

Emotional Consequences:
- Anger/worry about delay in diagnosis.
- Fear due to poor prognosis and treatments.
- Fear of dying.

Standard Gamble.
You have the opportunity to gamble your current health state (as described above) for perfect health, but you also risk death if you take the gamble. It is important to remember about your current health scenario, and your current symptoms, treatments, side-effects and prognosis. Below, you will see 2 scenarios. When you click on the right button, the chances of perfect health and death vary, these are examples to help you understand. In the text box below, please enter in the risk of death that you are prepared to take in order to avoid this health state.

5/5/2015

Health State 10: Watchful waiting, referred to breast clinic for triple assessment - No cancer found

What it means
After a period **watchful waiting** with your GP. Your symptoms were not resolving/became worse. You are referred for **triple assessment** and no cancer was found.

Physical Consequences:
- You have undergone **triple assessment.**

Emotional Consequences:
- Fear of investigations.
- Worry about the delay in diagnosis.
- Fear the results might be wrong and that you still could have cancer.

VAS Scale
Assume that you are currently experiencing the health state you just read. On a scale of 0 to 100 where 0 is death and 100 perfect health where would you rate this health state. Please use the scale below. When you are happy with your selection please click 'Completed'.

Health State 10 : Watchful waiting, referred to breast clinic for triple assessment - No cancer found

What it means
After a period **watchful waiting** with your GP. Your symptoms were not resolving/became worse. You are referred for **triple assessment** and no cancer was found.

Physical Consequences:
- You have undergone **triple assessment.**

Emotional Consequences:
- Fear of investigations.
- Worry about the delay in diagnosis.
- Fear the results may be wrong and that you still could have cancer.

Standard Gamble.
You have the opportunity to gamble your current health state (as described above) for perfect health, but you also risk death if you take the gamble. It is important to remember about your current health scenario, and your current symptoms, treatments, side-effects and prognosis. Below, you will see 2 buttons. When you click on the right button, the chances of perfect health and death vary, these are examples to help you understand. In the text box below, please enter in the risk of death that you are willing to take in order to avoid this health state.

5/5/2015

Health State 11: Watchful waiting, resolution of symptoms, no referral needed.

What it means:
After a period of **watchful waiting**, symptoms have stopped or are resolving within 3 month's follow up with GP. You do not need further investigation.

Physical Consequences:
•.No further tests are needed.

Emotional Consequences:
• Relief that symptoms are not serious.
• Worry that there might be a cancer that has been missed.

VAS Scale
Assume that you are currently experiencing the health state you just read. On a scale of 0 to 100 where 0 is death and 100 perfect health where would you rate this health state. Please use the scale below. When you are happy with your selection please click 'Completed'.

Health State 11: Watchful waiting, resolution of symptoms, no referral needed.

What it means:
After a period of **watchful waiting**, symptoms have stopped or are resolving within 3 month's follow up with GP. You do not need further investigation.

Physical Consequences:
•.No further tests are needed.

Emotional Consequences:
• Relief that symptoms are not serious.
• Worry that there might be a cancer that has been missed.

Standard Gamble.

You have the opportunity to gamble your current health state (as described above) for perfect health, but you also risk death if you take the gamble. It is important to remember about your current health scenario, and your current symptoms, treatments, side-effects and prognosis. Below, you will see 2 buttons. When you click on the right button, the chances of perfect health and death vary, these are examples to help you understand. In the text box below, please enter in the risk of death that you are willing to take in order to avoid this health state.

STROBE Statement—checklist of items that should be included in reports of observational studies

	Item No	Recommendation
**Title and abstract**	1	Decision analysis mentioned in title and abstract
		(*b*) Provide in the abstract an informative and balanced summary of what was done and what was found- see abstract
**Introduction**		
Background/rationale	2	Explain the scientific background and rationale for the investigation being reported
Objectives	3	State specific objectives, including any prespecified hypotheses<br>See pages 4-8
**Methods**		
Study design	4	Present key elements of study design early in the paper- page 4-8
Setting	5	Describe the setting, locations, and relevant dates, including periods of recruitment, exposure, follow-up, and data collection- page 4-8
Participants	6	Page 4-8
Variables	7	Details of decision tree and utility states in methods pages 4-8 and in supplementary file of health states computerized programme that performed utility assessment
Data sources/ measurement	8*	Table 1
Bias	9	Limitations discussed page 11
Study size	10	Explanation re: feasibility study in methods section page 7
Quantitative variables	11	Explanation, methods page 6-8
Statistical methods	12	Plan of analysis pages 6-8; includes sensitivity analysis

Continued on next page

For peer review only

For peer review only - http://bmjopen.bmj.com/site/about/guidelines.xhtml

**Results**		
Participants	13*	Page 9
Descriptive data	14*	Table 2
Main results	16	Page 9, Table 3, Figure 2
**Discussion**		
Key results	18	Page 10-11
Limitations	19	Page 11
Interpretation	20	Page 11-12
Generalisability	21	Page 11-12
**Other information**		
Funding	22	Page 13 Give the source of funding and the role of the funders for the present study and, if applicable, for the original study on which the present article is based

For peer review only

For peer review only - http://bmjopen.bmj.com/site/about/guidelines.xhtml
